# Novel Quaternary Ammonium Salt‐Linked STING Agonist Antibody‐Drug Conjugate: Synergistic Activation of Tumor Immunity with Mitigated Off‐Target Toxicity

**DOI:** 10.1002/advs.202502270

**Published:** 2025-06-10

**Authors:** Yu Long, Borui Tang, Fei Xie, Lianqi Liu, Yangyihua Zhou, Jingwen Dong, Jianfeng Wang, Cuicui Sun, Yuting Wang, Ruoqi Li, Na Zhang, Liping Li, Longlong Luo, Junhai Xiao, Wu Zhong, Dian Xiao, Hongbin Deng, Xinbo Zhou

**Affiliations:** ^1^ College of Pharmacy Qingdao University Qingdao 266071 China; ^2^ School of Pharmaceutical Sciences Capital Medical University Beijing 100069 China; ^3^ National Engineering Research Center for the Emergency Drug Academy of Military Medical Sciences Beijing 100850 China; ^4^ Institute of Medicinal Biotechnology Chinese Academy of Medical Sciences & Peking Union Medical College Beijing 100050 China; ^5^ Academy of Military Medical Sciences Beijing 100850 China

**Keywords:** antitumor immunity, diABZI STING agonist 3, immune‐stimulating antibody conjugates, quaternary ammonium salt‐linker, stimulator of interferon genes

## Abstract

Immune‐stimulating antibody conjugate (ISACs) incorporating STING agonists as payloads leverage both the targeting capability of the Fab region and the Fc region‐mediated tumor antigen‐dependent immune activation. Herein, a novel class of ISACs is reported, generated by engineering a quaternary ammonium‐cleavable linker to conjugate diABZI STING agonist 3 (dSA3) with the HER2‐targeting antibody Trastuzumab. The optimized ISAC (TZ‐dSA3‐12) demonstrated high potency, stability, enhanced solubility, and reduced off‐target toxicity. The data showed that TZ‐dSA3‐12 potently activates the STING pathway in the tumor microenvironment through the synergistic action of the Fab and Fc regions of antibodies (*activity switch‐on*). In contrast, TZ‐dSA3‐12 exhibited ≈75 fold lower activity than dSA3 in normal immune cells, where activation relies solely on the Fc region without Fab‐mediated tumor antigen binding (*activity switch‐off*). Furthermore, systemic administration of TZ‐dSA3‐12 at a dose (1 mg kg^−1^) elicited robust and sustained antitumor effect in a manner dependent on the activation of innate immunity and adaptive immunity, including macrophages, dendritic cells (DCs) and CD8^+^ T cells, while minimizing systemic cytokine release. Notably, TZ‐dSA3‐12 also induced immunological memory to combat the growth of rechallenged tumors. This innovative quaternary ammonium‐linked STING agonist‐ISAC represents a promising avenue for the future development of STING‐targeted immunotherapy.

## Graphical abstract

Novel quaternary ammonium salt‐linked STING agonist ADC demonstrates high potency, stability, enhanced solubility, and reduced off‐target toxicity, potently activating the STING pathway in the tumor microenvironment through the synergistic action of the Fab and Fc regions of antibodies (activity switch‐on) while exhibiting ≈20 fold lower activity in normal immune cells, where activation relies solely on Fc (activity switch‐off).

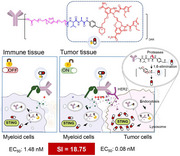

## 1 Introduction

Antibody‐drug conjugates (ADCs) are a category of therapeutics that consist of monoclonal antibodies conjugated with a cytotoxic payload via a specialized linker.^[^ ^1^ ^]^ The successful development of DS‐8201^[^ ^2^, ^3^ ^]^ has ushered in a new era of “targeted conjugation”, particularly with the emergence of immune‐stimulating antibody conjugates (ISACs) as a promising next‐generation of therapeutic ADC.^[^ ^4^, ^5^ ^]^ ISACs are meticulously designed to enable the covalent attachment of an immune agonist to a tumor‐targeting antibody,^[^ ^4^ ^]^ thereby not only activating innate immunity but also stimulating adaptive immunity, which collectively contributes to a dual therapeutic effect to eliminate tumor cells.^[^ ^5^ ^]^ However, ISACs remain in the early stage of concept validation, necessitating the development of more efficacious designs to overcome current limitations.

The pivotal role of the cGAS‐STING pathway in tumor immunity has established it as a promising target for the development of therapeutics designed to enhance the efficacy of cancer treatments.^[^ ^6^ ^]^ Under normal physiological conditions, STING is activated by its natural agonist, cGAMP, which is generated by the pattern recognition receptor cGAS in response to cytosolic dsDNA.^[^ ^7^ ^]^ As a second messenger, cGAMP binds to STING, leading to the activation of TANK‐binding kinase 1 (TBK1) and inhibitor of κB kinases (IKKs) signaling pathways.^[^ ^8^ ^]^ This activation subsequently triggers the transcription factors interferon regulatory factor 3 (IRF3) and nuclear factor κB (NF‐κB)‐dependent production of type I interferons (IFNs) and other inflammatory cytokines/chemokines, which promote antitumor immunity by activating T cells and natural killer (NK) cells.^[^ ^9^ ^]^ Given the immunostimulatory and antitumor effects of the cGAS‐STING pathway, substantial efforts have been directed to target this pathway pharmaceutically. At present, nine STING agonists are in clinical trials, including cyclic dinucleotides (CDN) derivatives (e.g., ADU‐S100^[^ ^10^ ^]^) and non‐CDN agonists (e.g., MSA‐2,^[^ ^11^ ^]^ SR717,^[^ ^12^ ^]^ and diABZI^[^ ^13^ ^]^). However, the overall clinical progress is relatively slow, with most agents restricted to intratumoral administration.^[^ ^14^ ^]^ ADU‐S100 was discontinued due to insufficient clinical efficacy,^[^ ^15^ ^]^ while the promising diABZI STING agonist 3 (dSA3)—despite showing potent STING activation and significant in vivo antitumor effects^[^ ^13^ ^]^—has remained stalled in Phase I trials, likely due to systemic toxicity risks such as cytokine storms.^[^ ^16^ ^]^

Recent advances have introduced STING agonist‐ISACs as a novel strategy to selectively target tumors while minimizing off‐target immune activation in normal tissues.^[^ ^17^ ^]^ Few studies focusing on STING agonist‐ISACs have been reported. One STING agonist‐ISAC, α‐EGFR‐172 ADC, utilized the STING agonist IMSA172 linked via a cathepsin‐cleavable valine‐citrulline (Val‐Cit) linker^[^ ^18^ ^]^ (Figure S1a, Supporting Information). However, IMSA172 demonstrated limited efficacy as a payload, with a half‐maximal effective concentration (EC_50_) of merely 35 µM for inducing an interferon response. Another STING agonist‐ISAC, XMT‐2056, conjugated the potent non‐CDN STING agonist XMT‐1616 to the HER2‐targeting antibody Trastuzumab via a cleavable ester linker^[^ ^19^ ^]^ (Figure S1b, Supporting Information). Although XMT‐2056 demonstrated significant antitumor activity in preclinical studies,^[^ ^20^ ^]^ its unstable linker contributed to Grade 5 adverse events and patient deaths in Phase I trials, leading to FDA‐mandated suspension. These findings indicate that while STING agonist‐ISACs hold promise as a therapeutic strategy, further structural optimization is essential to achieve an appropriate balance between therapeutic efficacy and safety.

**Figure 1:**
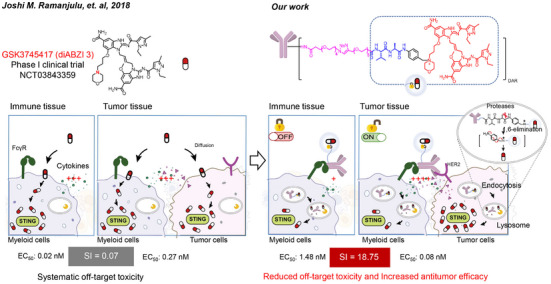
The proposed model of diABZI STING agonist 3 (dSA3) and STING ISAC eliciting durable antitumor immunity. In normal environments, the efficacy of dSA3 activity was superior to that of STING ISAC. However, within the tumor microenvironment, the activity of the STING ISAC was significant enhanced, exhibiting a synergistic effect that may mitigate the toxicity associated with systemic administration of dSA3.

In this study, we designed a novel STING agonist‐ISAC by conjugating the highly potent and stable dSA3 to Trastuzumab via a quaternary ammonium linker. The optimized construct, TZ‐dSA3‐12, exhibited exceptional stability, water solubility, and structural simplicity. The quaternary ammonium linker effectively shielded dSA3 activity and reduced off‐target toxicity in normal tissues. Meanwhile, TZ‐dSA3‐12 successfully delivered dSA3 to tumor sites and robustly synergistically activated STING signaling in both tumor cells and myeloid cells through the Fab and Fc regions of antibodies. This activation leads to an increased expression of interferon‐stimulated genes and pro‐inflammatory cytokines at therapeutically relevant nanomolar concentrations, which are significantly lower than free dSA3 (**Figure** **1**). Moreover, systemic administration of TZ‐dSA3‐12 elicited sustained antitumor immunity with minimal toxicity in a mouse tumor model. This innovative quaternary ammonium‐linked STING agonist‐ISAC represents a promising advancement in targeted STING agonist therapeutics.

## 2 Results

### 2.1 Design of a Quaternary Ammonium‐Linked STING Agonist Antibody Conjugate

**Figure 2:**
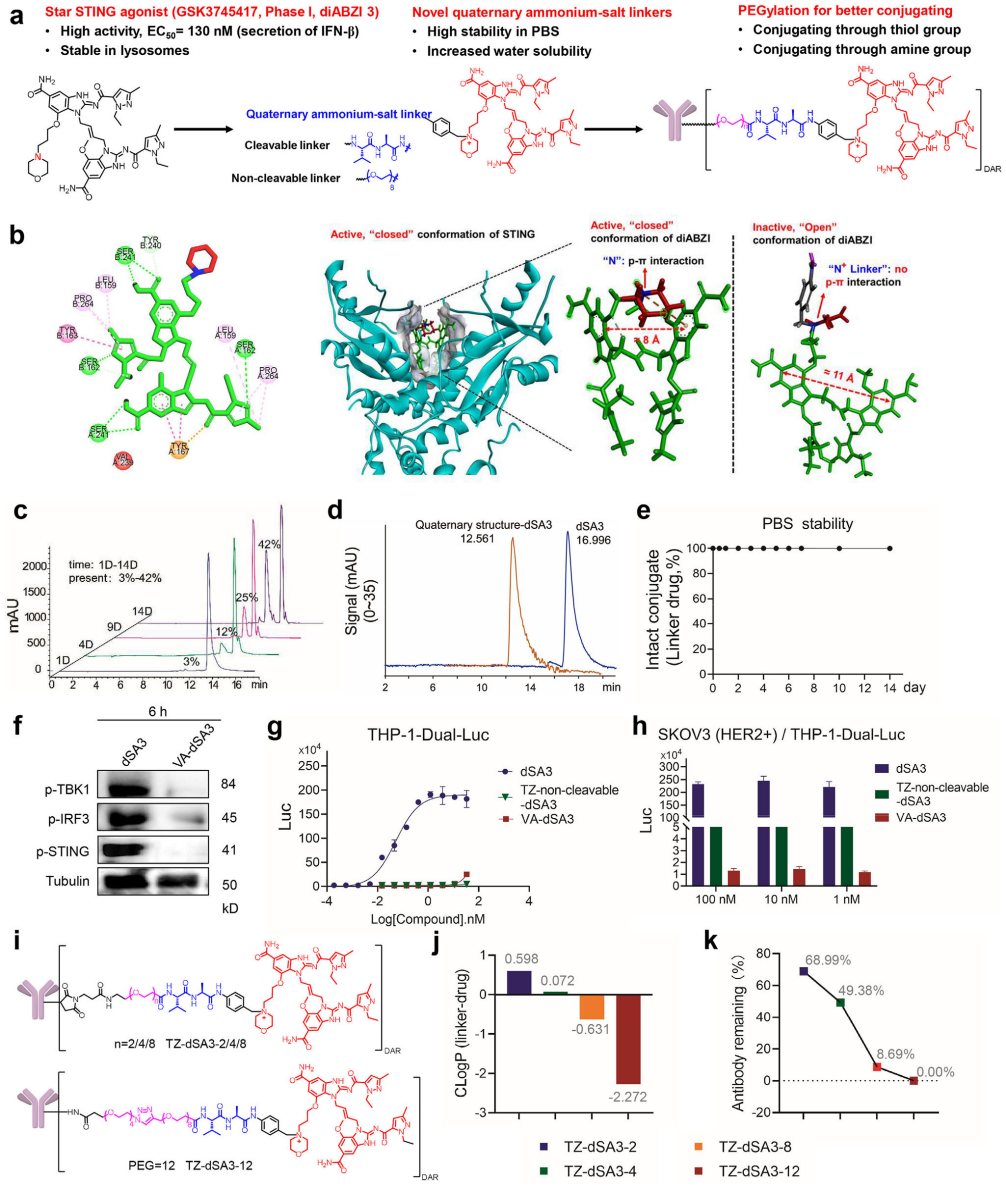
Design of a quaternary ammonium‐linked STING agonist immune‐stimulating antibody conjugate. a) Schematic diagram of the process of diABZI STING agonist 3 and linking to design qualified STING ISACs. b) Close‐up of dSA3 and non‐cleavable linker binding in the ligand‐binding pocket of STING. c) Reaction process detection diagram for the synthesis of VA‐dSA3. Reaction processes were monitored by high performance liquid chromatography (HPLC). d) HIC spectroscopies of dSA3 and Quaternary structure. e) VA‐dSA3 was dissolved in PBS over a time course as indicated. The release of dSA3 in PBS was detected by HPLC. f) E0771‐HER2 cells were treated with dSA3 (100 nM) and VA‐dSA3 (100 nM) for 6 h; the levels of phospho‐TBK1(p‐TBK1), phospho‐IRF3 (p‐IRF3), and phospho‐STING (p‐STING) were determined by western blot. g) Serial dilutions of dSA3, TZ‐non‐cleavable‐dSA3, or VA‐dSA3 were incubated with THP‐1‐Dual‐Luc cells for 24 h, and the interferon response was measured by luciferase assay. h) Indicated concentrations of dSA3, TZ‐non‐cleavable‐dSA3, and VA‐dSA3 were incubated with THP‐1‐Dual‐Luc cells, followed by co‐culturing with SKOV3 (HER2) cells for 24 h, the interferon response was measured by luciferase assay. i) Chemical structures of TZ‐dSA3 with different PEG linkers. j) ClogP analysis of linker‐drugs. the hydrophilicity of linker drugs was predicted through ChemDraw using ClogP. k) TZ‐dSA3 were conjugated with different PEG linkers, the remaining antibodies were measured by HIC analysis. For g, h) data were represented three independent experiments as mean ± SD (n = 3). EC_50_ values were derived using the curve fitting function in GraphPad Prism 9.0.

The non‐CDN STING agonist dSA3 is a promising candidate for ISAC payload due to its advantageous properties. Notably, dSA3 is the most potent STING agonist identified to date, exhibiting an EC_50_ of 130 nM in the induction of interferon (IFN) secretion. Its stability within lysosomal environments and favorable lipophilicity (LogD = 1.27) further enhance its potential for bystander effects (**Figure** **2**a). To rational design the linker for dSA3, we first performed molecular docking of dSA3 with the STING protein complex (PDB: 8GT6) and analyzed their interactions (Figure 2b). The binding model revealed that the symmetrical diABZI skeleton bound to the ligand‐binding domain (LBD) through extensive hydrophobic interactions and hydrogen bonds, while the morpholine ring projects outward from the binding pocket without significant contact with STING. Importantly, the nitrogen atom of the morpholine ring serves as the sole available site for linker conjugation. Therefore, we introduced linkers into morpholine to generate quaternary ammonium salt‐based linker‐drugs and corresponding ISACs. To assess the impact of linker modification on activity, we designed two linker types: a cathepsin B (CTSB)‐cleavable valine‐alanine (Val‐Ala) linker and a non‐cleavable polyethylene glycol (PEG) linker T (Figure 2a). Previous studies have shown that minor structural modifications to diABZI can drastically alter its activity.^[^ ^21^ ^]^ As illustrated in Figure 2c, the synthesis of the quaternary ammonium‐linked dSA3 (e.g., VA‐dSA3) was a stepwise process requiring ≈14 days. Hydrophobic interaction chromatography (HIC) confirmed that VA‐dSA3 exhibited greater hydrophilicity than dSA3 (Figure 2d). Additionally, VA‐dSA3 showed minimal payload release (<1.2%) over 14 days in phosphate‐buffered saline (PBS), indicating excellent stability (Figure 2e). Western blot results revealed that treatment with VA‐dSA3 did not increase phosphorylation levels of TBKI, IRF3, and STING (Figure 2f). We next evaluated the effect of ISAC and VA‐dSA3 on STING activation using a human monocytic THP‐1 cell line harboring a luciferase reporter gene under the control of NF‐κB and IRF3 promoter (THP‐1‐Dual‐Luc). Neither the non‐cleavable ISAC nor VA‐dSA3 activated the STING pathway in THP‐1‐Dual‐Luc reporter cells or SKOV3/THP‐1‐Dual‐Luc co‐culture systems (Figure 2g, h). These findings suggest that a non‐cleavable linker is unsuitable for dSA3, likely because the quaternary ammonium linker disrupts the critical p‐π conjugation between the nitrogen atom and the dSA3 benzene ring, shifting STING into an inactive “open” conformation (Figure 2b). Although the quaternary ammonium linker enhanced the hydrophilicity of dSA3, the addition of polyethylene glycol (PEG) remained necessary for efficient antibody conjugation (Figure 2i; Figure S1c–e, Supporting Information). As shown in Figure 2j, the extension of PEG linkers significantly improved the hydrophilicity of the linker‐dSA3 and markedly increased the conjugation efficiency. Ultimately, the STING ISAC utilizing a PEG12 linker achieved ≈100% conjugation efficiency (Figure 2k). Therefore, the ISAC featuring a PEG12 cleavable Val‐Ala quaternary ammonium linker, designated as TZ‐dSA3‐12, was selected for the subsequent research.

### 2.2 TZ‐dSA3‐12 Meets ISAC Quality Control Standards

**Figure 3:**
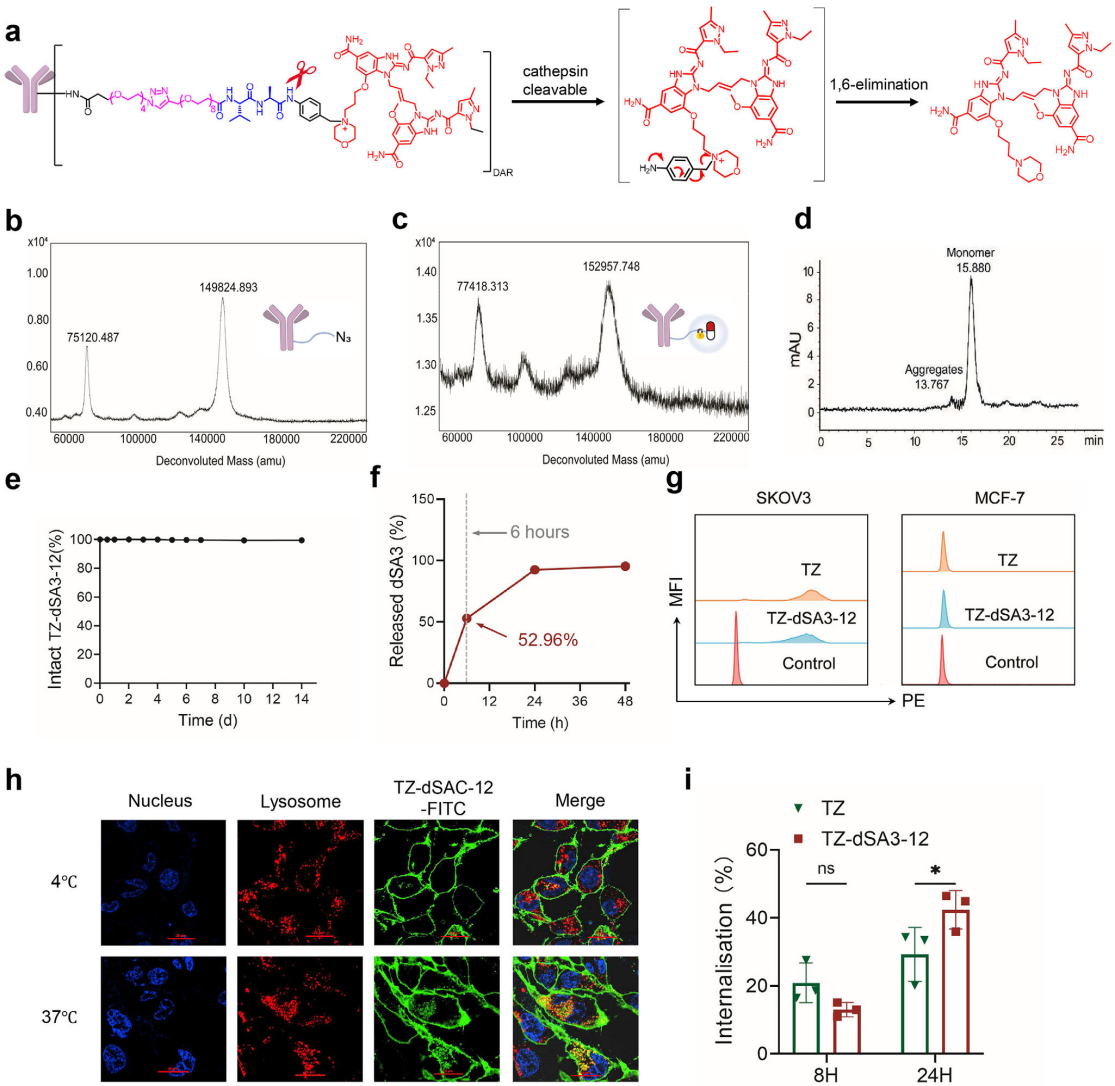
TZ‐dSA3‐12 meets the quality control standards for ISACs. a) Reaction scheme for the synthesis of TZ‐dSA3‐12 used in this study. b–d) MALDI‐TOF analysis of TZ‐dSA3‐12 (b) compared with TZ‐PEG4‐N3(c), SEC spectroscopies (d), e) The stability of TZ‐dSA3‐12 in human sera. TZ‐dSA3‐12 was incubated with 50% human sera at 37°C over the indicated time course. The released dSA3 in sera was measured by HPLC and presented as percentage of untreated samples. f) TZ‐dSA3‐12 were incubated with cathepsin B under 37°C for the indicated time. The released dSA3 was measured by HPLC. g) Flow cytometry analysis of TZ and TZ‐dSA3‐12 binding to HER2 antigens expressed by SKOV3 and MCF‐7 cells. h) Confocal microscopy analysis of TZ‐dSA3‐12 internalization and colocalization with lysosomes in SKOV3 cells at 4°C or 37°C. scale bar = 20 µm. TZ‐dSA3‐12 were labelled with FITC, lysosome was labelled with Lysotracker red, and nucleus was labelled with DAPI. i) Flow cytometry determination of the internalization rate of TZ‐dSA3‐12 and TZ in SKOV3 cells. For e, f, i) data were represented three independent experiments as mean ± SD (n = 3). To compare the difference in activity between TZ and TZ‐dSA3‐12, unpaired t test was used, and statistical analyses were performed using GraphPad Prism 9.0. ^*^ *p* < 0.05, ns, not significant.

Plasma stability and tumor‐specific cleavage efficiency of ISAC are critical for evaluating the quaternary ammonium salt‐linker design (**Figure** **3**a). First, Matrix‐assisted laser desorption/ionization time‐of‐flight (MALDI‐TOF) revealed that TZ‐dSA3‐12 contains an average of 2.1 drug molecules per antibody compared to TZ‐PEG4‐N3 (Figure 3b,c; Figure S1f, Supporting Information). Size exclusion chromatography (SEC) analysis indicated that TZ‐dSA3‐12 is predominantly composed of a single monomeric species, exhibiting over 96% purity (Figure 3d). Furthermore, TZ‐dSA3‐12 demonstrated a release of less than 1.5% of the total dSA3 payload over a 14‐day period in human plasma (Figure 3e), suggesting favorable plasma stability. In vitro studies revealed that in the presence of CTSB enzymes, TZ‐dSA3‐12 can release 52.96% of the drug within 6 hours and achieve a complete release in 24 h (Figure 3f), thereby confirming its efficient cleavage capacity. Subsequent assays utilizing phycoerythrin (PE)‐labeled TZ and TZ‐dSA3‐12 exhibited significant fluorescence in HER2‐positive SKOV3 cells, while displaying weak fluorescence in HER2‐negative MCF‐7 cells, indicating that TZ‐dSA3‐12 specifically targets the HER2 antigen present on cancer cells (Figure 3g). Additionally, the internalization and migration of TZ‐dSA3‐12 in SKOV3 cells were assessed using laser scanning confocal microscopy. Following the confirmation of TZ‐dSA3‐12 binding to the cell surface at 4 °C, the cells were incubated at 37 °C to facilitate internalization. Microscopic analysis revealed that the intracellular antibody signal colocalized with the lysosomal signal (Figure 3h), indicating successful internalization and translocation of TZ‐dSA3‐12 to the lysosome. Furthermore, the internalization of TZ‐dSA3‐12 in SKOV3 cells occurred as early as 4 h (Figure S2a, Supporting Information). Flow cytometry assay also revealed that the internalization rate of TZ‐dSA3‐12 was higher than that of TZ, with values of 41.4% compared to 31.1% at the 24 h (Figure 3i; Figure S2b, Supporting Information). Most importantly, free dSA3 possesses the advantage of small molecular weight, and enough lipid solubility (ClogP = 2.48), facilitating dSA3 escaping into the cytoplasm by free diffusion. Collectively, TZ‐dSA3‐12 meets all established criteria for a qualified ISAC, demonstrating robust plasma stability and efficient tumor‐specific drug release.

### 2.3 TZ‐dSA3‐12 Potently Stimulates the STING Signaling in HER2‐Expressing Cancer Cells

**Figure 4:**
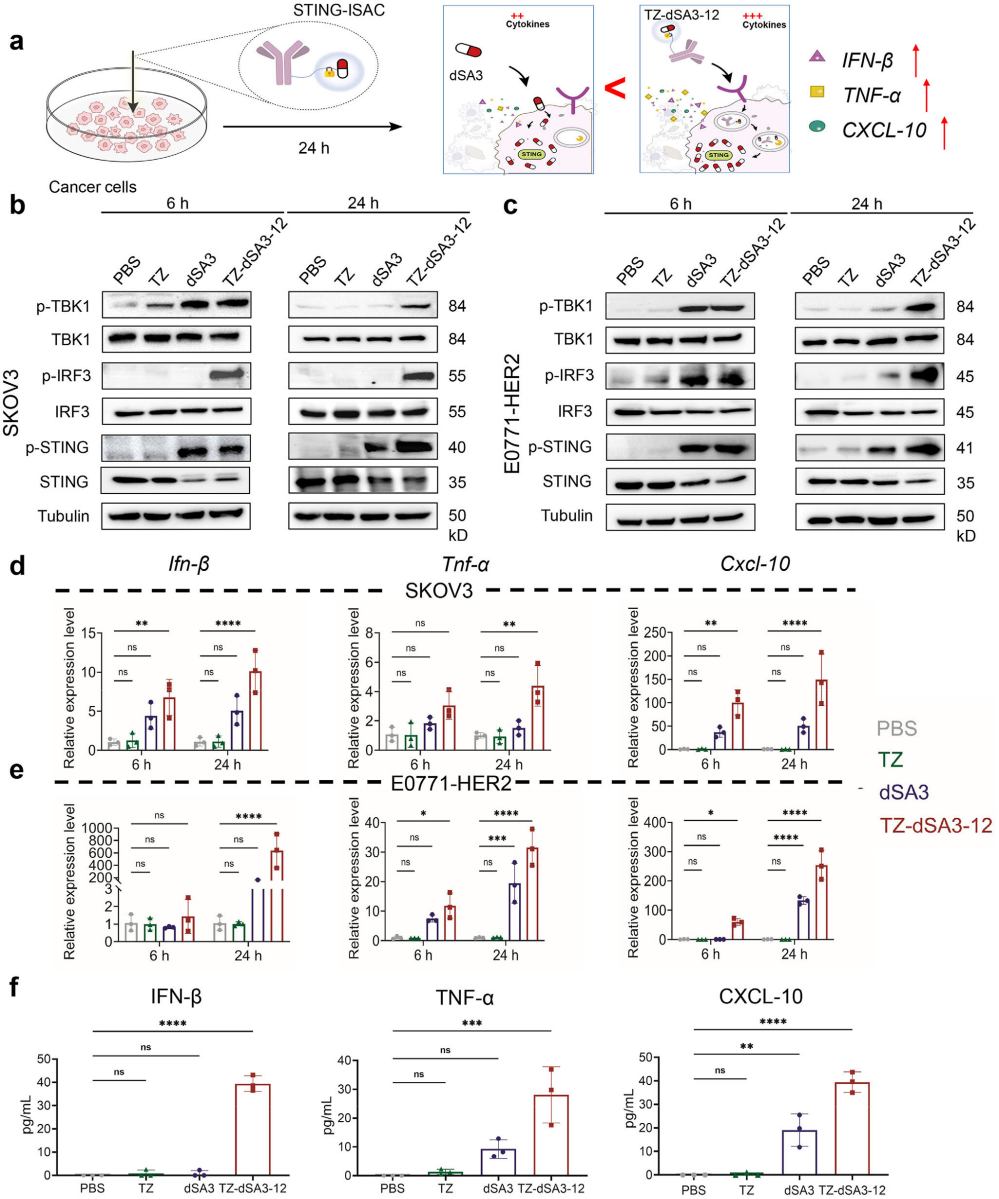
TZ‐dSA3‐12 potently stimulates the STING signaling in cancer cells expressing HER2. a) Diagrams of TZ‐dSA3‐12 internalization and stimulation of STING signaling in cancer cells. b, c) Western blot analysis of the levels of phospho‐TBK1 (p‐TBK1), phospho‐IRF3 (p‐IRF3), and phospho‐STING (p‐STING) in SKOV3 cells (b) and E0771‐HER2 cells (c) treated with PBS, dSA3 (100 nM), TZ (100 nM), and TZ‐dSA3‐12 (100 nM) for 6 or 24 h. d, e) Real‐time PCR examination of the gene expressions of *Ifn‐β, Tnf‐α*, and *Cxcl‐10* in SKOV3 (d) and E0771‐HER2 cells (e) treated with PBS, dSA3 (100 nM), TZ (100 nM), and TZ‐dSA3‐12 (100 nM) for 6 or 24 h. f) Enzyme‐linked immunosorbent assay (ELISA) determining the levels of IFN‐β, TNF‐α and CXCL‐10 in the supernatant of SKOV3 cell treated with dSA3 (100 nM), TZ (100 nM), and TZ‐dSA3‐12 (100 nM) for 24 h. For d–f) data were represented three independent experiments as mean ± SD (n = 3); data were analyzed by Ordinary one‐ way ANOVA (Multiple comparisons), ^*^ *p*<0.05, ^**^ *p*<0.01, ^***^ *p*<0.001, ^****^ *p*<0.0001, ns, not significant.

Given that STING and HER2 are constitutively expressed at variable levels in multiple tumor cell types,^[^ ^22^, ^23^ ^]^ we hypothesized that TZ‐dSA3‐12 could effectively activate STING signaling and enhance tumor cell‐intrinsic innate immunity for tumor control (**Figure** **4**a). Initially, we examined the basal expression levels of STING and HER2 in various tumor cell lines. Western blot analysis revealed that SIING and HER2 were highly expressed in both human SKOV3 and SKBR3 cells, while HER2 was negatively expressed in HCT116 and E0771 cells (Figure S3a, Supporting Information). Additionally, we generated murine E0771 cells overexpressing HER2 (E0771‐HER2) to investigate STING activation by TZ‐dSA3‐12 in murine tumor cells (Figure S3a, b, Supporting Information). To evaluate the capacity of TZ‐dSA3‐12 to activate STING signaling in cancer cells, we treated these cells with vehicle (PBS), dSA3, TZ, or TZ‐dSA3‐12 for either 6 or 24 h. The results indicated that treatment with dSA3 or TZ‐dSA3‐12, as opposed to PBS or TZ, resulted in significant increases in the phosphorylation of TBKI, IRF3, and STING in SKOV3 (Figure 4b), E0771‐HER2 (Figure 4c), and SKBR3 cells (Figure S3c, Supporting Information). However, TZ‐dSA3‐12 did not induce phosphorylation of TBK1, IRF3, or STING at 6 or 24 h in HCT116 cells (Figure S3d, Supporting Information), confirming its HER2‐dependent activity. Notably, the ability of dSA3 to activate STING signaling was found to be comparable to that of TZ‐dSA3‐12 in cells treated for 6 h; however, after 24 h of treatment, TZ‐dSA3‐12 exhibited a significantly greater capacity to activate STING signaling than dSA3 (Figure 4b,c; Figure S3c, Supporting Information). This finding indicates a sustained and prolonged effect of TZ‐dSA3‐12 on STING activation. The activation of STING signaling by TZ‐dSA3‐12 was further corroborated by quantitative real‐time PCR (qRT‐PCR) analysis of downstream antitumor effectors, including *Tnf‐α*, *Cxcl‐10*, and *Ifn‐β*, in SKOV3 (Figure 4d) and E0771‐HER2 (Figure 4e) cells. Moreover, TZ‐dSA3‐12 treatment remarkably increased the protein levels of downstream cytokines associated with the STING pathway, such as IFN‐β, TNF‐α, and CXCL‐10 (Figure 4f; Figure S3e, Supporting Information). Additionally, TZ‐dSA3‐12 exhibited minimal cytotoxicity in SKOV3 or MCF‐7 cells (Figure S3f, Supporting Information), implicating that the activation of STING signaling by TZ‐dSA3‐12 did not induce tumor cell death. Collectively, these findings demonstrate that TZ‐dSA3‐12 potently and sustainably activates STING signaling in HER2‐expressing cancer cells, outperforming the traditional STING agonist dSA3.

### 2.4 TZ‐dSA3‐12 Robustly Activates STING in Tumor Microenvironment While Minimizing Off‐Target Toxicity

**Figure 5:**
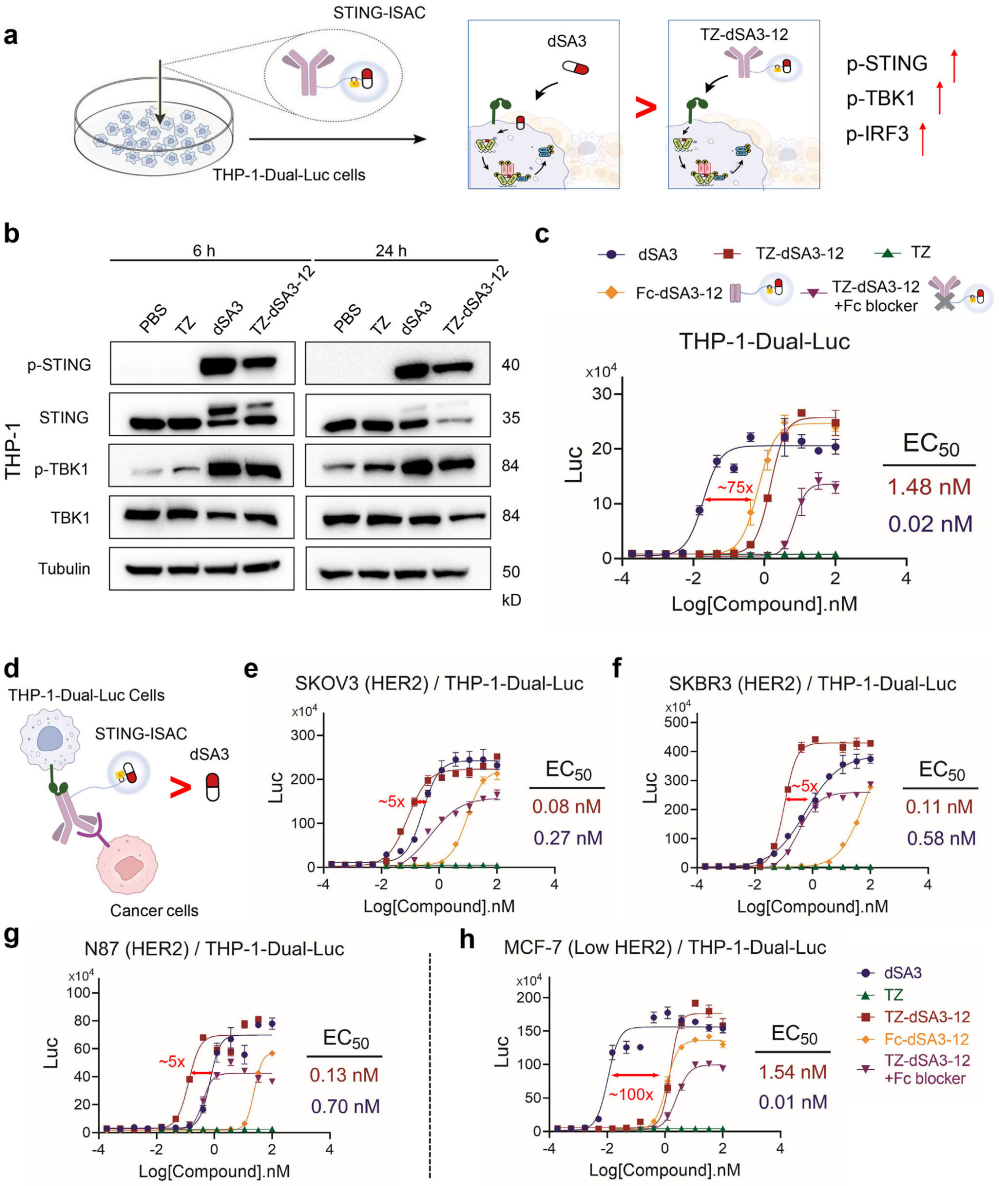
TZ‐dSA3‐12 significantly reduces off‐target toxicity while robustly activates STING in tumor microenvironment. a) The proposed TZ‐dSA3‐12 internalization and stimulation of STING signaling in myeloid cells. b) Western blot analysis of the levels of phospho‐STING (p‐STING) and phospho‐TBK1 (p‐TBK1) in THP‐1 cells treated with PBS, TZ (100 nM), dSA3 (100 nM), and TZ‐dSA3‐12 (100 nM) for 6 or 24 h c) Serial dilutions of dSA3, TZ, TZ‐dSA3‐12, Fc‐dSA3‐12, or TZ‐dSA3‐12+ Fc blocker were incubated with THP‐1‐Dual‐Luc cells for 24 h, and the interferon response was measured by luciferase assay. d) Description Cartoon of the THP‐1‐Dual‐Luc reporter cells and cancer cells (HER2) co‐culture assay. e‐h) Serial dilutions of dSA3, TZ, TZ‐dSA3‐12, Fc‐dSA3‐12, or TZ‐dSA3‐12+ Fc blocker was incubated with THP‐1‐Dual‐Luc cells, followed by co‐culturing with SKOV3 cells (e), SKBR2 cells (f), N87 cells (g), or MCF‐7 cells (h) for 24 h, the interferon response was measured by luciferase assay. For (c, e–h) data were represented three independent experiments as mean ± SD (n = 3). EC_50_ values were derived using the curve fitting function in GraphPad prism 9.0.

We next investigated the STING‐activating effect of TZ‐dSA3‐12 in myeloid cells. To this end, we examined whether TZ‐dSA3‐12 can internalize and translocate to the lysosome in THP‐1 cells (Figure S4a, Supporting Information). Human myeloid THP‐1 cells were treated with PBS, dSA3, TZ, Fc‐dSA3‐12, TZ‐dSA3‐12, or a combination of FcγR blocker (TZ‐dSA3‐12+Fc blocker) for 6 or 24 h in the presence of recombinant human HER2 (**Figure** **5**a). Compared to dSA3, TZ‐dSA3‐12 exhibited reduced efficacy in elevating the phosphorylation levels of TBK1 and STING in THP‐1 cells (Figure 5b). This effect was further diminished in the FcγR‐blocked group (Figure S4b, Supporting Information), suggesting that FcγRI‐mediated endocytosis contributes to TZ‐dSA3‐12 uptake. To quantify STING activation, we assessed the actions of TZ‐dSA3‐12 and its controls in THP‐1‐Dual‐Luc cells. TZ‐dSA3‐12 elicited an IFN response with a half‐maximal effective concentration (EC_50_) of 1.48 nM, which was ≈75 fold less potent than the payload dSA3 (EC_50_ = 0.02 nM, Figure 5c; Figure S4c, Supporting Information), indicating enhanced safety in normal immune tissues.

Next, we evaluated the immunostimulatory activity of TZ‐dSA3‐12 in a THP‐1‐Dual‐Luc and cancer cells co‐culture assay (Figure 5d; Figure S4d, Supporting Information), which mimicking the tumor microenvironment. TZ‐dSA3‐12 elicited robust interferon responses with EC_50_ values of 0.08 nM (SKOV3), 0.11 nM (SKBR3), and 0.13 nM (N87), which were significantly lower than that of the payload dSA3 (Figure 5e–g; Figure S4e, f, Supporting Information). Strikingly, the interferon response in SKOV3/THP‐1‐Dual‐Luc co‐cultured cells (Figure 5e) was ≈50 fold stronger than that in THP‐1‐Dual‐Luc cells alone (Figure 5c), highlighting its synergistic enhancement effect in the tumor microenvironment. Notably, unconjugated HER2 antibodies did not induce any response (Figure 5c,e–h), indicating that STING stimulation depends on dSA3 release. The Fc‐dSA3‐12 conjugate showed **>**100‐fold lower activity than TZ‐dSA3‐12 (Figure S4e, Supporting Information), and FcγR blockade reduced TZ‐dSA3‐12's maximal effect by ≈40% (Figure S4f, Supporting Information). These results suggest that TZ‐dSA3‐12 synergistically activates STING through both Fab‐mediated tumor targeting and FcγR‐mediated myeloid engagement. Furthermore, TZ‐dSA3‐12 induced an over 20 fold stronger interferon response in SKOV3 (high HER2) cells (Figure 5e) than in MCF‐7 (low HER2) cells (Figure 5h), indicating HER2‐dependent activity of TZ‐dSA3‐12. Importantly, TZ‐dSA3‐12‐induced STING activity was ≈100 fold more potent than dSA3 in MCF‐7 cells (Figure 5h), further supporting its reduced off‐target toxicity in normal tissues due to the quaternary ammonium salt‐linker design. In summary, TZ‐dSA3‐12 combines strong stability, potent payload delivery, robust STING activation in the tumor microenvironment, and minimal off‐target effects, making it a promising therapeutic candidate.

### 2.5 TZ‐dSA3‐12 Enhances the Cytotoxicity of T Cells

**Figure 6:**
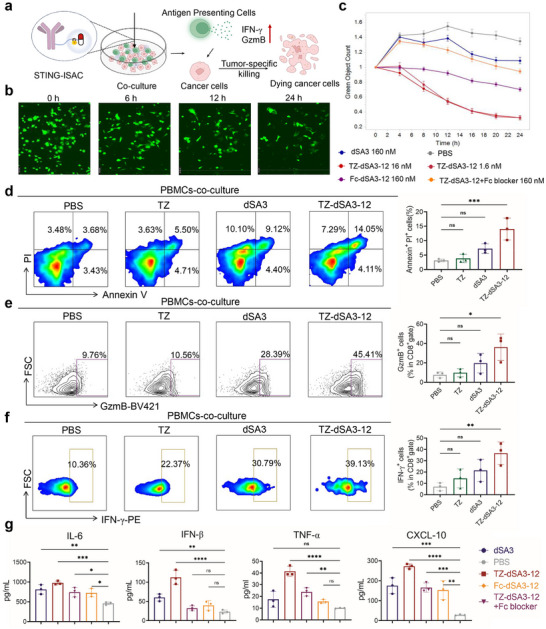
TZ‐dSA3‐12 enhances the cytotoxicity of T cells. a) Schematic of SKOV3 cells‐co‐incubated with activated PBMCs. b, c) Graphs showing count green object confluency as a measure of growth of SKOV3 cells over time in the presence of indicated treatments in PBMCs co‐cultures. The number of green objects in each well was normalized to the number of green objects at its own T = 0 time point. d) Flow cytometry analysis of PBMCs‐mediated killing SKOV3 cells using Annexin V and propidium iodide (PI) double staining. Cells that in the late stage of apoptosis with Annexin V and PI positive were shown. e, f) SKOV3 cells were co‐incubated with activated PBMCs, the levels of GzmB and IFN‐γ in CD8^+^ T cells were examined by flow cytometry. g) Enzyme‐linked immunosorbent assay (ELISA) determining the IL‐6, IFN‐β, TNF‐α and CXCL‐10 levels in the supernatant of SKOV3 cells and PBMCs co‐cultures. For d–g) data were represented three independent experiments as mean ± SD (n = 3); data were analyzed by Ordinary one‐way ANOVA (Multiple comparisons), ^*^ *p*<0.05, ^**^ *p*<0.01, ^***^ *p*<0.001, ^****^ *p*<0.0001, ns, not significant.

To investigate the functional activity of TZ‐dSA3‐12, we conducted co‐culture experiments with peripheral blood mononuclear cells (PBMCs) (**Figure** **6**a). Using the IncuCyte live‐cell imaging system, we first evaluated cell death in co‐cultures of SKOV3‐GFP cells and PBMCs. The results indicated a gradual decrease in the survival of SKOV3‐GFP (green) cells with increasing co‐culture duration (Figure 6b). Notably, TZ‐dSA3‐12 (1.6 nM) exhibited significantly enhanced tumor‐killing activity even at a concentration 100‐fold lower than that of dSA3 (160 nM), Fc‐dSA3‐12 (160 nM), and TZ‐dSA3‐12 + Fc blocker (160 nM). This suggests that the immune activation mechanism of TZ‐dSA3‐12 relies on the synergistic activation of both Fab and Fc regions, surpassing the effects of small molecules (Figure 6c). Consistent with these findings, flow cytometry assays further confirmed that TZ‐dSA3‐12 enhanced the sensitivity of tumor cells to PBMC‐mediated killing. Treatment with TZ‐dSA3‐12 markedly promoted PBMCs‐mediated apoptosis in SKOV3 (Figure 6d) and SKBR3 cells (Figure S5a, Supporting Information). Since granzyme B (GzmB) and IFN‐γ are key mediates of the apoptotic effects of cytotoxic T lymphocytes, we measured their secretion levels. Increased levels of GzmB and IFN‐γ released from CD8^+^ T cells were observed in PBMCs co‐cultured with TZ‐dSA3‐12‐treated cells compared to those treated with PBS (Figure 6e,f; Figure S5b,c, Supporting Information). Furthermore, TZ‐dSA3‐12 treatment markedly elevated the protein levels of IL‐6, IFN‐β, TNF‐α, and CXCL‐10 in PBMC co‐cultures, whereas the Fc‐dSA3‐12 and TZ‐dSA3‐12+Fc blocker treatments exhibited reduced potency (Figure 6g), indicating that TZ‐dSA3‐12 functionally activates the STING pathway. Given the critical role of dendritic cells (DCs) in antitumor immunity, we determined their activation status following co‐cultured with TZ‐dSA3‐12‐treated cancer cells (Figure S5d, Supporting Information). TZ‐dSA3‐12‐treated E0771‐HER2 cancer cells markedly increased the surface expression of activation markers, including CD40, CD80, CD86, MHC‐I, and MHC‐II on bone marrow‐derived dendritic cells (BMDCs) (Figure S5e, Supporting Information), suggesting that TZ‐dSA3‐12‐treated tumor cells enhance the antigen presentation capacity of DCs. Altogether, these results suggest that TZ‐dSA3‐12 enhances the cytotoxicity of PBMCs toward cancer cells through the activation of the STING pathway.

### 2.6 TZ‐dSA3‐12 Inhibits Tumor Growth via Innate Immunity in a Xenograft Mouse Model

**Figure 7:**
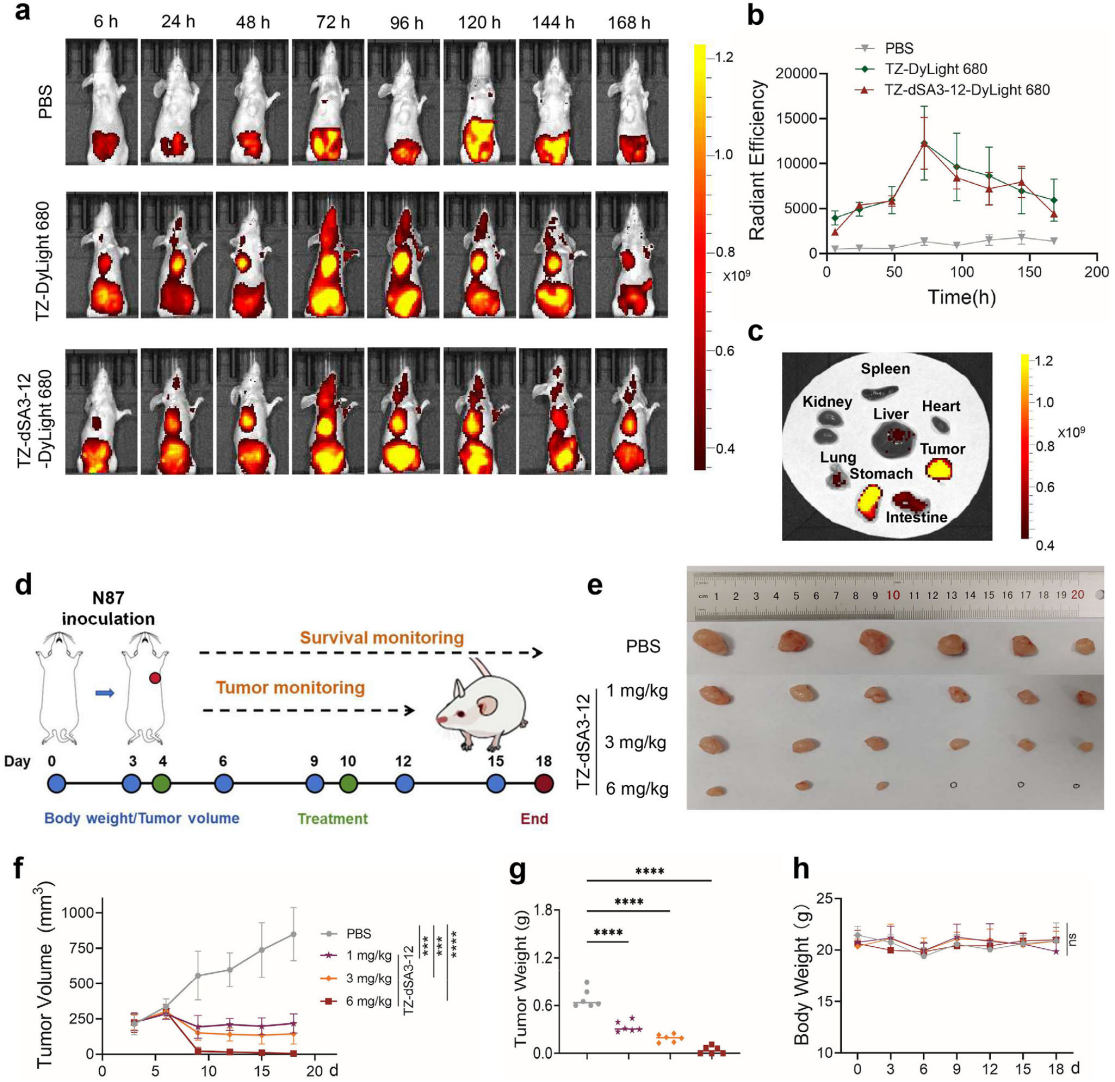
TZ‐dSA3‐12 inhibits tumor growth via innate immune responses in vivo. a) In vivo imaging of mice after administration of TZ‐Dylight680, TZ‐dSA3‐12‐Dylight680 at the indicated time points. b) Quantitative analysis of the fluorescence signals of TZ‐Dylight 680 and TZ‐dSA3‐12‐DyLight 680 in tumors. c) Fluorescence imaging of healthy tissues and the tumor at 24 h after the TZ‐dSA3‐12 administration. d) Groups of CB.17 SCID mice (n = 6) with established N87 tumor were intravenously (*i.v*.) injected with PBS, TZ‐dSA3‐12 (1, 3 and 6 mg/kg) at the indicated times. e) Tumors collected post injection of each TZ‐dSA3‐12 in the N87 tumor model. f, g) Volumes (f) and weights (g) of the collected tumors from the N87 tumor model. h) The curves of the body weight of TZ‐dSA3‐12 treated mice were recorded every three days. For f‐h) data were analyzed by Ordinary one‐ way ANOVA (Multiple comparisons). ^***^ *p*<0.001, ^****^ *p*<0.0001, ns, not significant.

To investigate the innate immune responses elicited by TZ‐dSA3‐12 in vivo, we first examined its biodistribution in N87 tumor‐bearing BALB/c nude mice using whole‐animal imaging. After administration of DyLight 680‐labeled TZ and TZ‐dSA3‐12 to mice via the tail vein at a dose of 10 mg kg^−1^, an intense fluorescence signal at the tumor site was clearly visible within 6 h, and persisting for ≈7 days (**Figure** **7**a,b). Moreover, the fluorescence signal intensity curve of TZ‐dSA3‐12‐DyLight 680 was similar to that of TZ‐DyLight 680 (Figure 7a,b). In addition, the fluorescence intensity in tumor tissue was considerably higher than that in healthy tissues (Figure 7c). These data suggest that TZ‐dSA3‐12 has excellent tumor‐targeting ability and prolonged half‐life in vivo. Next, we assessed the antitumor efficacy of TZ‐dSA3‐12 in CB.17 SCID mice subcutaneously implanted with N87 gastric cancer cells. Previous studies have shown that STING ISAC can suppress tumors via innate immunity.^[^ ^19^ ^]^ When tumors reached ≈200 mm^3^, mice received a weekly tail vein injection of PBS or TZ‐dSA3‐12 (1, 3, and 6 mg kg^−1^) (Figure 7d). Compared to the PBS control group, the TZ‐dSA3‐12 (1 and 3mg kg^−1^) treatment group displayed significant and sustained suppression of tumor growth with an inhibition rate of 87.71% and 89.75% (Figure 7e,f). Moreover, 6 mg/kg of TZ‐dSA3‐12 treatment nearly completely eradicated xenograft tumor growth (Figure 7g), further confirming the satisfactory antitumor efficacy of TZ‐dSA3‐12 in vivo. All treatment groups did not remarkably alter the mice's body weight (Figure 7h). Together, these data suggest that TZ‐dSA3‐12 activates the innate immunity to inhibit tumor growth.

### 2.7 TZ‐dSA3‐12 Enhances Antitumor Immunity in a Syngeneic Mouse Model

**Figure 8:**
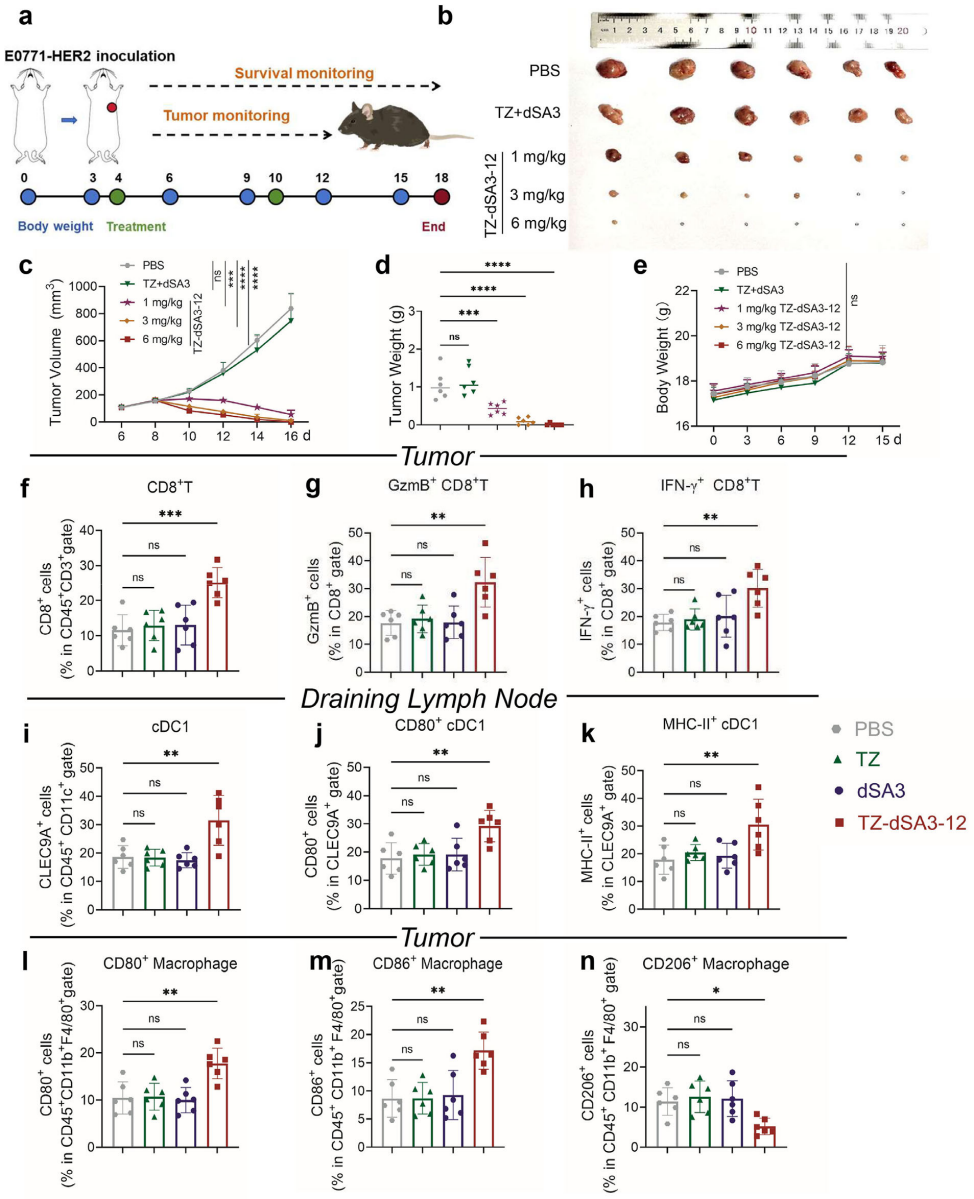
TZ‐dSA3‐12 elicits robust antitumor immunity in E0771‐HER2 tumor xenograft model. a) Groups of C57BL/6 mice (n = 6) with established E0771 tumors expressing human HER2 were intravenously (*i.v*.) injected with PBS, TZ + dSA3, or TZ‐dSA3‐12 (1, 3 and 6 mg/kg) at the indicated times. b) Tumors collected post injection of each TZ‐dSA3‐12 in the E0771‐HER2 tumor model. c, d) Volumes (c) and weights (d) of the collected tumors from the E0771‐HER2 tumor model. e) The curves of the body weight of TZ‐dSA3‐12 treated mice were recorded every three days. f–n) Flow cytometry analyzing the numbers of tumor‐infiltrating CD8^+^ T cells (f), and effector molecules GzmB (g) and IFN‐γ (h) in CD8^+^ T cells, the numbers of draining Lymph Node‐infiltrating cDC1 (i), and surface expression levels of CD80 (j), MHC‐II (k) in cDC1 and CD80 (l), CD86 (m), and CD206(n) on tumor macrophages cells. For (c–n) data were analyzed by Ordinary one‐way ANOVA (Multiple comparisons), ^*^ *p*<0.05, ^**^ *p*<0.01, ^***^ *p*<0.001, ^****^ *p*<0.0001, ns, not significant.

To evaluate the anti‐tumor effect of TZ‐dSA3‐12 under immunocompetent conditions, we utilized a mouse tumor model in which C57BL/6J mice were subcutaneously implanted with E0771 cells that stably express human HER2 (E0771‐HER2). When tumors reached ≈80 mm^3^, mice received weekly tail vein injections of either a TZ (6 mg kg^−1^) + dSA3 (0.7 mg kg^−1^) mixture or TZ‐dSA3‐12 (1, 3, and 6 mg kg^−1^) for two consecutive weeks (**Figure** **8**a). At a low dose of 1 mg kg^−1^, TZ‐dSA3‐12 exhibited significant and sustained suppression of tumor growth compared to the TZ + dSA3 group (Figure 8b), indicating superior efficacy of TZ‐dSA3‐12 at an equimolar dosage. Notably, treatment with 6 mg/kg of TZ‐dSA3‐12 nearly abolished xenograft tumor growth (Figure 8c,d), further confirming its potent in vivo antitumor activity. Both TZ‐dSA3‐12 and the control treatments were well tolerated by the mice, with no adverse effects on body weight (Figure 8e). Furthermore, analysis of the tumor‐infiltrating lymphocyte (TILs) profile revealed that TZ‐dSA3‐12 increased the proportion of tumor‐infiltrating CD8^+^ T cells compared to monotherapy (Figure 8f). Consistently, TZ‐dSA3‐12 enhanced the production of effector molecules, including GzmB and IFN‐γ, by tumor‐infiltrating CD8^+^ T cells compared to monotherapy (Figure 8g,h), reinforcing its role in augmenting cytotoxic CD8^+^ T cells activity. Additionally, the cDC1 population in draining lymph nodes (dLN) showed upregulated expression of CD80, MHC‐II, CD40, CD86, and MHC‐I following TZ‐dSA3‐12 treatment (Figure 8i–k; Figure S6a–c, Supporting Information), suggesting TZ‐dSA3‐12 potentiates the antitumor efficacy by reprogramming the immune microenvironment. Interestingly, TZ‐dSA3‐12 reduced the total DCs population in tumors (Figure S6d, Supporting Information) while increasing it in dLN (Figure 8i), implying DCs migration from tumors to dLN for T cell activation. This was supported by elevated expression of co‐stimulatory molecules (CD40, CD80, CD86, MHC‐I, MHC‐II) in dLN but not in tumors (Figure 8j,k; Figure S6e–i, Supporting Information). To investigate the role of the STING pathway in TZ‐dSA3‐12‐mediated antitumor effects, we administered H151, a selective STING inhibitor,^[^ ^24^ ^]^ to E0771‐HER2‐bearing C57BL/6 mic**e** alongside TZ‐dSA3‐12 (3 mg kg^−1^) for two weeks (Figure S7a, Supporting Information). The combination of H151 and TZ‐dSA3‐12 significantly attenuated the tumor‐suppressive effects of TZ‐dSA3‐12, as evidenced by accelerated tumor growth and increased tumor weight (Figure S7b–d, Supporting Information), without affecting body weight (Figure S7e, Supporting Information). Flow cytometry analysis revealed that combination treatment with H151 and TZ‐dSA3‐12 reduced tumor‐infiltrating CD8+ T cells (Figure S7f, Supporting Information) and diminished GzmB and IFN‐γ production compared to TZ‐dSA3‐12 treatment group (Figure S7g, h, Supporting Information). Furthermore, H151 decreased the total DCs population in dLN following TZ‐dSA3‐12 treatment (Figure S7i, Supporting Information) and abrogated TZ‐dSA3‐12‐induced upregulations of CD40, CD80, and MHC‐II (Figure S7j–l, Supporting Information), confirming the dependency of TZ‐dSA3‐12 on STING signaling. Given the prevalent of tumor‐associated macrophages (TAMs) in the tumor microenvironment, where M1‐polarized TAMs promote tumor cell death while M2‐polarized TAMs suppress the antitumor immune response and promote tumor growth, we assessed the impact of TZ‐dSA3‐12 on macrophages polarization. Our findings revealed TZ‐dSA3‐12 treatment increased the M1‐like macrophages (MHC‐II^+^CD206^‐^) (Figure 8l–n; Figure S6j, Supporting Information) while decreasing M2‐like macrophages (MHC‐II^‐^CD206^+^) (Figure 8n). These results demonstrate that the TZ‐dSA3‐12 induces a shift from M2 to M1 polarization of macrophages within the tumor microenvironment. Taken together, these findings suggest that TZ‐dSA3‐12 exerts its antitumor effects by promoting DCs maturation, enhancing T cell cytotoxicity, and inducing M1 macrophage polarization.

### 2.8 TZ‐dSA3‐12 Induces a Long‐Term Immune Memory Effect

**Figure 9:**
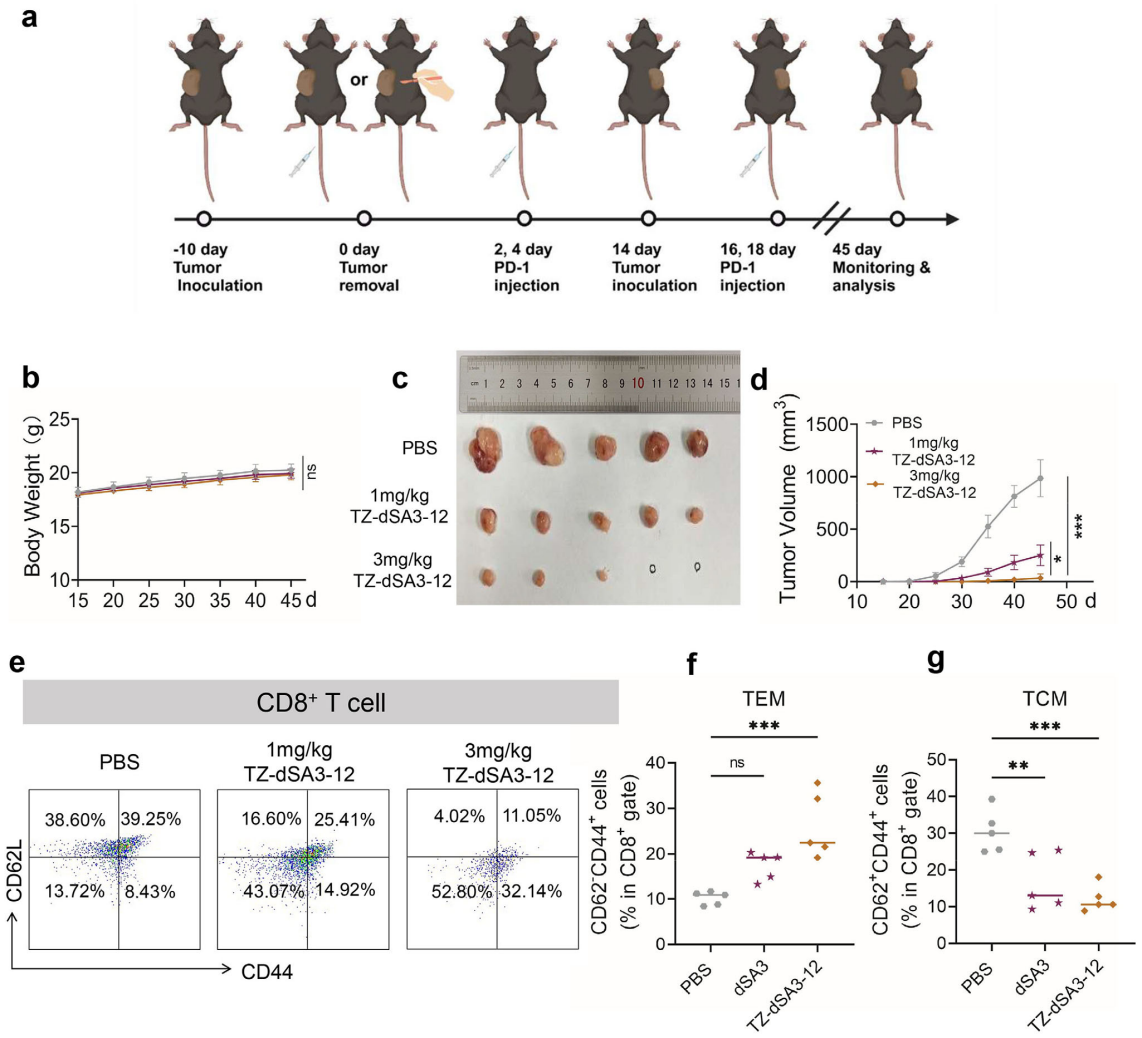
TZ‐dSA3‐12 induces a long‐term immune memory effect. a) Schematic representation of the experimental schedule for evaluating the long‐term immuno‐logical memory response induced by TZ‐dSA3‐12 in animals. b) Body weight changes of tumor‐bearing mice during the tumor rechallenge. c) Tumors collected post injection of each TZ‐dSA3‐12 in the E0771‐HER2 tumor model. d) Tumor‐growth curves of surgery and TZ‐dSA3‐12‐treated mice (n = 5). e) Flow cytometry measuring the depicting subsets of memory T cells (CD3^+^CD8^+^) in spleens (n = 5). f, g) Flow cytometry analysis of the populations of central memory T cells (f) and effector memory T cells (g) within CD8^+^ T cells. Data were analyzed by Ordinary one‐way ANOVA (Multiple comparisons), ^*^ *p*<0.05, ^**^ *p*<0.01, ^***^ *p*<0.001, ns, not significant.

To assess the long‐term immune memory effects of TZ‐dSA3‐12‐mediated therapy, tumor‐bearing C57BL/6J mice were rechallenged with E0771‐HER2 cells. As illustrated in **Figure** **9**a, tumors were initially implanted and subsequently subjected to either surgical excision or TZ‐dSA3‐12 treatment. Following a 14‐day interval, E0771‐HER2 cells were reintroduced to evaluate tumor regrowth. Throughout the treatment period, no significant changes in body weight were observed in either experimental group (Figure 9b). Analysis of tumor growth following rechallenge (Figure 9c,d) indicated that TZ‐dSA3‐12‐treated mice exhibited a robust immune‐memory effect compared to the surgical group. To elucidate the mechanisms by which TZ‐dSA3‐12 prevents tumor recurrence, we examined the populations of memory T cells in splenocytes, specifically effector memory T cells (Tem, CD3^+^CD8^+^CD44^+^CD62L^−^) and central memory T cells (Tcm, CD3^+^CD8^+^CD44^+^CD62L^+^). Flow cytometry analysis revealed a significantly increased proportion of Tem and a decreased proportion of Tcm among CD8^+^ T lymphocytes in TZ‐dSA3‐12‐treated mice (Figure 9e–g), confirming the long‐term immune memory effect mediated by TZ‐dSA3‐12. Given that Tcm cells can readily differentiate into effector cells upon antigen re‐exposure, these findings may explain the observed increase in Tem and the corresponding decrease in Tcm.

### 2.9 In Vivo Safety Assessment of TZ‐dSA3‐12

Finally, we evaluated the in vivo safety profile of TZ‐dSA3‐12. C57BL/6J mice received a single intraperitoneal injection of 3 mg kg^−1^ TZ‐dSA3‐12, with plasma samples collected at designated time points. dSA3 served as a control in this experiment. Plasma levels of cytokines, including TNF‐α, IFN‐β, IL‐6, and CXCL‐10, were quantified as safety biomarkers. The results demonstrated that administration of dSA3 induced markedly elevated levels of systemic cytokines, with TNF‐α peaking at 3 h, IFN‐β at 12 h, and both IL‐6 and CXCL‐10 reaching maximal levels at 24 h post‐injection, followed by gradual declines (Figure S8a, Supporting Information). In contrast, TZ‐dSA3‐12‐treated mice exhibited significantly attenuated cytokine induction compared to the dSA3 group (Figure S8a, Supporting Information). For instance, TNF‐α levels in TZ‐dSA3‐12‐treated mice were ≈10 fold lower than those in dSA3‐treated mice. Furthermore, intrathecal administration of TZ‐dSA3‐12 showed no significant alterations in blood biochemical parameters, including alanine aminotransferase (ALT), aspartate aminotransferase (AST), α‐hydroxybutyrate dehydrogenase (HBDH), and creatinine (CREA) (Figure S8b, Supporting Information), or organ‐to‐body weight ratios (heart, liver, spleen, kidney, lung; Figure S8c, Supporting Information). Additionally, histological analysis by a blinded pathologist further confirmed no abnormalities in major organs (Figure S8d, Supporting Information). Furthermore, to evaluate the potential long‐term toxicity caused by TZ‐dSA3‐12, C57BL/6J mice were administrated were intravenously administrated TZ‐dSA3‐12 (3 mg/kg) or dSA3 (1.5 mg kg^−1^) once every 5 days for 6 consecutive times (Figure S9a, Supporting Information). The results demonstrated that TZ‐dSA3‐12 induced lower systemic IFN‐β and IL‐6 levels than dSA3 (Figure S9b, Supporting Information), with no adverse effects on body weight (Figure S9c, Supporting Information), or organ histology (Figure S9d, Supporting Information). Collectively, these findings demonstrate that TZ‐dSA3‐12 exhibits a favorable safety profile with minimal systemic cytokine release, no detectable organ toxicity, and negligible long‐term adverse effects in vivo.

## 3 Discussion

STING represents a promising target for cancer immunotherapy. However, the clinical application of single STING agonists (e.g., ADU‐S100, MK‐1454) has been limited by poor pharmacokinetics, systemic toxicity, and inefficient cytosolic delivery. To overcome these challenges, the pharmaceutical industry has begun to explore developing ISACs to target the delivery of STING agonists into tumor cells, thereby minimizing off‐target toxicity. In this study, we developed a novel class of quaternary ammonium salt‐linked STING agonist‐ISACs, featuring a stable linker and a unique dual synergistic activation mechanism, achieving both toxicity reduction and efficacy enhancement of the STING agonist.

STING agonist‐ISACs represent a paradigm shift in cancer immunotherapy, offering distinct advantages over traditional ADCs. Conventional ADCs rely on cytotoxic payloads that kill both tumor and healthy cells through the Fab‐mediated targeting of antibodies, leading to dose‐limiting toxicities and a narrow therapeutic window. In contrast, STING agonist‐ISACs leverage both Fab and Fc regions of the antibody to achieve robust STING activation specifically within the tumor microenvironment. Meanwhile, in normal immune tissues—where Fab binding to tumor antigens is absent—STING activation is limited to Fc‐mediated effects, thus reducing systemic toxicity. However, previously developed STING agonist‐ISACs have failed to fully realize this potential. For instance, the instability of the carboxylic ester‐based linker in XMT‐2056 led to the inevitable off‐target toxicity in clinic trials.^[^ ^20^ ^]^ Similarly, the limited tumor penetration of CDN STING agonist IMSA172 resulted in the limited antitumor efficacy of αEGFR‐172 ADC^[^ ^18^ ^]^ in vivo.

In this study, we addressed these limitations by developing a novel quaternary ammonium‐based linker and selecting the non‐CDN STING agonist dSA3 as the payload. Although dSA3 monotherapy clinical trials for hematological malignancies were discontinued due to off‐target toxicity,^[^ ^25^ ^]^ it remains an attractive candidate for ISACs development due to its high stability, potent activity, and short half‐life.^[^ ^13^ ^]^ The primary challenge for dSA3 remains the absence of suitable conjugation sites. The key innovation of our design lies in the synthesis of the quaternary ammonium salt conjugation site, which simultaneously resolves challenges related to rigidity, conjugation chemistry, and solubility. Structure‐activity relationship (SAR) studies have demonstrated that even minor modifications to diABZI‐based STING agonists (e.g., morpholine substitution to form a quaternary ammonium salt) can significantly enhance solubility without requiring complex polysaccharide modifications.^[^ ^21^ ^]^ Furthermore, this modification renders dSA3 prodrug‐like, masking its pharmacological activity until tumor‐localized lysosomal cleavage (e.g., by cathepsin B) releases the payload, minimizing off‐target effects and broadening the therapeutic window.

Subsequent investigations also verified the efficacy of our design. The tumor cells and THP‐1 cells co‐culture assay demonstrated that TZ‐dSA3‐12 robustly activates the STING pathway in the tumor microenvironment through HER2‐dependent targeting and FcγR‐mediated innate immune activation. FcγR interactions promote DC uptake and cross‐presentation, synergizing with STING‐induced IFN‐I to enhance T‐cell priming. Notably, TZ‐dSA3‐12 exhibited several‐fold higher activity than free dSA3 in this context.  In contrast, in THP‐1 cells alone (which lack HER2 expression), TZ‐dSA3‐12 showed almost 75 fold lower activity than dSA3, indicating improved safety in normal immune tissues. Furthermore, the potent antitumor effect observed in the N87 xenograft model corroborated the strong innate immune activation mediated by TZ‐dSA3‐12.

In addition to innate immunity, we further investigate the effect of TZ‐dSA3‐12 on adaptive immunity in immunocompetent mice. STING‐activation‐mediated type‐I interferon (IFN) production is critical for initiating adaptive immune responses and represents a promising antitumor strategy.^[^ ^26^ ^]^ Our results showed that activation of the STING pathway by TZ‐dSA3‐12 facilitated the release of type I IFNs, thereby bridging innate and adaptive immune responses. Furthermore, our in vitro and in vivo results demonstrated that TZ‐dSA3‐12 strongly induced the surface expression of CD80 and CD86 on DCs within the tumor microenvironment, and boosted the production of IFN‐γ and GzmB by tumor‐infiltrating CD8^+^ T cells. These findings suggest that TZ‐dSA3‐12 modulates DC‐CD8^+^T cell immunity for tumor control, aligning with previous reports that EGFR‐targeted STING agonist ADC promotes DCs maturation, antigen cross‐presentation, and CD8^+^ T cells expansion.^[^ ^18^ ^]^ Moreover, we observed that the DCs population decreased in the tumors and increased in the dLN following TZ‐dSA3‐12 treatment, suggesting that TZ‐dSA3‐12‐activated DCs likely capture tumor antigens and migrate to dLN, where they prime T cells^[^ ^27^ ^]^ and induce a long‐term immune memory response.

Macrophages are the most abundant immune cells in tumors, with M1 macrophages inhibiting tumor growth by secreting proinflammatory factors and engaging in phagocytosis, whereas M2 macrophages promote immunosuppression and tumor progression.^[^ ^28^, ^29^ ^]^ Furthermore, we showed that TZ‐dSA3‐12 abrogates the immunosuppressive function of macrophages by resetting them toward the M1 phenotype, thereby enhancing their phagocytic activity and contributing to the anti‐tumor effects. Collectively, we provide clear evidence that TZ‐dSA3‐12 can suppress tumor growth by coordinating innate and adaptive immune responses.

In conclusion, the novel quaternary ammonium linker‐based ISAC design presents promising therapeutic potential and validates the unique synergistic STING activation mechanism of ISAC. This approach also addresses previous druggability challenges associated with the clinical STING agonist dSA3. The ISAC strategy in this work could be expanded to other indications, such as chronic inflammatory diseases by delivering immune agonists to the inflammatory lesion area to minimize the systemic immune response. For instance, ISACs could be used to enhance the immunogenicity of vaccines in vaccine delivery, especially in cases where a potent T‐cell response is required, such as viral diseases. Additionally, the potential of combination therapy remains to be studied, such as ISAC combined with immune checkpoint inhibitors (PD‐1/PD‐L1 inhibitors), or cell therapies (such as CAR‐T) and other ADCs.

## 4 Experimental Section

### Cell Lines, Reagents, Antibodies, and Analytical methods

The human ovarian carcinoma cell line SKOV3, breast carcinoma cell lines SKBR3 and MCF‐7, gastric cancer cell line N87, and mouse breast carcinoma cell line E0771 were obtained from the Institute of Basic Medicine, Chinese Academy of Medical Sciences (Beijing, China). Human STING THP‐1‐Dual‐Luc reporter cells (luciferase reporter gene under the control of NF‐κB and IRF3 promoters) were kindly provided by Junhai Xiao (National Engineering Research Center for the Emergency Drug, Beijing Institute of Pharmacology and Toxicology, China). E0771‐HER2 cell lines were generated by stable transfection of E0771 cells with the plasmid pCMV3‐ERBB2‐Myc (HG10004‐CM). SKOV3, SKBR3, MCF‐7, N87, E0771‐HER2, and E0771 cells were cultured in Dulbecco's Modified Eagle Medium (DMEM; Gibco, Pittsburgh, PA, USA), while THP‐1‐Dual‐Luc cells were maintained in RPMI 1640 medium. All culture media were supplemented with 10% fetal bovine serum (FBS; Hyclone, Logan, UT, USA) and 100 U mL^−1^ penicillin‐streptomycin. STING agonist was purchased from MedChemExpress (MCE). Trastuzumab and human IgG1k were obtained from Sino Biological Inc. Unless specified otherwise, all other reagents and solvents were commercially available and used without further purification. The reaction process was monitored by thin‐layer chromatography (TLC). High‐performance liquid chromatography (HPLC) analysis was performed using an Agilent 1260 Series Liquid Chromatography (LC) system (California, CA, USA). Nuclear magnetic resonance (NMR) spectra were recorded on a JNM‐ECA‐400 spectrometer. Additional reagents and commercial assay kits used in this study are listed in Table S1 (Supporting Information). The antibodies employed are detailed in Tables S2 and S3 (Supporting Information).

### Isolation and Culture of Bone Marrow‐Derived Dendritic Cells

Bone marrow (BM) cells were isolated from the femurs and tibiae of 6‐ to 8‐week‐old female C57BL/6J mice by flushing with RPMI 1640 medium containing 10% FBS. The collected cells were then filtered through a 70 µm filter, centrifuged, and treated with red blood cell (RBC) lysis buffer (150 mM NH_4_Cl, 10 mM NaHCO_3_, 1 mM EDTA) for 3 min to remove erythrocytes. The remaining BM cells were cultured in RPMI 1640 medium supplemented with 10% FBS, 0.1% streptomycin, 100 U mL^−1^ penicillin, 55 µM β‐mercaptoethanol, 50 ng mL^−1^ mGM‐SF (Peprotech), and 20 ng mL^−1^ mIL‐4 (Peprotech). The medium was refreshed every 3 days. After 7 days of culture, ≈90% of the BM cells differentiated into dendritic cells (DCs).

### Animal Treatment

Female C57BL/6J mice (6–8 weeks old) were purchased from GemPharmatech Co., Ltd. (Nanjing, Jiangsu, China). The mice were housed under specific pathogen‐free (SPF) conditions, and all experimental procedures were conducted in accordance with the guidelines approved by the Animal Ethics Committee of the Institute of Medicinal Biotechnology, Chinese Academy of Medical Sciences (approval ethical numbers IMB‐20240912D801 and IMB‐20241018D801). For in vivo experiments, E0771‐HER2 cells (2 × 10⁶ cells) were subcutaneously injected into the right abdomen of each mouse. After 4 days, mice were intravenously (i.v.) injected with PBS, TZ, dSA3, or TZ‐dSA3‐12 (1, 3, and 6 mg kg^−1^) once a week. Tumor growth and body weight were monitored every two and three day**s**, respectively. Tumor volume was calculated as π/6 × tumor length × (tumor width) ^2^. At the experimental endpoint, mice were euthanized, and tumors were excised for flow cytometric analysis of tumor‐infiltrating immune cells. For the immunological memory study, E0771‐HER2 tumors were implanted on the right flank and treated either with surgical resection or TZ‐dSA3‐12 pharmacodynamics when the tumor size reached 50–60 mm^3^. In the surgical group, primary tumors were completely excised, and the incision was sutured.  Fourteen days later, the immune memory effect was detected by injecting 2 × 10⁶ E0771‐HER2 cells intravenously into the left side of the mice. Spleens from both groups of mice were collected, prepared as single‐cell suspensions, and then stained with CD45, CD3, CD4, CD8, CD62L, and CD44 antibodies for flow cytometry assay.

### Isolation and Flow Cytometric Analysis of Tumor‐Infiltrating T Lymphocytes

To prepare single‐cell suspensions, tumor tissues were collected and cut into small pieces and then filtered through a 70 µm cell strainer. T cells were analyzed using staining with CD45, CD3, CD8, IFN‐γ, and GzmB. For dendritic cell analysis, cells were labeled with CD45, CD11c, CD103, MHC‐II, CD80, CD86, and CD40. For Treg analysis, cells were marked with CD45, CD3, CD4, CD25, and Foxp3. To analyze MDSCs, cells were stained with CD45, CD11b, MHC‐II, F4/80, Ly6C (M‐MDSC), and Ly6G (PMN‐MDSC). For macrophage analysis, cells were labeled with CD45, CD11b, F4/80, MHC‐II (M1), and CD206 (M2). Stained cells were quantified by flow cytometry using guavaSoft 3.1 software.^[^ ^30^ ^]^

### Real‐Time Fluorescence Quantitative PCR

Total RNA was extracted from tissues and cells using TRIzol reagent (Thermo Fisher Scientific) according to the manufacturer's instructions. Reverse transcription and qPCR were performed using the UniPeak U+ One Step RT‐qPCR SYBR Green Kit (Vazyme Biotechnology, R226). Relative mRNA expression levels were determined using the ΔΔCt method, with GAPDH as the reference gene. Primers were designed using the online tool Primer3Plus and are listed in Table S4 (Supporting Information).

### ELISA Assays

The levels of CXCL‐10, IFN‐β, IL‐6, and TNF‐α in the culture medium were quantified by DuoSet ELISA kits from R&D Systems (Cat# DIFNB0, MIFNB0, DIP100, DY466‐05, MTA00B, DTA00D, and D6050B, respectively). The dose‐response curves were generated using the GraphPad Prism software package.

### Cell Counting Kit‐8 Assays

SKOV3 or MCF‐7 cells were seeded into 96‐well plates at a density of 8000 cells/well and treated with various concentrations of TZ‐dSA3‐12 for 24 h (37°C, 5% CO₂). After 10 µL of Cell Counting Kit‐8 (CCK‐8) reagent (C0005, TargetMol, USA) was added to each well, the cells were incubated at 37°C for another 3 h. The absorbance was measured at an optical density (OD) of 450 nm using a microplate reader (Thermo Fisher Scientific, Inc., MA, USA).

### Western Blot Analysis

Cells were cultured in 6‐well plates and treated as specified. Following treatment, the culture medium was aspirated, and wells were washed twice with PBS. Whole‐cell extracts were prepared using cell lysis buffer (20 mM Tris‐HCl pH 7.6, 250 mM NaCl, 3 mM EDTA, 3 mM EGTA, 0.5% NP40) supplemented with protease inhibitors (1 mM PMSF, 1 mM DTT, 1 mM Na₃VO₄, 10 mM PNPP, phosphatase cocktail). Lysates were centrifuged at 12,000 ×  *g* for 15 min at 4°C, and the supernatants were mixed with 5 × loading buffer and boiled for 10 mintues. Proteins were resolved by 10% SDS‐PAGE and transferred onto polyvinylidene difluoride (PVDF) membranes (Millipore, IPVH00010). Membranes were probed with the following primary antibodies: p‐STING (Cell Signaling Technology, S366, S365), STING (Cell Signaling Technology, D2P2F), p‐IRF3 (Cell Signaling Technology, S396), IRF3 (Cell Signaling Technology, D83B9), p‐TBK1 (Cell Signaling Technology, S172), TBK1 (Cell Signaling Technology, D1B4), Tubulin (Cell Signaling Technology, 2146), and HER2 (Cell Signaling Technology, D8F12). HRP‐conjugated anti‐mouse (Cell Signaling Technology, 7076) or anti‐rabbit (Cell Signaling Technology, 7074) secondary antibodies were used for detection. Protein bands were visualized by the Tanon 5200 chemiluminescence imaging system (Tanon, Shanghai, China).^[^ ^31^ ^]^

### THP‐1 Reporter Cell Assays

For luciferase reporter assays, THP‐1‐Dual‐Luc cells were seeded in a 96‐well plate (50,000 cells/well) and treated with the indicated compounds for 24 h at 37 °C under 5% CO₂. For cancer cell and THP‐1‐Dual‐Luc reporter cell co‐culture assays, cancer cells (8000 cells/well) were plated overnight in 96‐well plates. The medium was replaced with assay medium (RPMI‐1640, 10% FBS, 1% penicillin), and the specified detection reagent was added, followed by incubation at 37°C for 20 min. THP‐1‐Dual‐Luc reporter cells (50 000 cells/well) were added and co‐cultured for 24 h. Luciferase activity in supernatants was measured using the ANTI‐Luc luminescence detection reagent (Invivogen, Cat# rep‐qlc) on a SpectraMax M5 plate reader. All dose‐response curves were generated using GraphPad Prism software. The EC₅₀ values were determined by four‐parameter curve fitting in GraphPad Prism.

### Cancer Cell/PBMC Co‐Cultures Killing Assays

Cancer cell cytotoxicity was assessed using the IncuCyte Live‐Cell Analysis System. Cancer cells were plated and incubated overnight, treated with test compounds for 20 min at 37°C, and co‐cultured with PBMCs at a 1:2 (cancer cell: PBMCs) ratio. The plates were then placed in the IncuCyte system (37°C, 5% CO₂) and imaged every 2 h for 24 h. The change in the number of green fluorescent objects over time (cancer cells) was quantified using IncuCyte S3 (version 2022B) software. For the co‐culture assay of killing efficiency, PBMC cells were seeded at a ratio of 2:1 onto the previously plated tumor cells and then co‐cultured for 24 h. Subsequently, the co‐cultured cells were stained with fluorescent antibodies for Annexin V, PI, CD3, CD45, CD8, IFN‐γ, and GzmB. All stained cells were quantified using a Guava easyCyte flow cytometer and guavaSoft 3.1 software.

### Cancer Cell/BMDC Co‐Culture Assays

To assess DC activation and antigen delivery, E0771‐HER2 cells were seeded into 6‐well plates, and the following day, BMDCs were added to each well and treated with TZ‐dSA3‐12 for 24 h. The co‐cultured cells were then harvested and stained with fluorescent antibodies directed against CD40, CD80, CD86, MHC‐I, and MHC‐II. All stained cells were quantified using a Guava easyCyte flow cytometer and guavaSoft 3.1 software.^[^ ^30^ ^]^

### Synthetic Procedures

The method of synthesis of linker drug, TZ‐dSA3‐2, 4, 6, 8, and 12 was described in the supplementary information+.

### Quality Control of TZ‐dSA3—HIC Analysis

The HIC analysis was performed utilizing a TSK‐gel Butyl‐NPR column (2.5 µm, 4.6 mm × 100mm, Tosoh Corporation, Tokyo, Japan) under conditions of linear gradient elution. Mobile phase A consisted of a mixture of 1.5 M ammonium sulfate and 20 mM PBS at pH 8.0, while mobile phase B consisted of a mixture of 20 mM PBS at pH 8.0 and 15% isopropanol. TZ‐dSA3I‐12 were administered at a flow rate of 0.8 mL/min, employing a linear gradient elution from 0% to 100% of mobile phase B spanning from 0 to 18 min. Detection of drug‐loaded species was achieved by monitoring absorbance at 280 nm.^[^ ^32^ ^]^

### Quality Control of TZ‐dSA3—SEC Analysis

The aggregation of TZ‐dSA3‐12 was assessed using SEC with an Agilent 1260 series HPLC equipped with a TSKgel G3000SWXL column (7.8 mm × 300 mm). The separation of TZ‐dSA3‐12 was performed with a mobile phase composed of 40 mM sodium phosphate, and 150 mM sodium chloride, at pH 7.0. TZ‐dSA3‐12 was injected at a flow rate of 1 mL/min, and UV absorbance was measured at 280 nm.^[^ ^32^ ^]^

### Quality Control of TZ‐dSA3*—*MALDI‐TOF‐MS Analysis

Molecular weight analysis was performed on a Bruker Autoflex MALDI‐TOF system. Samples were pre‐separated by SEC on a Shodex Protein KW‐802.5 SEC column (8.0 mm × 300 mm, 5 µm) with PBS (pH 7.4) as the mobile phase (flow rate: 0.5 mL/min). Spectra were processed using FlexAnalysis 3.4 software.

### Quality Control of TZ‐dSA3—Stability Testing

For stability, assays of linker‐drug (VA‐dSA3) in PBS, a 1 mM stock solution of VA‐dSA3 in DMSO (500 µL) was diluted in PBS (4500 µL) to prepare the working solution. The samples were incubated at 37 °C, and aliquots (100 µL) were collected at subsequent time points (0, 12 h, 1, 2, 3, 4, 5, 6, 7, 10, and 14 d). Samples were stored at–80 °C and the release of dSA3 was determined by HPLC. For the stability assays of TZ‐dSA3‐12 in plasma, 4500 µL of human plasma (BioreclamationIVT) was diluted 2 fold and mixed with 500 µL of TZ‐dSA3‐12 (1 mM stock) to formulate the working solution. Portions (50 µL, 250 nM) from the working solution were placed in an incubator at 37 °C and sampled at predetermined time intervals. Each sample was immediately quenched with acetonitrile prior to being stored at –80 °C. Once all aliquots were collected, samples were thawed at room temperature, centrifuged to pellet proteins, and the resulting supernatants were subjected to HPLC analysis.

### Quality Control of TZ‐dSA3—Enzyme Release Assay

To the Cathepsin B buffer (25.6 mg NaAc, 3.7 mg EDTA·2Na, 58.4 mg NaCl, 9.7 mg Cysteine, pH = 5) containing 1% DMSO (v/v) was added Cathepsin B. The mixture was incubated at 37 °C. Aliquots were collected at set time points and quenched with acetonitrile before being frozen at –80 °C. After collecting the final aliquots, all samples were centrifuged to remove protein and analyzed by HPLC.

### Quality Control of TZ‐dSA3—Flow Cytometry for Binding and Internalization Analysis

The HER2‐positive ovarian cancer cell line SKOV3 was used to assess the binding affinity of TZ‐dSA3‐12 for the HER2 antigen. Briefly, 1 × 10⁶ SKOV3 cells per sample were collected in 1.5 mL tubes and initially rinsed with FACS buffer (phosphate‐buffered saline supplemented with 2% fetal bovine serum). The cells were then incubated with either TZ‐dSA3‐12 or TZ at a concentration of 5 µg mL^−1^ in PBS at 4 °C for 30 min. After two additional washes with FACS buffer, the cells were incubated with PE‐conjugated goat anti‐human IgG at 4 °C for another 30 min. Finally, following three final washes with FACS buffer, cell fluorescence was analyzed using a FACS Calibur (BD Biosciences, USA). To compare the internalization efficiency of TZ‐dSA3‐12 versus TZ, SKOV3 cells were incubated with either compound at 4 °C for 1 h. The cells were then divided into control and experimental groups. Following two rinses with chilled FACS buffer, the experimental groups were transferred to 37 °C for further incubation for 8 or 24 h, whereas the control groups remained at 4 °C for the same duration. At each time point, all samples were washed three times with FACS buffer and subsequently incubated with PE‐conjugated goat anti‐human IgG at 4 °C for 30 min. Flow cytometry analysis was conducted to measure the mean fluorescence intensity (MFI) ratio of PE on the cell surface. The internalization rate was calculated using the following formula: internalization percentage (%) = [100–(MFI of experimental groups/MFI of control groups)] × 100%.

### Laser Confocal Microscope

To assess the binding and internalization efficiency of TZ‐dSA3‐12, SKOV3 cells were cultured in a chamber slide and incubated overnight at 37 °C under 5% CO₂ and 95% air. The medium was then removed, and the cells were treated with FITC‐labeled TZ‐dSA3‐12, followed by incubation at either 4 °C (binding assessment) or 37 °C (internalization observation). At each time point, the cells were rinsed with PBS and stained with DAPI for nuclear staining and Lyso‐Tracker Red for lysosomal labeling. After two additional washes with PBS, cells were imaged using a confocal laser scanning microscope (Zeiss LSM 510, Zeiss, Oberkochen, Germany).

### Molecular Docking

Discovery Studio 4.5 and UCSF Chimera 1.7 were used to perform the molecular overlay of dSA3 with the STING protein. The regularized protein structure was used to identify key amino acid residues (Ser241, Leu159, Tyr240, Pro264, Ser162, Val239, Tyr167, and Tyr167) within the predicted binding site (STING binding pocket). Interatomic distances were measured using Discovery Studio 4.5.

### Statistical Analysis

Data were presented as mean ± SD of three independent experiments. Statistical comparisons between two groups were performed using a two‐tailed unpaired Student's *t*‐test, while comparisons among multiple groups were assessed by two‐way ANOVA followed by Bonferroni's post hoc test. Statistical significance was defined as *p* < 0.05. All graphs and statistical analyses were generated using GraphPad Prism 9.0 (GraphPad Software, Inc., La Jolla, CA, USA).

## Conflict of Interest

The authors declare no conflict of interest.

## Author Contributions

Y.L., B.T., F.X., L.L., and Y.Z. contributed equally to this work. H.D., X.Z., J.X., and W.Z. conceived and designed this study. Y.L., B.T., and F.X. conducted most experiments and data analysis. J.D., J.W., C.S., R.L., L.L., L.L., N.Z., and Y.Z. performed parts of the experiments. X.Z., H.D., and D.X. supervised the study and interpreted results. H.D., Y.L., X.Z. and D.X. wrote and revised the manuscript.

## Supporting information



Supporting Information

## Data Availability

The data that support the findings of this study are available from the corresponding author upon reasonable request.
