# Supplementary material for: Novel Quaternary Ammonium Salt‐Linked STING Agonist Antibody‐Drug Conjugate: Synergistic Activation of Tumor Immunity with Mitigated Off‐Target Toxicity
Source: Adv Sci (Weinh). 2025 Jun 10;12(31):e02270. doi: 10.1002/advs.202502270 (PMC12376676; doi:10.1002/advs.202502270)
Supplement: Supplementary file 1 — Supporting Information [file ADVS-12-e02270-s001.docx]

**Supporting Information for**

**Novel quaternary ammonium salt-linked STING agonist antibody-drug conjugate: synergistic activation of tumor immunity with mitigated off-target toxicity**

*Yu Long, Borui* *Tang, Fei Xie, Lianqi Liu, Yangyihua Zhou, Jingwen Dong, Jianfeng Wang, Cuicui Sun, Yuting Wang, Ruoqi Li, Na Zhang, Liping Li, Longlong Luo^*^, Junhai Xiao^*^, Wu Zhong^*^, Dian Xiao^*^, Hongbin Deng^*^, Xinbo Zhou^*^*

**This file includes:**

Figure S1. The quality control data of TZ-dSA3-2/4/8/12.

Figure S2. The internalization of TZ-dSA3-12.

Figure S3. TZ-dSA3-12 potently stimulates the STING signaling in cancer cells expressing HER2.

Figure S4. TZ-dSA3-12 reduces off-target toxicity while activates STING in tumor microenvironment.

Figure S5. TZ-dSA3-12 enhances the activation and infiltration of antitumor dendritic cells (DCs) and T cells.

Figure S6. TZ-dSA3-12 activates DCs and polarizes macrophages from M2 to M1 *in vivo.*

Figure S7. H151 abrogates the therapeutic efficacy of TZ-dSA3-12 *in vivo*.

Figure S8. *In vivo* safety assessment of TZ-dSA3-12.

Figure S9. The long-term toxicity assessment of TZ-dSA3-12.

Figure S10. Gating strategies for FASC.

Table S1. Reagents and commercial assay kits used in this study.

Table S2. Antibodies used for immunoblotting.

Table S3. Antibodies used for FACS, IF and IHC analysis.

Table S4. Forward and reverse primers for qPCR.

Chemicals Synthesis

Figures S11-S25 NMR and MS spectra


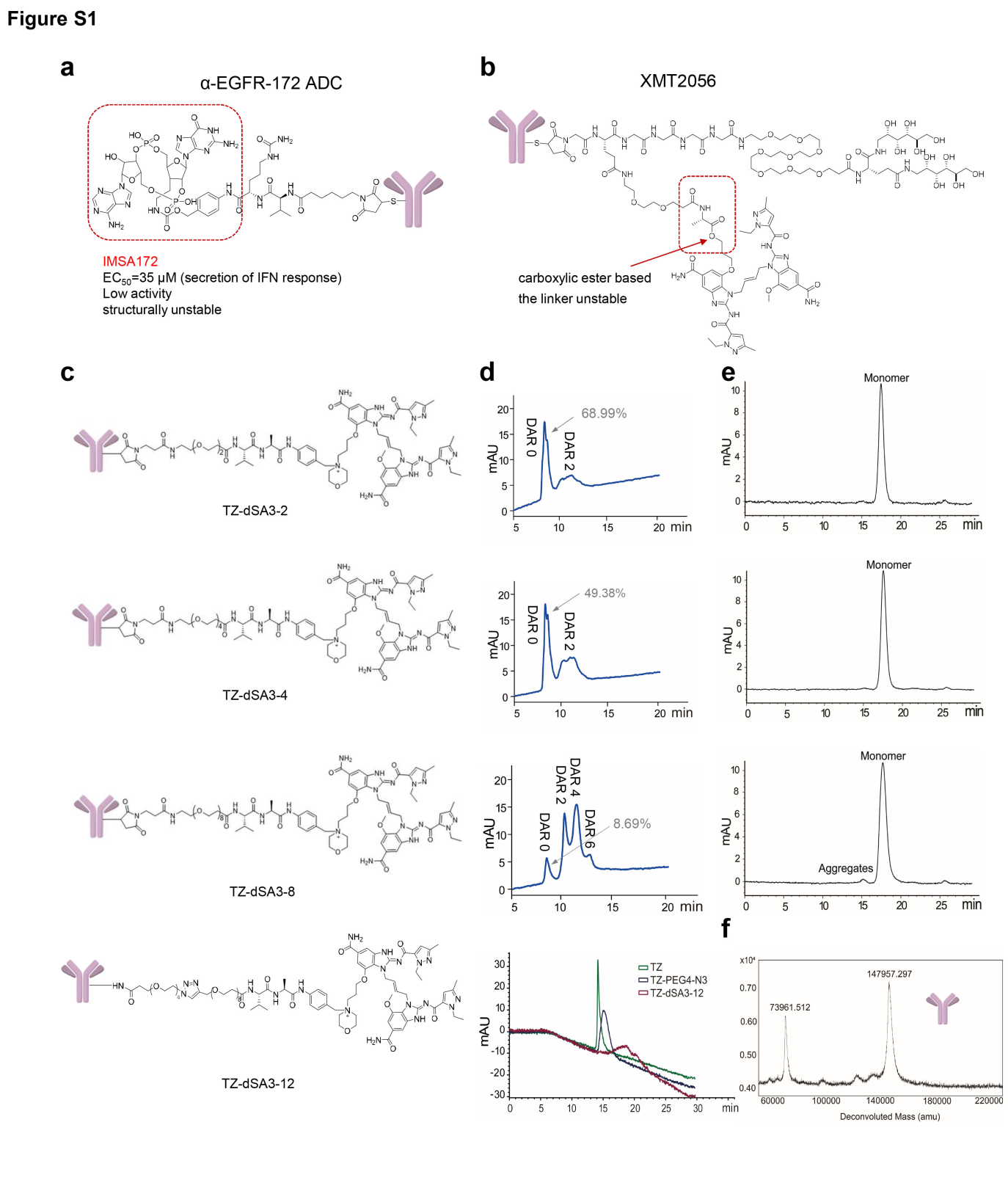


**Figure S1.** The quality control data of TZ-dSA3-2/4/8/12. **a, b.** Diagrams of chemical structures of α-EGFR-172 ADC (**a**) and XMT2056 (**b**). **c.** Diagrams of chemical structures of TZ-dAS3-2/4/8/12 used in this study. **d.** HIC spectroscopies of TZ-dAS3-2/4/8/12. **e.** SEC spectroscopies of TZ-dAS3-2/4/8. **f.** MALDI-TOF analysis of TZ.


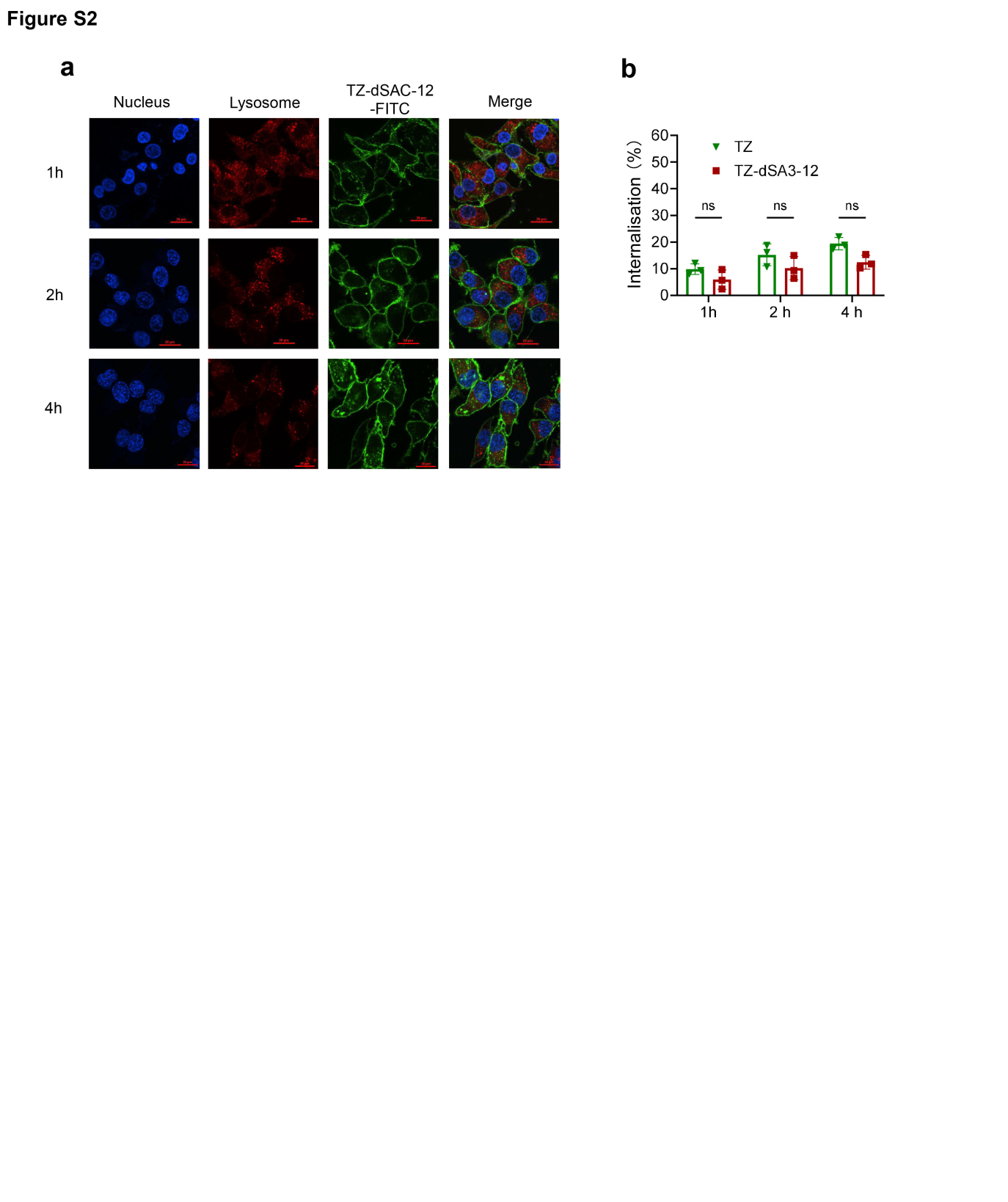


**Figure S2.** The internalization of TZ-dSA3-12. **a.** Confocal microscopy analysis of TZ-dSA3-12 internalization with lysosomes in SKOV3 cells at 1h, 2h, 4h. scale bar = 20 μm. TZ-dSA3-12 were labelled with FITC, lysosome was labelled with Lysotracker red, and nucleus was labelled with DAPI. **b.** Flow cytometry determination of the internalization rate of TZ-dSA3-12 and TZ in SKOV3 cells. For **b**, data were represented three independent experiments as mean ± SD (n = 3). ns, no significant.


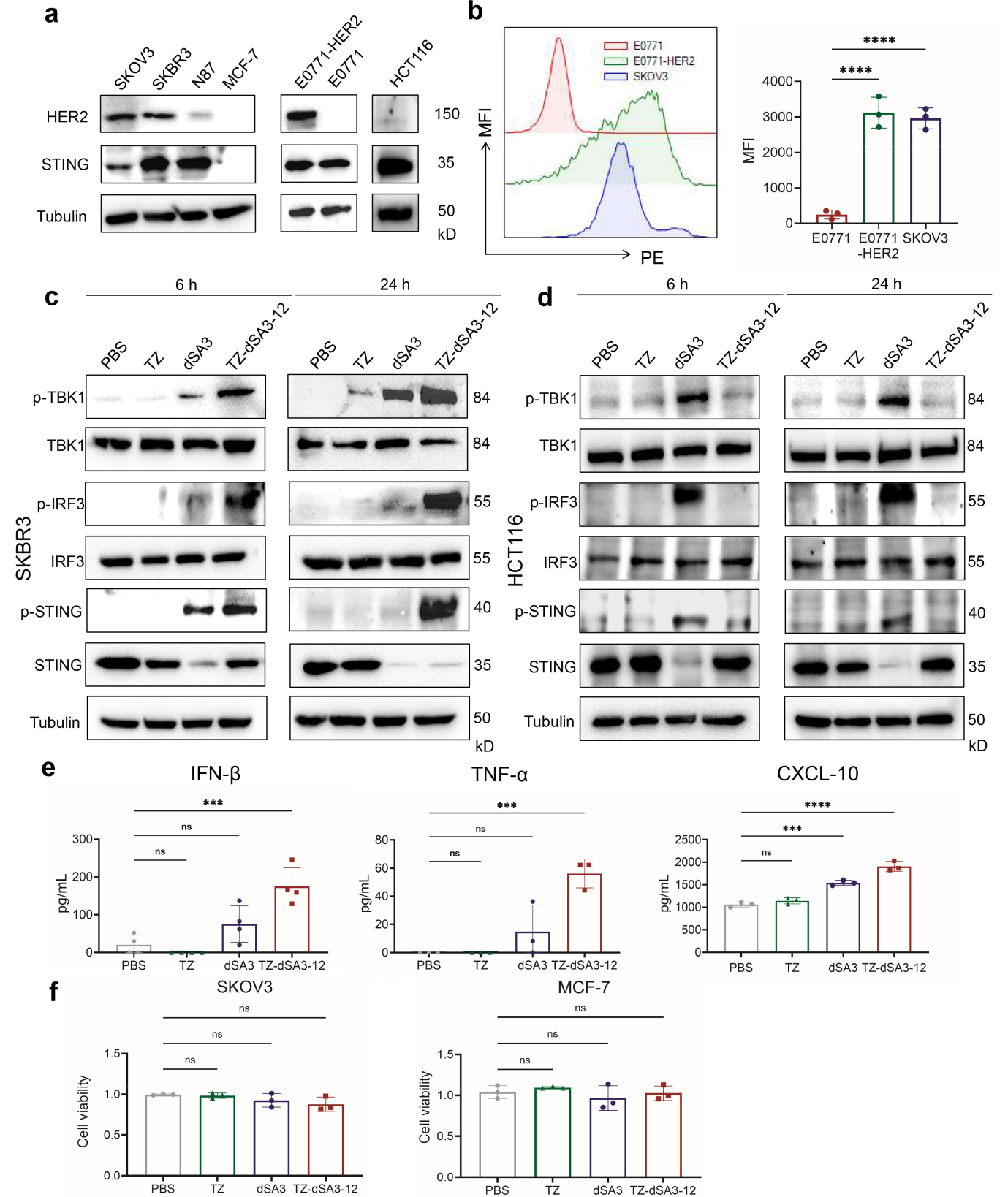


**Figure S3.** TZ-dSA3-12 potently stimulates the STING signaling in cancer cells expressing HER2. **a.** Western blot analysis of STING and HER2 protein expression in various cancer cells. **b.** Flow cytometry analysis of surface expression of HER2 in E0771, E0771-HER2, and SKOV3 cells. **c, d.** SKBR3(**c**) and HCT116 cells(**d**) were treated with PBS, dSA3 (100 nM), TZ (100 nM), and TZ-dSA3-12 (100 nM) for 6 or 24 h; the levels of phospho-TBK1 (p-TBK1), TBK1, phospho-IRF3 (p-IRF3), IRF3, phospho-STING (p-STING), and STING were analyzed by western blot. **e.** Enzyme-linked immunosorbent assay (ELISA) determining the levels of IFN-β, TNF-α, and CXCL-10 in the supernatant of E0771-HER2 cells treated with dSA3(100 nM), TZ (100 nM), and TZ-dSA3-12 (100 nM) for 24 h. **f.** CCK8 assay determining the cytotoxicity effect of TZ-dSA3-12 on SKOV3 and MCF-7 cells. For **e, f,** data were represented three independent experiments as mean ± SD (n = 3); data were analyzed by Ordinary one-way ANOVA (Multiple comparisons), ns, no significant, *^***^p*<0.001, ^****^*p*<0.0001.


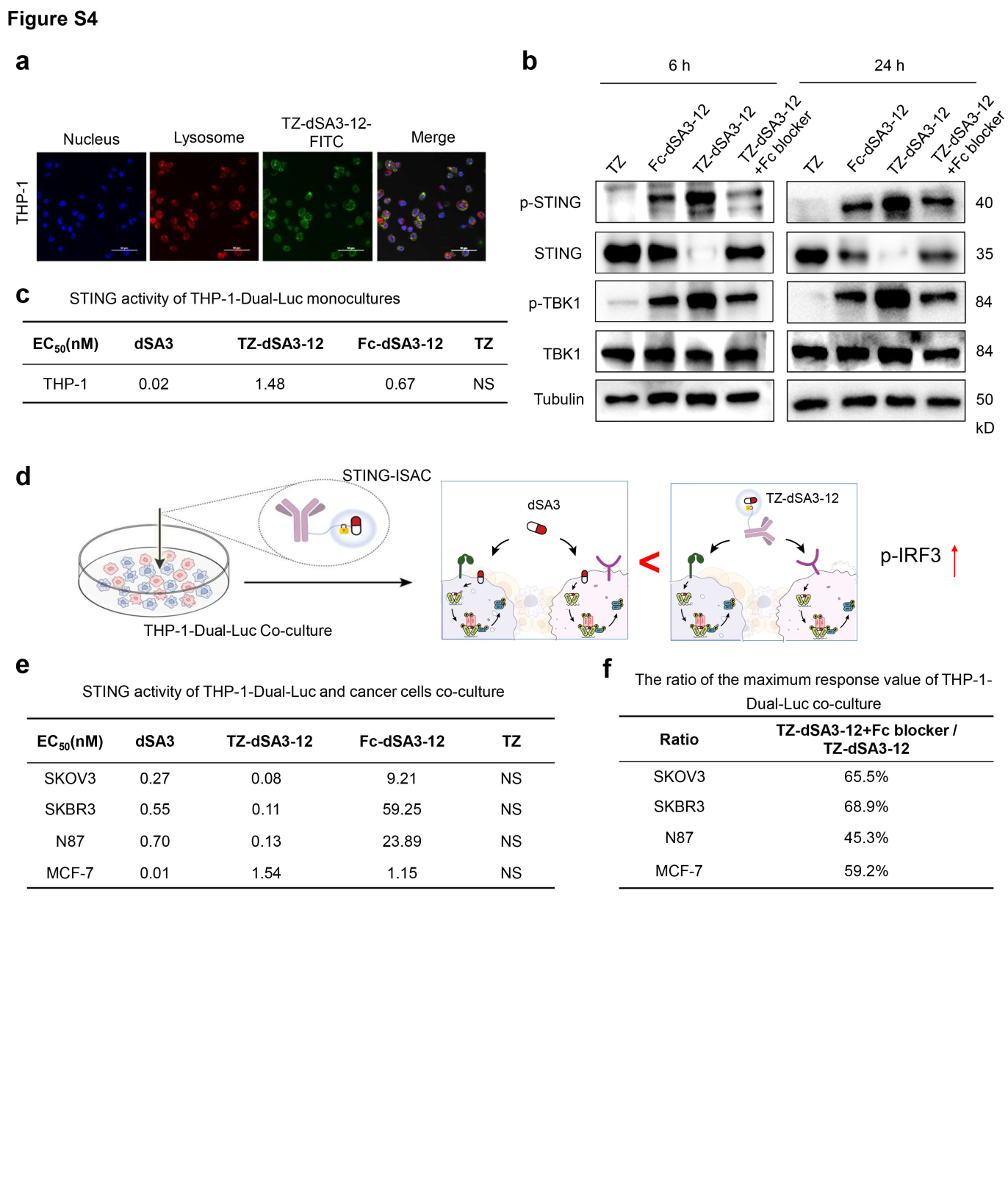


**Figure S4.** TZ-dSA3-12 reduces off-target toxicity while activates STING in tumor microenvironment. **a.** Confocal microscopy analysis of TZ-dSA3-12 internalization and colocalization with lysosomes in THP-1 cells, scale bar =50 μm. TZ-dSA3-12 was labelled with FITC, lysosomes was labelled with Lysotracker red, and nucleus was labelled with DAPI. **b.** THP-1 cells were treated with TZ (100 nM), TZ-dSA3-12 (100 nM), Fc-dSA3-12 (100 nM), and TZ-dSA3-12+Fc blocker for 6 or 24 h; the levels of p-TBK1, TBK1, p-STING, and STING were analyzed by western blot. **c.** dSA3(100 nM), TZ (100 nM), TZ-dSA3-12 (100 nM), and Fc-dSA3-12 (100 nM) stimulated THP-1-Dual-Luc cells for 24 h, the STING activity was measured by luciferase assay. **d.** Schematic diagram of the dSA3- and TZ-dSA3-12-stimulated STING activity in tumor cells and THP-1-Dual-Luc coculture assay**. e.** EC_50_ values of dSA3(100 nM), TZ (100 nM), TZ-dSA3-12 (100 nM), and Fc-dSA3-12 (100 nM)-stimulated STING activity in THP-1-Dual-Luc and cancer cells co-culture assay. **f.** The ratio of the maximum response value of THP-1-Dual-Luc and cancer cells co-culture assay in **e**. For **c, e, f,** data were represented three independent experiments as mean ± SD (n = 3). EC_50_ values were derived using the curve fitting function in GraphPad prism 9.0.


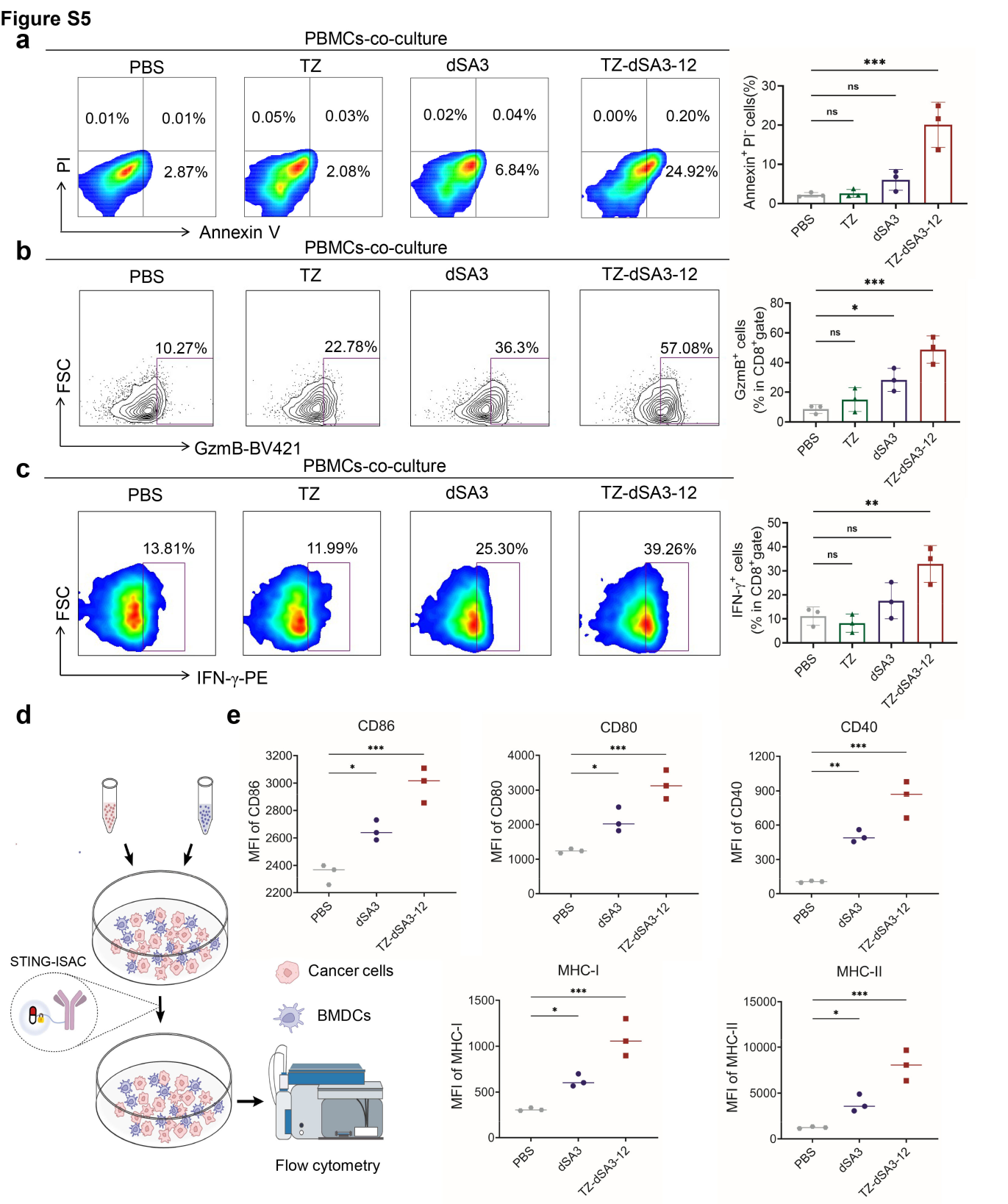


**Figure S5.** TZ-dSA3-12 enhances the activation and infiltration of DCs and T cells. **a.** Flow cytometry analysis of PBMCs-mediated killing of SKBR3 cells using Annexin V and propidium iodide (PI) double staining. Cells that in the early stage of apoptosis with Annexin V- and PI-positive were shown. **b, c.** SKBR3 cells were co-incubated with activated PBMCs, the levels of GzmB and IFN-γ in CD8^+^T cells were examined by flow cytometry. **d.** Schematic of cancer cell-mediated BMDCs activation in response to dSA3 and TZ-dSA3-12. **e.** E0771-HER2 cells were co-cultured with BMDCs for 24 h in the presence of dSA3 or TZ-dSA3-12, the surface expression levels of CD40, CD80, CD86, MHC-II, and MHC-I on CD11c^+^ BMDCs were determined by Flow cytometry. For **a-c, e,** data were represented three independent experiments as mean ± SD (n = 3); data were analyzed by Ordinary one- way ANOVA (Multiple comparisons), ^*^*p*<0.05, ^**^*p*<0.01, ^***^*p*<0.001, ns, not significant.


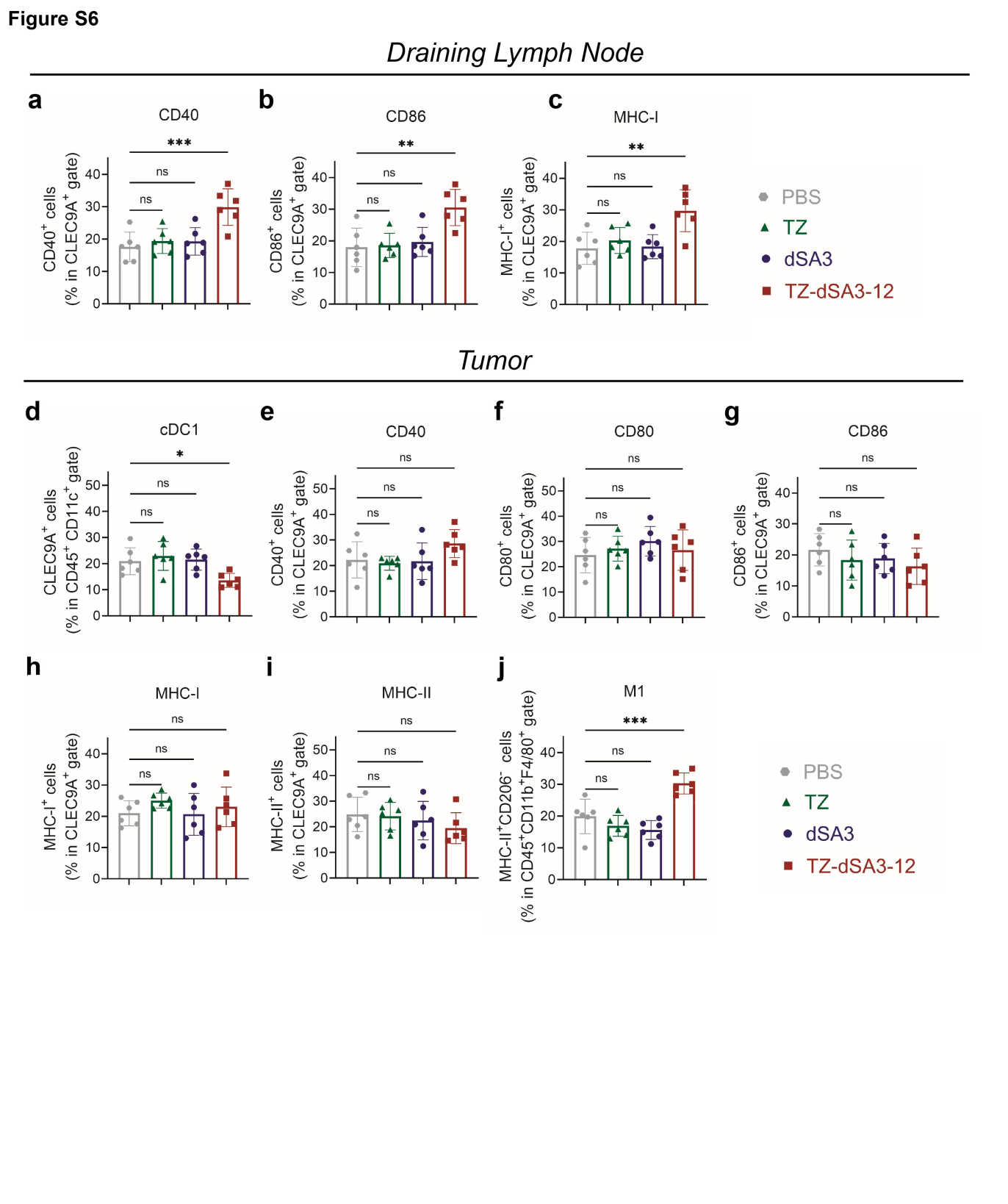


**Figure S6.** TZ-dSA3-12 activates DCs and polarizes macrophages from M2 to M1 *in vivo****.*** FACS analyzed the surface expression levels of CD40 **(a)**, CD86 **(b)**, and MHC-I **(c)** on cDC1 cells in draining lymph nodes, and the numbers of cDC1 in tumors **(d)**, and surface expression levels of CD40 **(e)**, CD80 **(f)**, CD86 **(g)**, MHC-I **(h)** and MHC-Ⅱ **(i)** on cDC1 cells in tumors. FACS analyzing the populations of M1 macrophages **(j)** on macrophages cells. For **a-j**, data were analyzed by Ordinary one- way ANOVA (Multiple comparisons), ^*^*p*<0.05, ^**^*p*<0.01, ^***^*p*<0.001, ns, not significant.


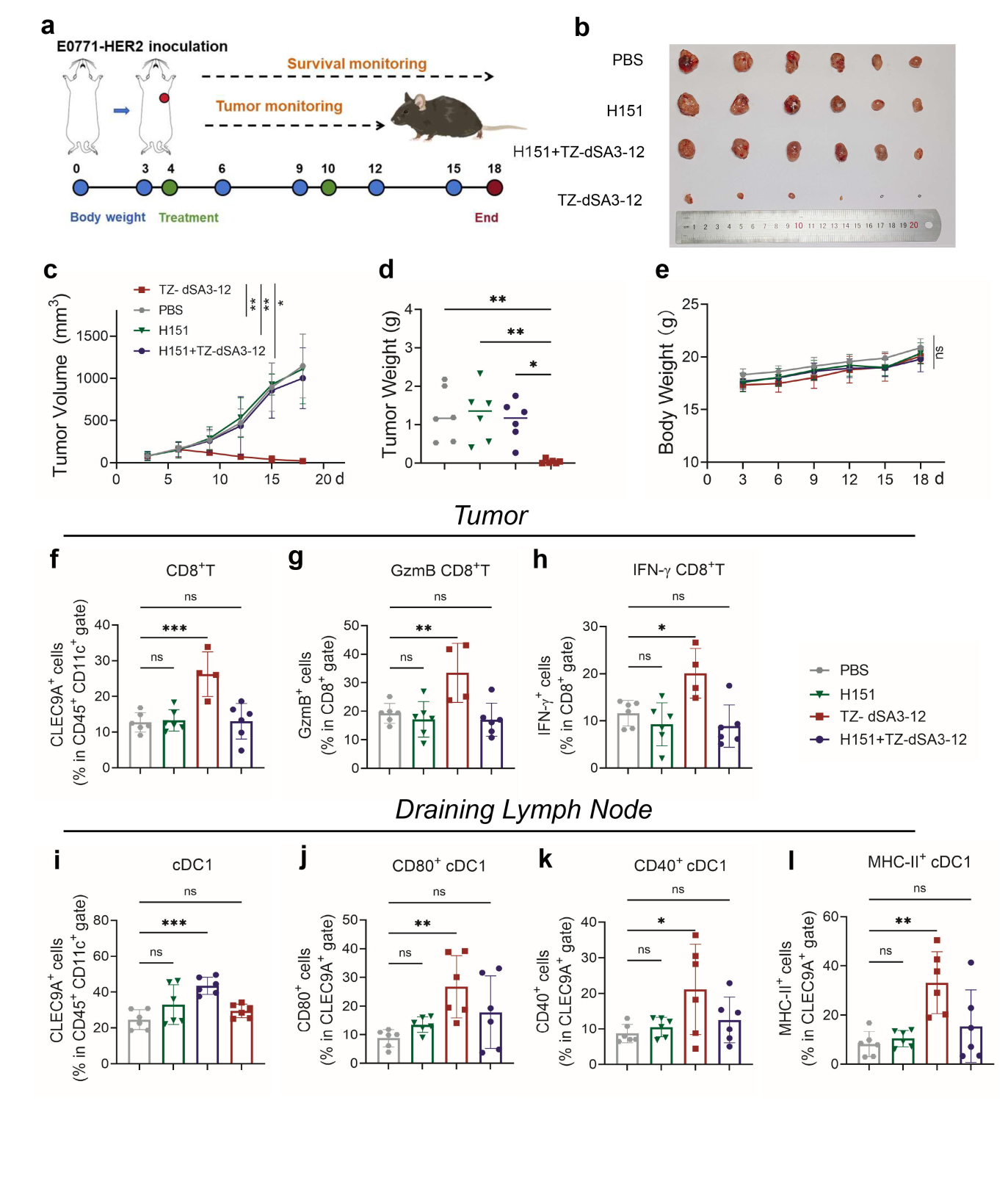


**Figure S7.** H151 abrogates the therapeutic efficacy of TZ-dSA3-12 *in vivo*. **a.** C57BL/6 mice (n = 6) bearing E0771-HER2 tumors were intravenously (*i.v*.) injected with PBS, H151 (750 nM), H151 (750 nM) +TZ-dSA3-12 (3 mg/kg), or TZ-dSA3-12 (3 mg/kg) at the indicated timepoints. **b.** Representative images of excised tumors from each group. **c, d.** Tumor volumes (**c**) and weights (**d**) post-treatment were monitored. **e.** Changes of mice body weight of each group were recorded every three days. **f-h.** Flow cytometry analysis of the numbers of tumor-infiltrating CD8^+^ T cells (**f**), and GzmB (**g**) and IFN-γ (**h**) in CD8^+^T cells; **i-l**. Flow cytometry analyzing the numbers of draining lymph nodes-infiltrating cDC1 (**i**), and surface expression levels of CD80 (**j**), CD40 (**k**), MHC-II (**l**) in cDC1. For **c-l**. data were analyzed by Ordinary one-way ANOVA (Multiple comparisons), ^*^*p*<0.05, ^**^*p*<0.01, ^***^*p*<0.001, ns, not significant.


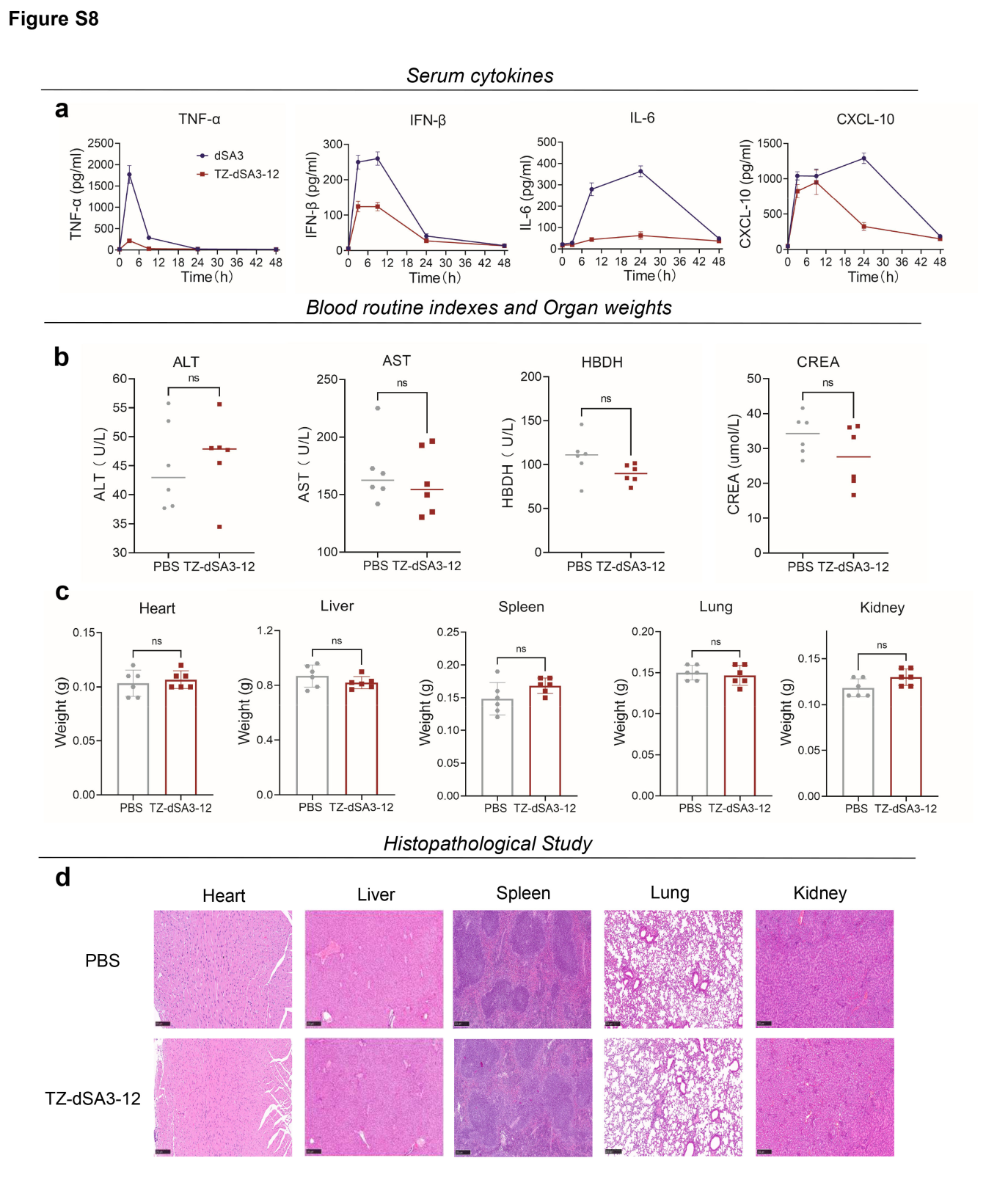
 **Figure S8.** *In vivo* safety assessment of TZ-dSA3-12. C57BL/6 mice bearing E0771-HER2 tumors or C57BL/6 mice were intravenously (*i.v.*) injected with PBS, dSA3 and TZ-dSA3-12. **a.** ELISA quantification of serum levels of TNF-α, IFN-β, IL-6, and CXCL-10 after dSA3(5 mg/kg, *i.v.*) and TZ- dSA3-12 (3 mg/kg, *i.v.*) treatment. The data were presented as mean ± SD, n=3. **b.** After TZ-dSA3-12 (6 mg/kg, *i.v.)* treatment for 16 days, heart, liver, spleen, lung, and kidney of each group were taken out for weighing. **c.** The serum biochemistry indices ALT, AST, HBDH, and CREA of each group after TZ-dSA3-12 (6 mg/kg *i.v.*) treatment were measured. **d.** Representative hematoxylin-eosin (H&E) staining of the heart, liver, spleen, lung, and kidney. For **b-d**, data were presented as mean ± SD, n=6 per group; data were analyzed by t-test, ns, not significant.


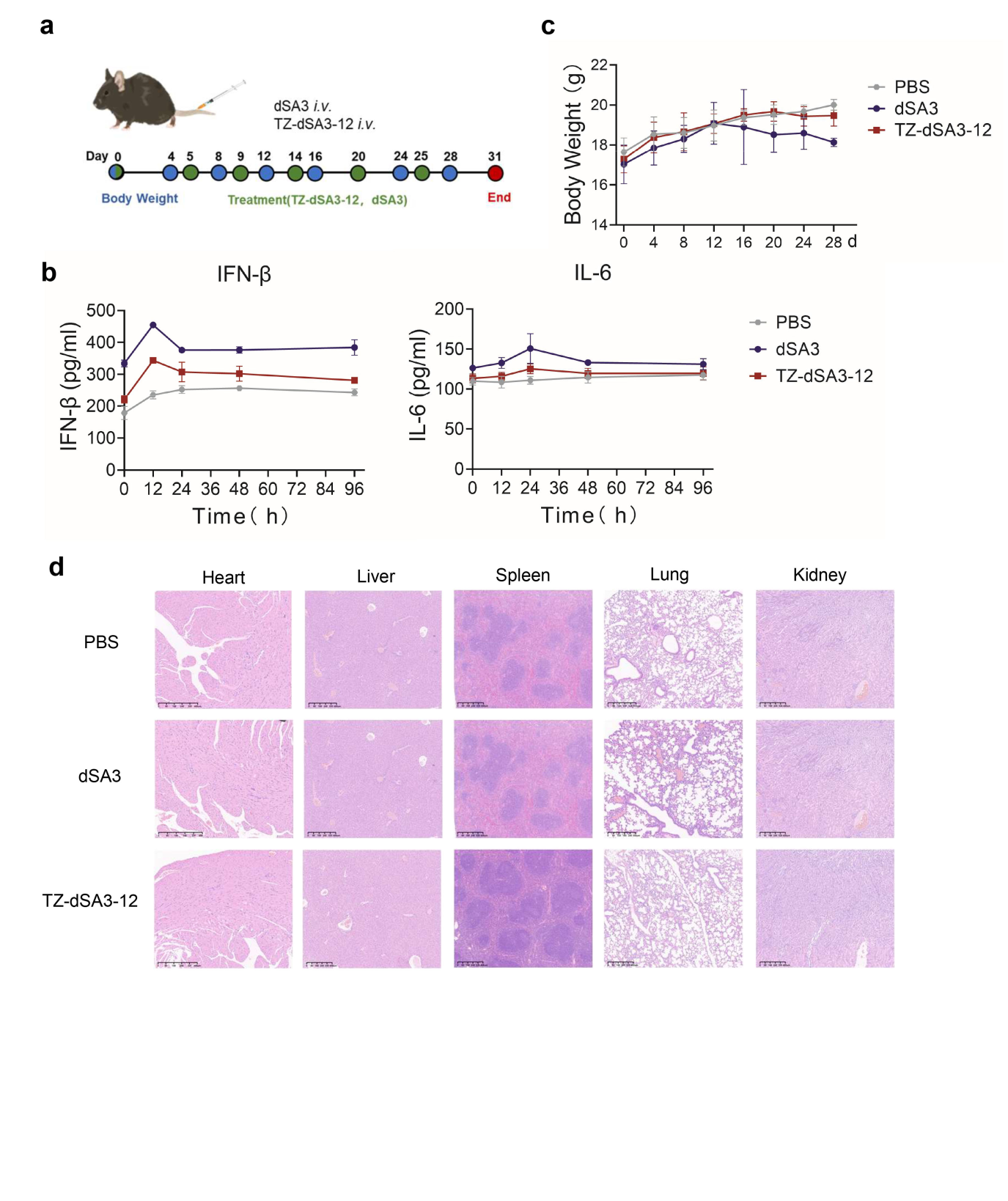
 **Figure S9.** The long-term safety assessment of TZ-dSA3-12. **a.** C57BL/6 mice bearing E0771-HER2 tumors or C57BL/6 mice were intravenously (*i.v.*) injected with PBS, dSA3(1.5 mg/kg) and TZ-dSA3-12(3 mg/kg). **b.** Serum levels of IFN-β and IL-6 were injected with and TZ-dSA3-12 at the indicated time point using ELISA. The data were presented as mean ± SD, n=3. **c.** Body weight changes of tumor-bearing mice during the tumor rechallenge. **d.** Representative hematoxylin-eosin (H&E) staining of the heart, liver, spleen, lung, and kidney. For **c, d**, data were presented as mean ± SD, n=6 per group.


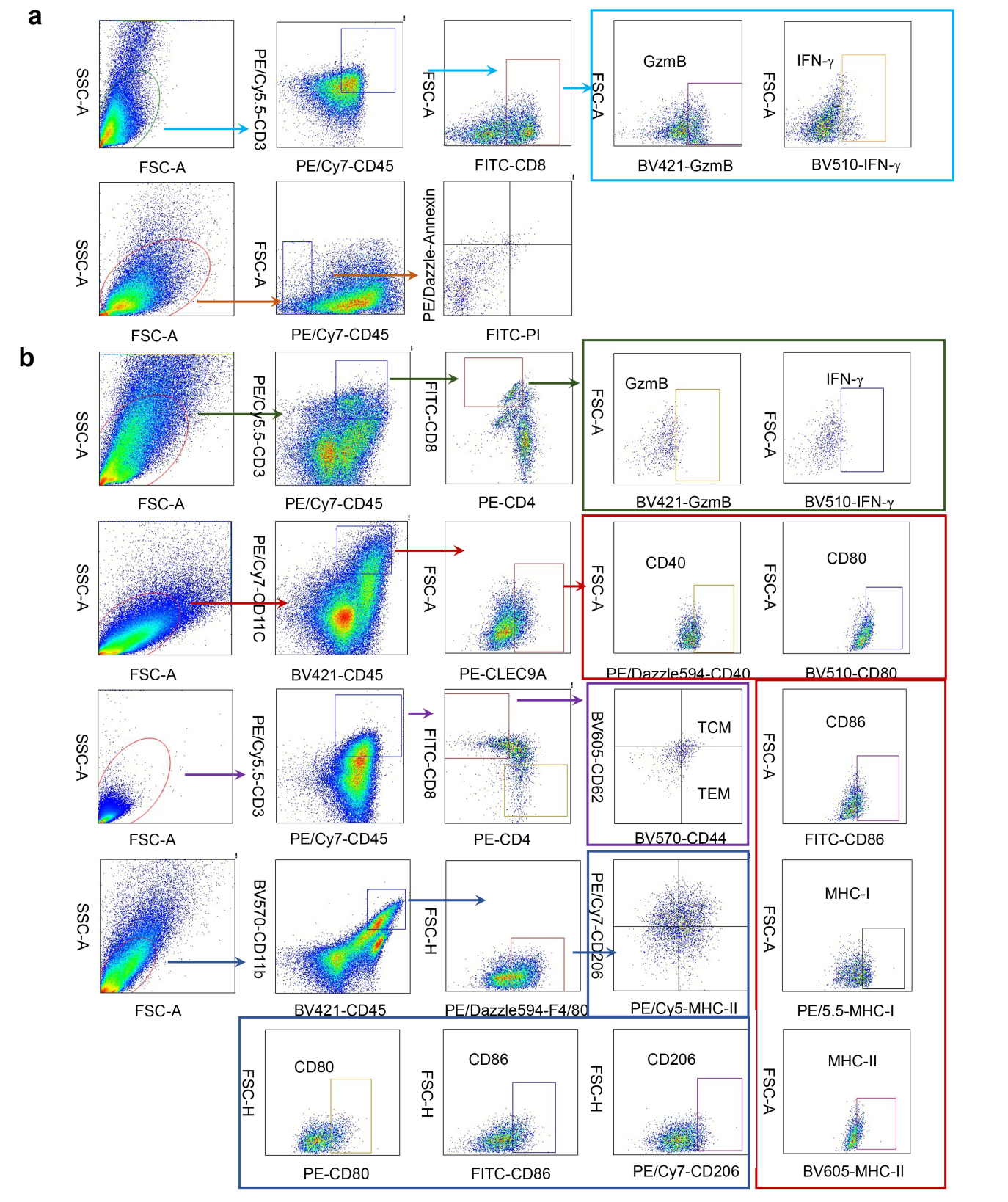


**Figure S10.** Gating strategies for FASC. **a.** Gating strategy for CD8^+^ T cell, IFN-γ, GzmB, and apoptosis in PBMC cells co-culture assay. **b.** Flow cytometry gating strategies for analysis of tumor-infiltrating CD8^+^ T, DCs, and macrophages were shown.

**Table S1**. Reagents and commercial assay kits used in this study

| REAGENTS/COMMERCIAL KITS | SOURCE | IDENTIFIER |
| --- | --- | --- |
| TRNzol Universal Reagent | TIANGEN | Cat# DP424 |
| UniPeak U+ One Step RT-qPCR SYBR Green Kit | Vazyme | Cat# Q226-01 |
| Mouse CXCL10/IP-10/CRG-2 ELISA Kit | R&D Systems | Cat# DY466-05 |
| Mouse IFN-beta Quantikine ELISA Kit | R&D Systems | Cat# MIFNB0 |
| Mouse TNF-alpha Quantikine ELISA Kit | R&D Systems | Cat# MTA00B |
| Human IFN-beta Quantikine ELISA Kit | R&D Systems | Cat# DIFNB0 |
| Human CXCL10/IP-10 Quantikine ELISA Kit | R&D Systems | Cat# DIP100 |
| Human TNF-alpha Quantikine ELISA Kit | R&D Systems | Cat# MTA00D |
| Human IL-6 Quantikine ELISA Kit | R&D Systems | Cat# D6050B |
| Cell Counting Kit-8 (CCK-8) | TargetMol | Cat# C0005 |
| QUANTI-LucTM4 Lucia/Gaussia | Invivogen | Cat# rep-qlc4lg5 |
| Human TruStain FcX™ (Fc Receptor Blocking Solution) | Biolegend | Cat# 422302 |
| Cathepsin B from Human Liver | Sigma | Cat# C8571 |

**Table S2**. Antibodies used for Western blot

| ANTIBODY | SOURCE | IDENTIFIER |
| --- | --- | --- |
| Phospho-STING (Ser366) (D7C3S) Rabbit mAb | Cell Signaling Technology | Cat# 19781 |
| Phospho-STING (Ser365) (D8F4W) Rabbit mAb | Cell Signaling Technology | Cat# 72971 |
| Phospho-IRF-3 (Ser396) (4D4G) Rabbit mAb | Cell Signaling Technology | Cat# 4947S |
| STING (D2P2F) Rabbit mAb | Cell Signaling Technology | Cat# 13647 |
| IRF-3 (D83B9) Rabbit mAb | Cell Signaling Technology | Cat# 4302 |
| Phospho-TBK1/NAK (Ser172) XP Rabbit mAb | Cell Signaling Technology | Cat# 5483 |
| TBK1/NAK (D1B4) Rabbit mAb | Cell Signaling Technology | Cat# 3504 |
| HER2/ErbB2 (D8F12) XP Rabbit mAb | Cell Signaling Technology | Cat# 4290 |
| GAPDH Rabbit mAb | Cell Signaling Technology | Cat# 5174 |
| β-Tubulin Antibody | Cell Signaling Technology | Cat# 2146 |

**Table S3**. Antibodies used for flow cytometry

| ANTIBODY | SOURCE | IDENTIFIER |
| --- | --- | --- |
| PE/Cyanine7 anti-mouse CD11c antibody | Biolegend | Cat# 117318 |
| PE/Dazzle™ 594 anti-mouse CD40 Antibody | Biolegend | Cat# 124630 |
| TITC anti-mouse CD86 antibody | Biolegend | Cat# 105006 |
| PE anti-mouse CD80 antibody | Biolegend | Cat# 104707 |
| PerCP/Cyanine5.5 anti-mouse H-2Kb Antibody | Biolegend | Cat# 116516 |
| Brilliant Violet 605™ anti-mouse I-A/I-E Antibody | Biolegend | Cat# 107639 |

| PE/Cyanine7 anti-mouse CD45 antibody | Biolegend | Cat# 157205 |
| --- | --- | --- |
| PerCP/Cyanine5.5 anti-mouse CD3 antibody | Biolegend | Cat# 100217 |
| FITC anti-mouse CD8a antibody | Biolegend | Cat# 100705 |
| PE anti-mouse CD4 antibody | Biolegend | Cat# 100407 |
| FITC anti-mouse CD25 antibody | Biolegend | Cat# 101907 |
| Brilliant Violet 510™ anti-mouse IFN-γ antibody | Biolegend | Cat# 505841 |
| PE anti-mouse/human CD11b antibody | Biolegend | Cat# 101207 |
| FITC anti-mouse F4/80 antibody | Biolegend | Cat# 123107 |
| Brilliant Violet 421™ anti-mouse CD45 antibody | Biolegend | Cat# 103133 |
| Brilliant Violet 421™ anti-human/mouse  Granzyme B Recombinant antibody | Biolegend | Cat# 396414 |
| Brilliant Violet 421™ anti-mouse CD206 (MMR)antibody | Biolegend | Cat# 141717 |
| Brilliant Violet 421™ anti-mouse FOXP3 antibody | Biolegend | Cat# 126419 |
| Brilliant Violet 421™ anti-mouse CD69 antibody | Biolegend | Cat# 104527 |
| Brilliant Violet 421™ anti-mouse Ly-6C antibody | Biolegend | Cat# 128031 |
| Brilliant Violet 570™ anti-mouse Ly-6G antibody | Biolegend | Cat# 127629 |
| Brilliant Violet 510™ anti-mouse CD69 antibody | Biolegend | Cat# 310936 |
| Brilliant Violet 605™ anti-mouse CD62L Antibody | Biolegend | Cat# 104438 |
| PE/Dazzle 594™ anti-mouse CD279 (PD-1) Antibody | Biolegend | Cat# 135228 |
| Brilliant Violet 570™ anti-mouse/human CD44 Antibody | Biolegend | Cat# 103037 |

**Table S4**. Primers for RT-qPCR

| GENE | SEQUENCE |
| --- | --- |
| qPCR: Mouse TNF forward | CCTCTCATCAGTTCTATGGCCC |
| qPCR: Mouse TNF reverse | GTCTTTGAGATCCATGCCGTTG |
| qPCR: Mouse CXCL10 forward | TCATTTTCTGCCTCATCCTGCT |
| qPCR: Mouse CXCL10 reverse | TCTGCAAGCTGAAGGGATTTCT |
| qPCR: Mouse IFNβ forward | ATGGAGATGACGGAGAAGATGC |
| qPCR: Mouse IFNβ reverse | TTCAGAAACACTGTCTGCTGGT |
| qPCR: Mouse GAPDH forward | TGGCCTCCAAGGAGTAAGAAAC |
| qPCR: Mouse GAPDH reverse | ATTCAAGAGAGTAGGGAGGGCT |
| qPCR: Human TNF forward | GCCCATGTTGTAGCAAACCC |
| qPCR: Human TNF reverse | TGAGGTACAGGCCCTCTGAT |
| qPCR: Human CXCL10 forward | CCACGTGTTGAGATCATTGCT |
| qPCR: Human CXCL10 reverse | TGCATCGATTTTGCTCCCCT |
| qPCR: Human IFNβ forward | AGTAGGCGACACTGTTCGTG |
| qPCR: Human IFNβ reverse | GCCTCCCATTCAATTGCCAC |
| qPCR: Human GAPDH forward | TGCACCACCAACTGCTTAGC |
| qPCR: Human GAPDH reverse | GGCATGGACTGTGGTCATGAG |

Chemicals Synthesis

*Synthesis of the TZ-dSA3-2/4/8:* The synthetic route for TZ-dSA3-2/4/8 is depicted in the Supporting Information Appendix, Scheme S1.

**

**Scheme S1.** Synthesis of antibody conjugates TZ-dSA3-2/4/8. Reagents and conditions: (a) L-Val-L- Ala-PAB, EDCI, HOBt, DIPEA, anhydrous DMF, r.t., 18 h; (b) SOCl_2_, -15 ºC, 1 h; (c) dSA3, TBAI, DIPEA, 55 ºC, 14 d; (d) TZ, TCEP, NAC, H_2_O, r.t.

*Synthesis of M1:* Starting with maleimide-dipolyethylene glycol-carboxylic acid (500.00 mg, 1.52 mmol), this material was added to a 100 mL flask and dissolved in 20 mL of anhydrous DMF. At room temperature with stirring, EDCI (437.08 mg, 2.28 mmol), HOBt (247.27 mg, 1.83 mmol), and DIPEA (294.44 mg, 2.28 mmol) were added sequentially. The reaction was carried out at room temperature under an argon atmosphere for 2 h. L-Val-L-Ala-PAB (445.91 mg, 1.52 mmol) was then added to the reaction mixture. The progress of the reaction was monitored by TLC. After the reaction was complete, the solvent was removed using a rotary evaporator, and the product was purified by column chromatography to obtain a colorless oily substance (773.98 mg, 84.35% yield). ¹H NMR (600 MHz, DMSO-d₆) δ 9.99 (s, 1H), 8.21 (dd, J = 11.9, 7.0 Hz, 1H), 8.03 (t, J = 5.5 Hz, 1H), 7.90 (d, J = 8.7 Hz, 1H), 7.59 (dd, J = 16.4, 8.5 Hz, 2H), 7.36 (d, J = 8.5 Hz, 1H), 7.24 (d, J = 8.5 Hz, 1H), 7.00 (s, 2H), 4.71 (s, 1H), 4.41–4.36 (m, 1H), 4.33 (s, 1H), 4.23–4.19 (m, 1H), 3.61–3.57 (m, 4H), 3.48–3.45 (m, 4H), 3.34 (t, J = 5.9 Hz, 2H), 3.24 (s, 1H), 3.14 (q, J = 5.8 Hz, 2H), 2.46 (dd, J = 14.1, 7.2 Hz, 1H), 2.39 (dd, J = 13.6, 7.2 Hz, 1H), 2.33 (t, J = 7.3 Hz, 2H), 1.96 (dq, J = 13.6, 6.8 Hz, 1H), 1.31 (d, J = 7.1 Hz, 3H), 0.87 (d, J = 6.8 Hz, 3H), 0.83 (d, J = 6.8 Hz, 3H). Calculated for C₂₉H₄₂N₅O₉⁺ [M+H] ⁺ 604.6730; found 604.3130.

*Synthesis of M2:* Starting with maleimide-tetraethylene glycol-carboxylic acid (500.00 mg, 1.20 mmol), the synthesis method was similar to that of intermediate M1, yielding a colorless oily substance (667.26 mg, 80.38% yield). ¹H NMR (600 MHz, DMSO-d₆) δ 10.01 (s, 1H), 8.21 (dd, J = 11.8, 7.0 Hz, 1H), 8.04 (t, J = 5.5 Hz, 1H), 7.91 (d, J = 8.7 Hz, 1H), 7.60 (dd, J = 16.7, 8.5 Hz, 2H), 7.37 (d, J = 8.5 Hz, 1H), 7.24 (d, J = 8.5 Hz, 1H), 7.00 (s, 2H), 4.72 (s, 1H), 4.40 (p, J = 7.1 Hz, 1H), 4.33 (s, 1H), 4.21 (dd, J = 8.3, 7.0 Hz, 1H), 3.59 (dd, J = 8.2, 3.0 Hz, 8H), 3.51–3.46 (m, 12H), 3.36 (t, J = 5.9 Hz, 2H), 3.25 (s, 1H), 3.18–3.12 (m, 2H), 2.47 (dd, J = 14.2, 7.2 Hz, 1H), 2.42–2.35 (m, 1H), 2.33 (t, J = 7.3 Hz, 2H), 1.97 (dq, J = 13.5, 6.7 Hz, 1H), 1.31 (d, J = 7.1 Hz, 3H), 0.88 (d, J = 6.8 Hz, 3H), 0.84 (d, J = 6.8 Hz, 3H). Calculate for C₃₃H₅₀N₅O₁₁⁺ [M+H] ⁺ 692.7790; found 692.3657.

*Synthesis of M3:* Starting with maleimide-octoethylene glycol-carboxylic acid (500.00 mg, 0.84 mmol), the synthesis method was similar to that of intermediate M1, yielding a colorless oily substance (560.32 mg, 76.85% yield). ¹H NMR (600 MHz, DMSO-d₆) δ 9.98 (s, 1H), 8.25–8.14 (m, 1H), 8.03 (t, J = 5.4 Hz, 1H), 7.90 (d, J = 8.7 Hz, 1H), 7.60 (dd, J = 16.5, 8.5 Hz, 2H), 7.37 (d, J = 8.5 Hz, 1H), 7.24 (d, J = 8.4 Hz, 1H), 7.00 (s, 2H), 4.71 (s, 1H), 4.39 (t, J = 7.1 Hz, 1H), 4.33 (s, 1H), 4.21 (dd, J = 8.2, 7.2 Hz, 1H), 3.59 (t, J = 6.0 Hz, 4H), 3.49 (dd, J = 9.4, 4.5 Hz, 28H), 3.36 (t, J = 5.9 Hz, 2H), 3.25 (s, 1H), 3.15 (q, J = 5.8 Hz, 2H), 2.47 (dd, J = 14.2, 7.1 Hz, 1H), 2.41–2.36 (m, 1H), 2.33 (t, J = 7.3 Hz, 2H), 1.97 (dq, J = 13.6, 6.8 Hz, 1H), 1.31 (d, J = 7.1 Hz, 3H), 0.88 (d, J = 6.8 Hz, 3H), 0.84 (d, J = 6.8 Hz, 3H). Calculate for C₄₁H₆₆N₅O₁₅⁺ [M+H] ⁺ 868.9910; found 868.3524.

*Synthesis of M4:* Starting with intermediate M1 (300.00 mg, 0.50 mmol), this material was added to a 100 mL round-bottom flask and dissolved in a mixed solvent of 5 mL anhydrous DMF and 15 mL anhydrous dichloromethane. The mixture was cooled to –15 °C and stirred while thionyl chloride (178.46 mg, 1.50 mmol) dissolved in 5 mL anhydrous dichloromethane was slowly added. The reaction was maintained at –15 °C for 30 min, monitored by TLC. After the reaction was complete, the solvent was removed using a rotary evaporator, and the product was purified by column chromatography to obtain a pale-yellow oily substance (142.09 mg, 45.68% yield). ¹H NMR (600 MHz, DMSO- d₆) δ 10.09 (s, 1H), 8.28 (d, J = 6.9 Hz, 1H), 8.08 (t, J = 5.6 Hz, 1H), 7.96 (d, J = 8.7 Hz, 1H), 7.66 (d, J = 8.5 Hz, 2H), 7.42 (d, J = 8.5 Hz, 2H), 7.06 (s, 2H), 4.77 (s, 2H), 4.45 (p, J = 7.1 Hz, 1H), 4.27 (dd, J = 8.5, 7.0 Hz, 1H), 3.68–3.62 (m, 5H), 3.57–3.50 (m, 6H), 3.40 (dd, J = 7.5, 4.2 Hz, 3H), 3.30 (s, 1H), 3.20 (dd, J = 11.4, 5.7 Hz, 3H), 2.52 (dd, J = 14.1, 7.2 Hz, 1H), 2.48–2.42 (m, 1H), 2.39 (t, J = 7.3 Hz, 2H), 2.02 (dq, J = 13.6, 6.7 Hz, 1H), 1.37 (d, J = 7.1 Hz, 3H), 0.93 (d, J = 6.8 Hz, 3H), 0.89 (d, J = 6.8 Hz, 3H). Calculated for C₂₉H₄₁ClN₅O₈⁺ [M+H] ⁺ 622.2565; found 622.2618.

*Synthesis of M5:* Starting with intermediate M2 (345.89 mg, 0.50 mmol), the synthesis method was similar to that of intermediate M4, yielding a pale-yellow oily substance (147.80 mg, 41.62% yield). ¹H NMR (600 MHz, DMSO-d₆) δ 9.99 (s, 1H), 8.21 (dd, J = 11.9, 7.0 Hz, 1H), 8.03 (t, J = 5.5 Hz, 1H), 7.90 (d, J = 8.7 Hz, 1H), 7.59 (dd, J = 16.4, 8.5 Hz, 2H), 7.36 (d, J = 8.5 Hz, 1H), 7.24 (d, J = 8.5 Hz, 1H), 7.00 (s, 2H), 4.71 (s, 1H), 4.42–4.36 (m, 1H), 4.33 (s, 1H), 4.24–4.17 (m, 1H), 3.62–3.55 (m, 4H), 3.50–3.43 (m, 4H), 3.34 (t, J = 5.9 Hz, 2H), 3.14 (q, J = 5.8 Hz, 2H), 2.51–2.49 (m, 1H), 2.46 (dd, J = 14.1, 7.2 Hz, 1H), 2.39 (dd, J = 13.6, 7.2 Hz, 1H), 2.33 (t, J = 7.3 Hz, 2H), 1.96 (dq, J = 13.6, 6.8 Hz, 1H), 1.31 (d, J = 7.1 Hz, 3H), 0.87 (d, J = 6.8 Hz, 3H), 0.83 (d, J = 6.8 Hz, 3H). Calculated for C₃₃H₄₉ClN₅O₁₀⁺ [M+H] ⁺ 710.3090; found 710.3159.

*Synthesis of M7:* Starting with dSA3 (20 mg, 23.53 mmol) and intermediate M4 (16.10 mg, 25.88 mmol), these were added to a 1.5 μL microcentrifuge tube and dissolved in 300 μL of anhydrous DMF. DIPEA (3.49 mg, 25.88 mmol) and TBAI (4.35 mg, 11.77 mmol) were then added. The reaction was maintained at 55 °C for 7 days, monitored by TLC. After the reaction was complete, the solvent was removed using a rotary evaporator, and the product (quaternary ammonium chloride or iodide) was purified by column chromatography to obtain a white solid (14.95 mg, 38.41% yield). Purity > 95%. 1H-NMR (600 MHz, DMSO-d6): δ 10.222(br., 1H), 8.007-7.883 (m, 4H), 7.756-7.720 (m, 3H), 7.693-7.635 (m, 2H), 7.499-7.344 (m, 3H), 7.245-7.198 (m, 3H), 6.521-6.445 (m, 1H), 5.788-5.397 (m, 2H), 5.397 (s, 1H), 4.991-4.871 (m, 3H), 4.650-4.496 (m, 5H), 4.358 (d, J = 4.8 Hz, 1H), 4.240-4.179 (m, 2H), 4.058 (s, 2H), 3.871 (br., 1H), 3.827 (s, 1H), 3.673 (s, 1H), 3.592 (s, 3H), 3.514-3.470 (m, 15H), 3.172 (br., 2H), 2.277-2.245 (m, 2H), 2.110 (s, 5H), 2.028-1.913 (m, 3H), 1.297-1.287 (m, 7H), 0.932-0.828 (m, 10H). Calculated for C₇₁H₉₈N₁₈O₁₅⁺ [M+H] ⁺ 1436.6906; found 1435.6939.

*Synthesis of M8:* Starting with dSA3 (20 mg, 23.53 mmol) and intermediate M5 (18.38 mg, 25.88 mmol), the synthesis method was similar to that of intermediate M7, yielding a white solid (12.54 mg, 31.33% yield). Purity > 95%. Calculated for C₇₅H₁₀₀N₁₈O₁₇⁺ [M+H] ⁺ 1524.7430; found 1523.7432.

*Synthesis of M9:* Starting with dSA3 (20 mg, 23.53 mmol) and intermediate M6 (22.94 mg, 25.88 mmol), the synthesis method was similar to that of intermediate M7, yielding a white solid (13.61 mg, 34.96% yield). Purity > 95%. Calculated for C₈₃H₁₁₅N₁₃O₂₁⁺ [M+H]⁺ 1700.8479; found 1700.8507.

*Generation of the TZ-dSA3-2/4/8:* Trastuzumab solution was precisely transferred to the reaction flask using a pipette (1.00 eq). Under slow stirring at room temperature, a 1 mg/mL TCEP (tris(2-carboxyethyl) phosphine) aqueous solution containing 4.00 eq was slowly added dropwise, and the reaction was maintained for 2 h. The required mass of small molecule raw material intermediates M7 to M9 was calculated (10.00 eq), and these were dissolved in an organic solvent (DMAC, N, N-dimethylacetamide) at 8% of the total reaction liquid volume. Under slow stirring at room temperature, the small molecule solution was slowly added dropwise to the reaction solution using a pipette, and the reaction was maintained for 4 h. Next, the reaction was terminated, and the unreacted raw materials M7 to M9 were capped. The small molecule N-acetylcysteine with a reducing thiol group was weighed (15 eq) and dissolved in an aqueous solution (1.00 mg/mL). This solution was slowly added dropwise to the reaction solution using a pipette, and the reaction was maintained for an additional 30 min. After the reaction was terminated, the reaction solution was concentrated to a volume of 5 mL using an ultrafiltration tube. Then, using GE Healthcare's AKTA Purifier and a hydrophobic interaction chromatography column (Butyl-5 mL), with the aforementioned Buffer A and Buffer B, the unreacted naked antibody in the reaction solution was separated and purified. After the reaction solution was concentrated by ultrafiltration, it was again purified using GE Healthcare's AKTA Purifier and a size exclusion chromatography column (G25-25 mL) to remove small molecule substances from the reaction solution. The collected solution was filtered through a 0.22 µm pore filter membrane for sterilization and aliquoted, yielding the quaternary ammonium chloride TZ-dSA3-2/4/8, which were stored at –80 °C for future use.

*Synthesis of TZ-dSA3-12:* The synthetic route for TZ-dSA3-12 is depicted in the Supporting Information Appendix, Scheme S2.

**Scheme S2.** Synthesis of antibody conjugates TZ-dSA3-12. Reagents and conditions: (a) L-Val-L- Ala-PAB, NHS, DIC, DIPEA, anhydrous DMF, r.t., 18 h; (b) SOCl_2_, -15 ºC, 1 h; (c) dSA3, TBAI, DIPEA, 55 ºC, 14 d; (d) TZ, (2,5-dioxopyrrolidin-1-yl) 3-[2-[2-[2-(2-azidoethoxy) ethoxy] ethoxy] ethoxy] propanoate, Cu2SO4.5H20, NaVc, THPTA, HEPES, r.t.

*Synthesis of M10:* The synthesis began with the addition of propargyl octa (ethylene glycol) carboxylic acid (500.00 mg, 1.14 mmol) to a 25 mL flask, which was then dissolved in 5 mL of anhydrous DMF and stirred at room temperature. NHS (120.3 mg, 1.05 mmol) and DIC (179.6 mg, 1.4 mmol) were subsequently added, and the reaction was carried out under an argon atmosphere for 12 h at room temperature. *L*-Val-*L*-Ala-PAB (290.6 mg, 0.96 mmol) was introduced into the reaction mixture, and the progress was monitored by TLC. Once the reaction was complete, the solvent was removed using a rotary evaporator, and the product was purified by column chromatography, yielding a pale-yellow oily product (564 mg, 80.2% yield). The ¹H NMR (600 MHz, Chloroform-d) data are as follows: δ 8.66 (s, 1H), 7.67 (d, J = 8.5 Hz, 2H), 7.32–7.23 (m, 3H), 7.07 (t, J = 6.0 Hz, 2H), 4.67 (p, J = 7.3 Hz, 1H), 4.63 (s, 2H), 4.24 (dd, J = 7.0, 5.6 Hz, 1H), 4.19 (d, J = 2.4 Hz, 2H), 3.88–3.78 (m, 1H), 3.71–3.57 (m, 28H), 2.69 (s, 0H), 2.64 (ddd, J = 14.8, 9.0, 4.1 Hz, 1H), 2.48 (ddd, J = 14.7, 5.8, 3.4 Hz, 1H), 2.44 (s, 0H), 2.26 (td, J = 7.1, 5.8 Hz, 1H), 2.02 (s, 3H), 1.45 (d, J = 7.2 Hz, 3H), 1.26 (s, 1H), 0.99 (dd, J = 15.6, 6.9 Hz, 6H). Calculated for C₃₅H₆₁N₄O₁₂⁺ [M+NH₄] ⁺ 729.4; found 729.4.

*Synthesis of M11:* Starting with M10 (120 mg, 0.169 mmol) as the raw material, this was added to a 25 mL round-bottom flask and dissolved in a mixed solvent of 4 mL anhydrous dichloromethane and 400 μL anhydrous DMF. The mixture was cooled to –8 °C and, under stirring, sulfur dioxide dichloride dissolved in anhydrous dichloromethane (30.2 mg, 0.253 mmol) was slowly added. The reaction was maintained at –8 °C for 30 min, monitored by TLC. Once the reaction was complete, the solvent was removed using a rotary evaporator, and the product was purified by column chromatography, yielding a pale-yellow oily substance (75 mg, 61.48% yield). ¹H NMR (600 MHz, Chloroform-d) δ 8.70 (s, 1H), 7.73 (d, J = 8.2 Hz, 2H), 7.33–7.30 (m, 2H), 7.17–6.97 (m, 2H), 4.70–4.63 (m, 1H), 4.56 (s, 2H), 4.24–4.17 (m, 3H), 3.84 (dt, J = 9.8, 4.9 Hz, 1H), 3.72–3.52 (m, 28H), 3.35 (s, 0H), 2.67 (d, J = 11.1 Hz, 1H), 2.47 (ddd, J = 14.6, 5.5, 3.2 Hz, 1H), 2.43 (t, J = 2.4 Hz, 1H), 2.28 (h, J = 6.6 Hz, 1H), 1.45 (d, J = 7.2 Hz, 3H), 1.25 (s, 4H), 1.01 (dd, J = 15.9, 6.8 Hz, 6H). Calculated for C₃₅H₆₀ClN₄O₁₁⁺ [M+NH₄] ⁺ 747.3.; found 747.3.

*Synthesis of VA-dSA3:* Starting with dSA3 (20 mg, 20.9 mmol) and M11 (16.8 mg, 22.8 mmol) as raw materials, these were added to a 1.5 μL microcentrifuge tube and dissolved in 200 μL of anhydrous NMP. DIPEA (13.4 mg, 100.4 mmol) and TBAI (3.9 mg, 10.4 mmol) were added, and the reaction was maintained at 60 °C for 14 days, monitored by TLC and liquid chromatography. Once the reaction was complete, the solvent was removed using a rotary evaporator, and the product (quaternary ammonium chloride or iodide) was purified by column chromatography and then lyophilized to obtain a white solid (14.7 mg, 45.65% yield). Purity > 95%.1H-NMR (600 MHz, DMSO-d6): δ 10.289-10.191 (m, 1H), 8.235 (s, 1H), 7.873-7.861 (m, 2H), 7.761-7.735 (m, 3H), 7.506-7.303 (m, 4H), 6.647 (s, 1H), 6.513-6.435 (s, 1H), 5.325 (t, J = 4.8 Hz, 1H), 4.999-4.855 (m, 3H), 4.593-4.374 (m, 5H), 4.201-4.189 (m, 2H), 4.132 (s, 3H), 3.589-3.413 (m, 43H), 2.109-2.099 (m, 4H), 2.021-1.943 (m, 6H), 1.323-1.293 (m, 7H), 0.884-0.817 (m, 12H). Calculated for C₈₃H₁₁₅N₁₃O₂₁⁺ [M+H] ⁺ 1543.8; found 1543.778.

*Generation of the TZ-dSA3-12:* Anti-HER2 antibody Trastuzumab in HEPES buffer (20 mM, pH ≈ 7) was treated with NHS-PEG₄-Azide (~4 equivalents relative to the whole antibody) for 12 h. To the reduced antibody were added the desired compounds VA-dSA3 with the maleimide group (15 equivalents) in dimethylacetamide for 24 h. The conjugates were exchanged into HEPES buffer (pH = 7) using Sephadex G-25S to remove the free 1–17 to give the HEPES ammonium salt TZ-dSA3-12 and stored at –80 °C before use for analysis and testing.

*Generation of the TZ-dSA3-12-FITC:* FITC NHS ester was purchased from Innochem Company. The FITC-labeled conjugates were prepared according to the manufacturer's instructions. Specifically, the conjugates were treated with the corresponding dye in PBS (pH = 7.4) for 4 h. The excitation wavelength for the FITC-labeled conjugates is 492 nm, and the emission wavelength is 518 nm.

*Synthesis of the TZ-non-cleavable dSA3:* The synthetic route for TZ-non-cleavable dSA3 is depicted in the Supporting Information Appendix, Scheme S3.

**Scheme S3.** Synthesis of antibody conjugates TZ-non-cleavable-dSA3. Reagents and conditions: (a) PAB, EDCI, HOBt, DIPEA, anhydrous DMF, r.t, 18 h; (b) SOCl_2_, -15 ºC, 1 h; (c) dSA3, TBAI, DIPEA, 55 ºC, 7 d; (d) TZ, TCEP, NAC, H_2_O, r.t.

*Synthesis of M12:* Maleimide-octoethylene glycol-carboxylic acid (500.00 mg, 0.84 mmol) was added to a 100 mL round-bottom flask and dissolved in 20 mL of anhydrous DMF. At room temperature with stirring, EDCI (241.54 mg, 1.26 mmol), HOBt (136.47 mg, 1.01 mmol), and DIPEA (162.72 mg, 1.26 mmol) were added sequentially. The reaction was carried out at room temperature under an argon atmosphere for 2 h. PAB (103.45 mg, 0.84 mmol) was then added to the reaction mixture. The progress of the reaction was monitored by TLC. After the reaction was complete, the solvent was removed using a rotary evaporator, and the product was purified by column chromatography to obtain a colorless oily substance (500.85 mg, 85.45% yield). ¹H NMR (600 MHz, DMSO-d₆) δ 10.02 (s, 1H), 8.02 (t, J = 5.5 Hz, 1H), 7.62–7.53 (m, 2H), 7.35 (d, J = 8.5 Hz, 1H), 7.22 (dd, J = 8.4, 4.4 Hz, 1H), 7.00 (s, 2H), 4.71 (s, 1H), 4.33 (s, 1H), 3.69 (t, J = 6.2 Hz, 2H), 3.59 (t, J = 7.3 Hz, 3H), 3.52–3.47 (m, 33H), 3.36 (t, J = 5.9 Hz, 3H), 3.25 (s, 1H), 3.14 (q, J = 5.8 Hz, 3H), 2.58–2.53 (m, 2H), 2.33 (t, J = 7.3 Hz, 2H). Calculated for C₃₃H₅₂N₃O₁₃⁺ [M+H] ⁺ 698.3422; found 698.3390.

*Synthesis of M13:* Starting with intermediate M12 (348.89 mg, 0.50 mmol), this was added to a 100 mL round-bottom flask and dissolved in a mixed solvent of 5 mL anhydrous DMF and 15 mL anhydrous dichloromethane. The mixture was cooled to –15 °C and stirred while thionyl chloride (178.46 mg, 1.50 mmol) dissolved in 5 mL anhydrous dichloromethane was slowly added. The reaction was maintained at –15 °C for 30 min, monitored by TLC. After the reaction was complete, the solvent was removed using a rotary evaporator, and the product was purified by column chromatography to obtain a pale-yellow oily substance (154.85 mg, 43.24% yield). ¹H NMR (600 MHz, DMSO-d₆) δ 10.04 (s, 1H), 8.01 (t, J = 5.3 Hz, 1H), 7.60 (d, J = 8.4 Hz, 2H), 7.35 (d, J = 8.4 Hz, 2H), 7.00 (s, 2H), 4.71 (s, 1H), 3.69 (t, J = 6.2 Hz, 2H), 3.60 (d, J = 7.2 Hz, 2H), 3.49 (dd, J = 9.9, 4.6 Hz, 28H), 3.37 (d, J = 4.1 Hz, 2H), 3.25 (s, 1H), 3.14 (dd, J = 11.4, 5.7 Hz, 2H), 2.57–2.54 (m, 2H), 2.33 (t, J = 7.3 Hz, 2H). Calculated for C₃₃H₅₀ClN₃O₁₂⁺ [M+H] ⁺ 716.3083; found 716.3153.

*Synthesis of M14:* dSA3 (20 mg, 23.53 mmol) and intermediate M13 (18.54 mg, 25.88 mmol) were added to a 1.5 μL microcentrifuge tube and dissolved in 300 μL of anhydrous DMF. DIPEA (3.49 mg, 25.88 mmol) and TBAI (4.35 mg, 11.77 mmol) were then added. The reaction was maintained at 55 °C for 7 days, monitored by TLC. After the reaction was complete, the solvent was removed using a rotary evaporator, and the product (quaternary ammonium chloride or iodide) was purified by column chromatography to obtain a white solid (14.40 mg, 39.98% yield). Purity > 95%. Calculated for C₇₅H₁₀₁N₁₆O₁₉⁺ [M+H]⁺ 1530.7423; found 1529.7416.

*Generation of the TZ-non-cleavable dSA3:* The synthesis and purification method of the quaternary ammonium chloride TZ-non-cleavable dSA3 is similar to that of TZ-dSA3-2, with intermediate M7 replaced by M14.

The FITC NHS ester and DyLight 680 NHS ester were obtained commercially from Innochem and Thermo Fisher Scientific, respectively. FITC-labeled and DyLight 680-labeled conjugates were synthesized following the manufacturer's instructions. In particular, the conjugates were incubated with the respective dyes in NaHCO₃ buffer (pH = 8.3) for 3 h, and then transferred to PBS buffer to produce the dye-labeled conjugates. The FITC-labeled conjugate exhibited an excitation wavelength of 492 nm and an emission wavelength of 518 nm, while the DyLight 680-labeled conjugate had an excitation wavelength of 692 nm and an emission wavelength of 712 nm.


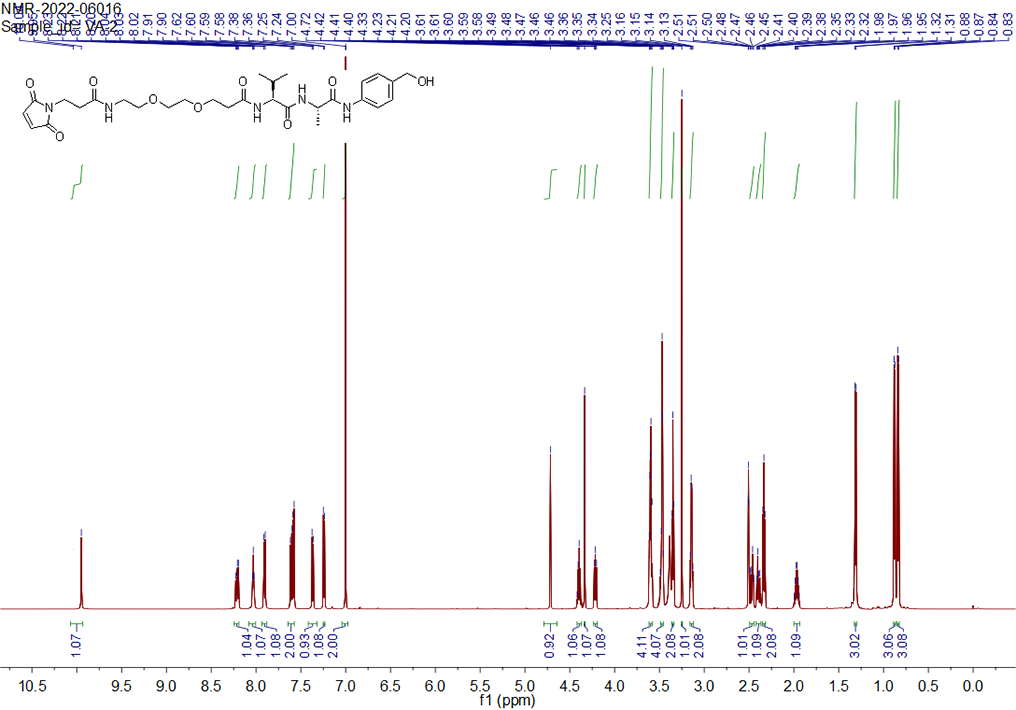


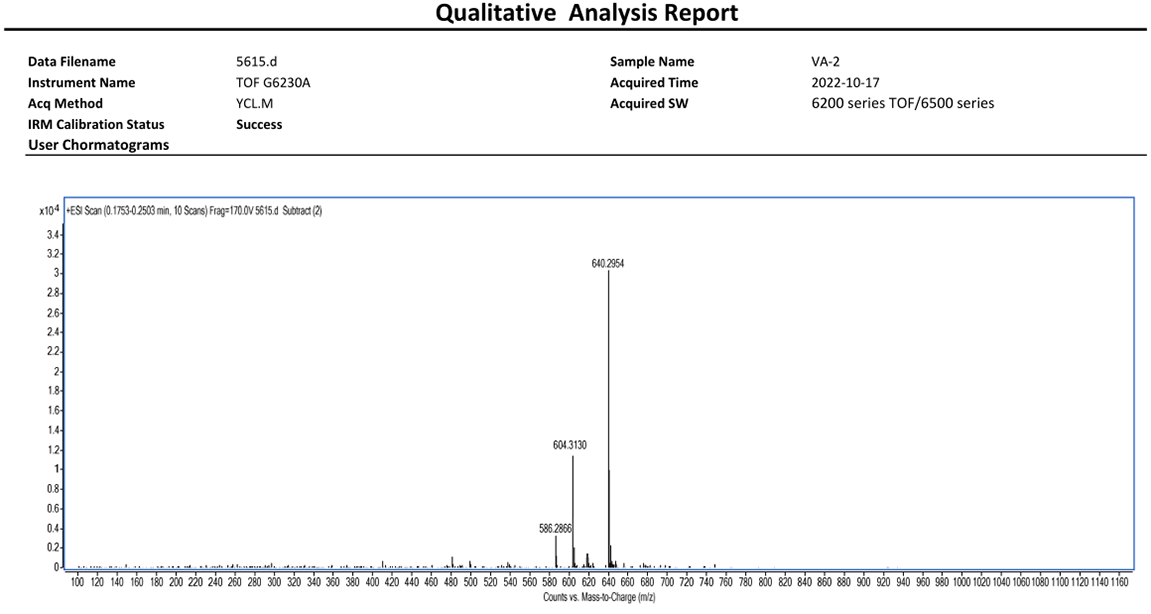


**Figure S11.** The 1H-NMR spectrum and MS spectrum of M1


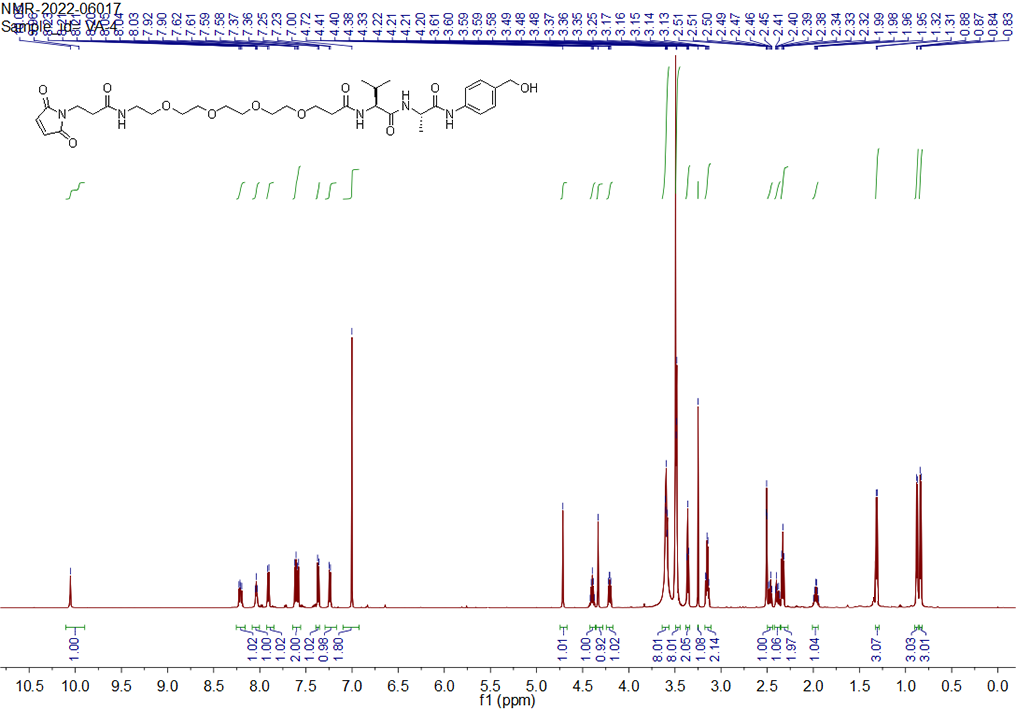


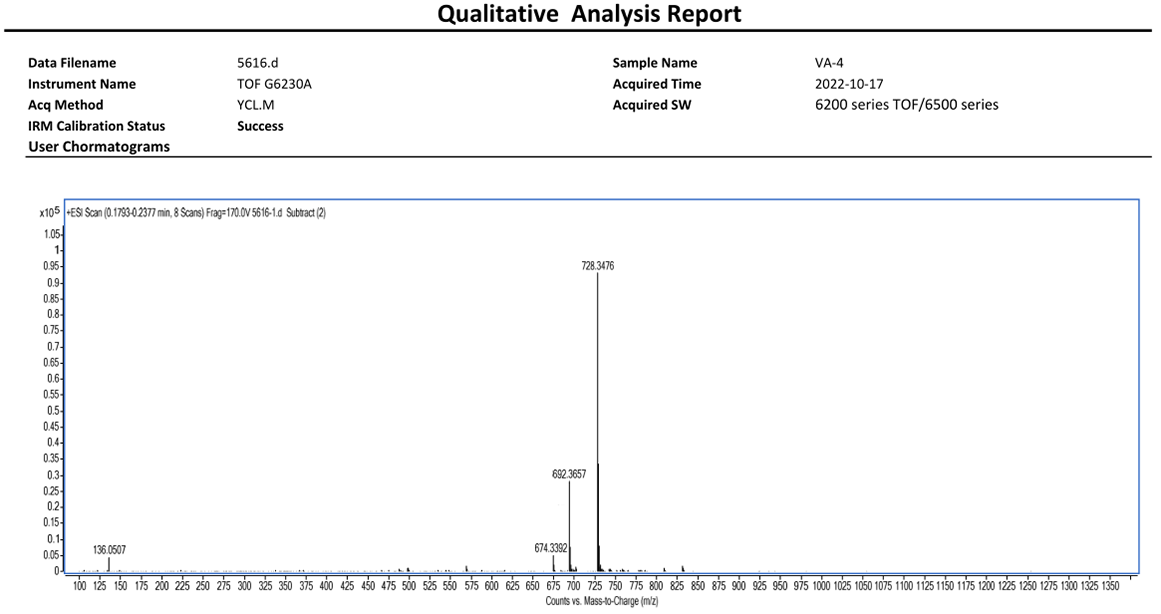


**Figure S12.** The 1H-NMR spectrum and MS spectrum of M2


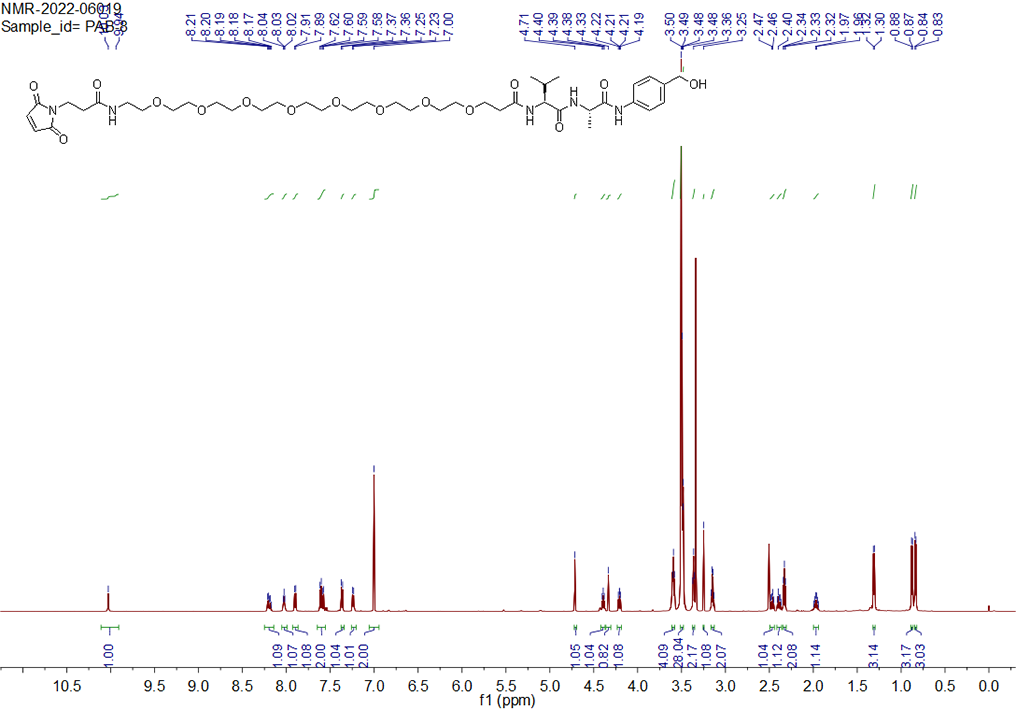


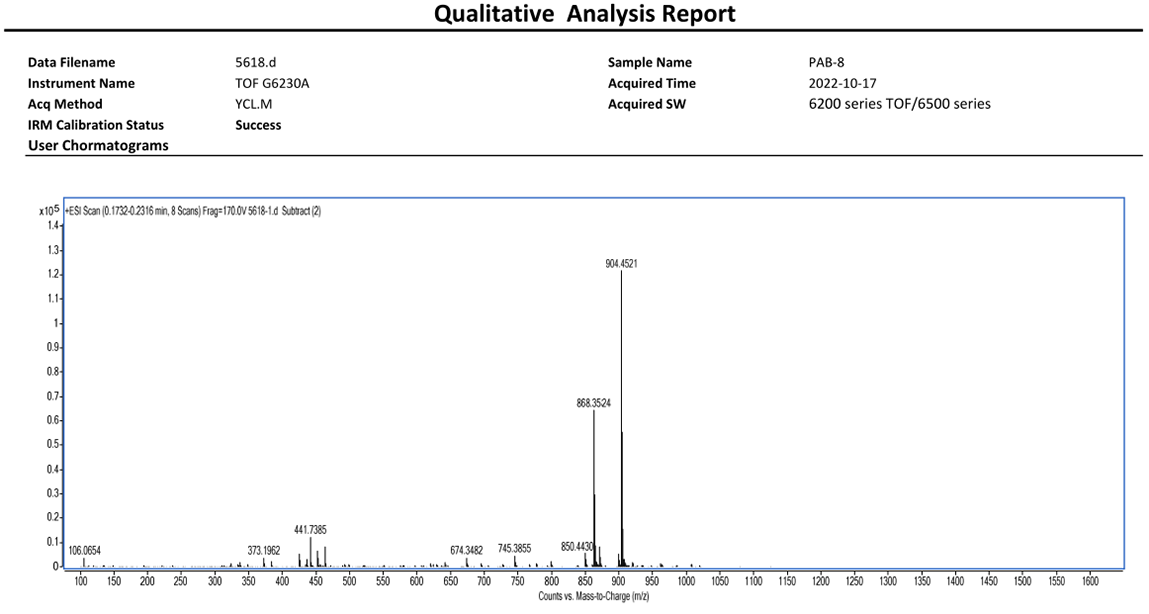
**Figure S13.** The 1H-NMR spectrum and MS spectrum of M3


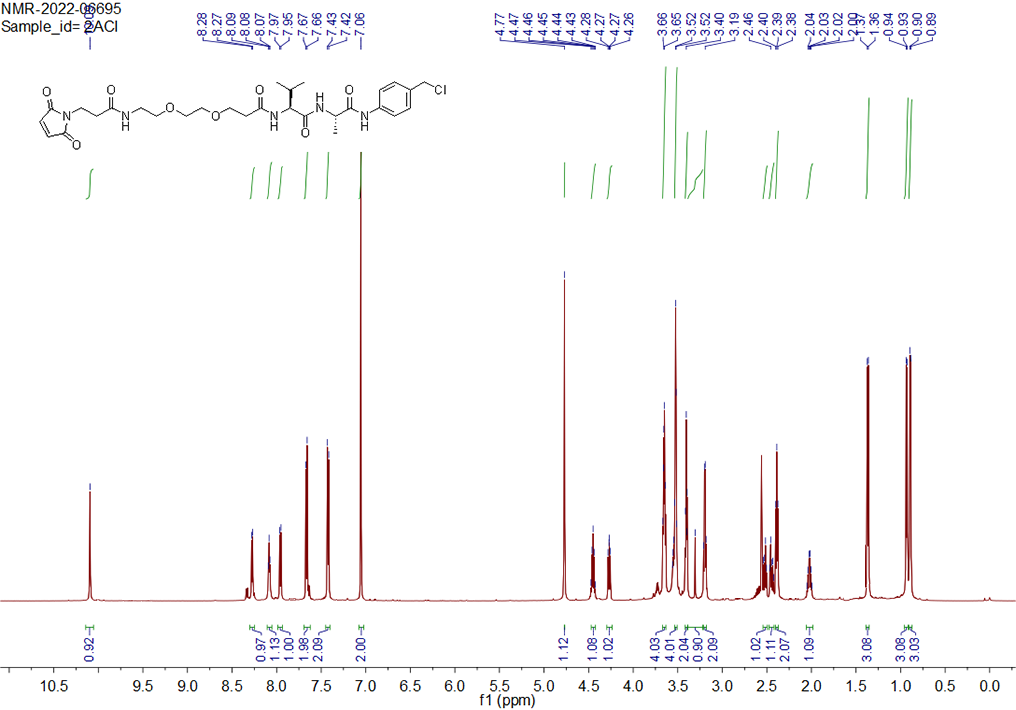


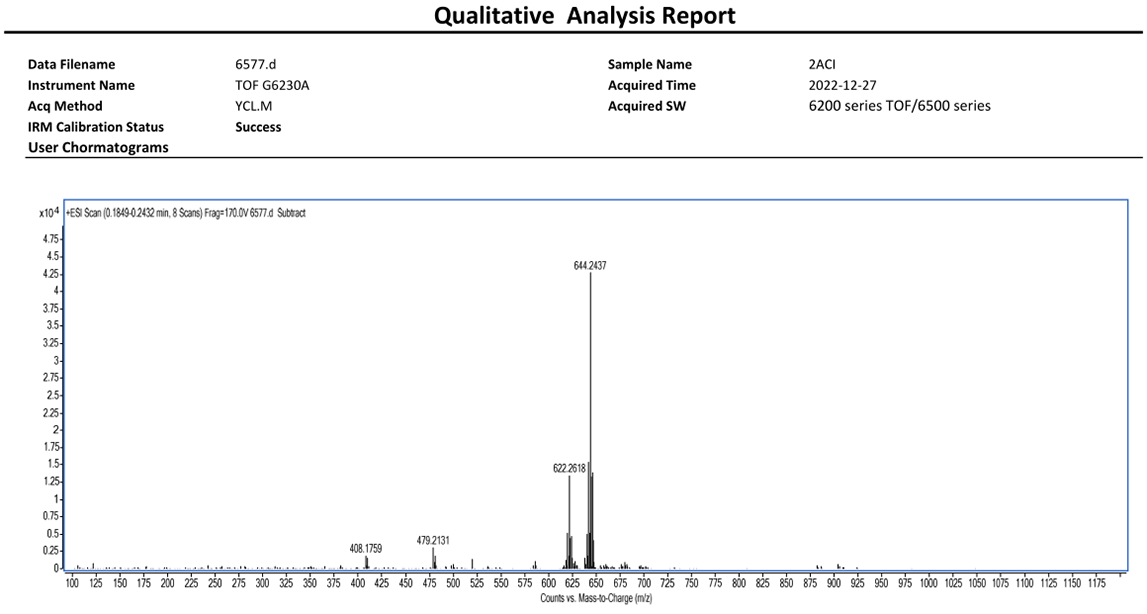


**Figure S14.** The 1H-NMR spectrum and MS spectrum of M4


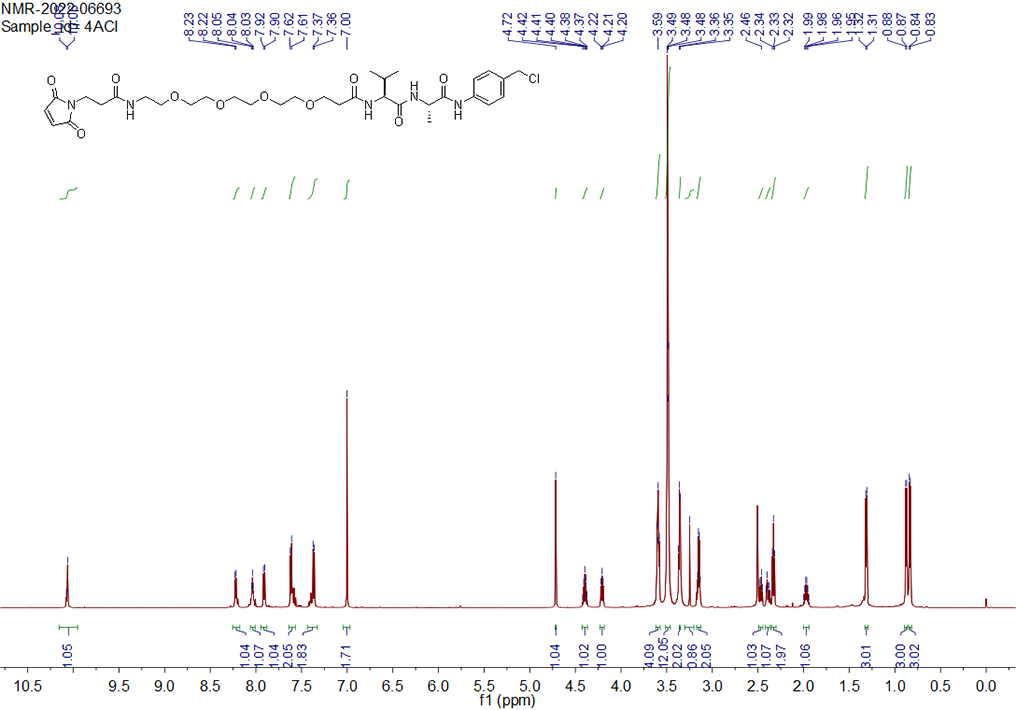


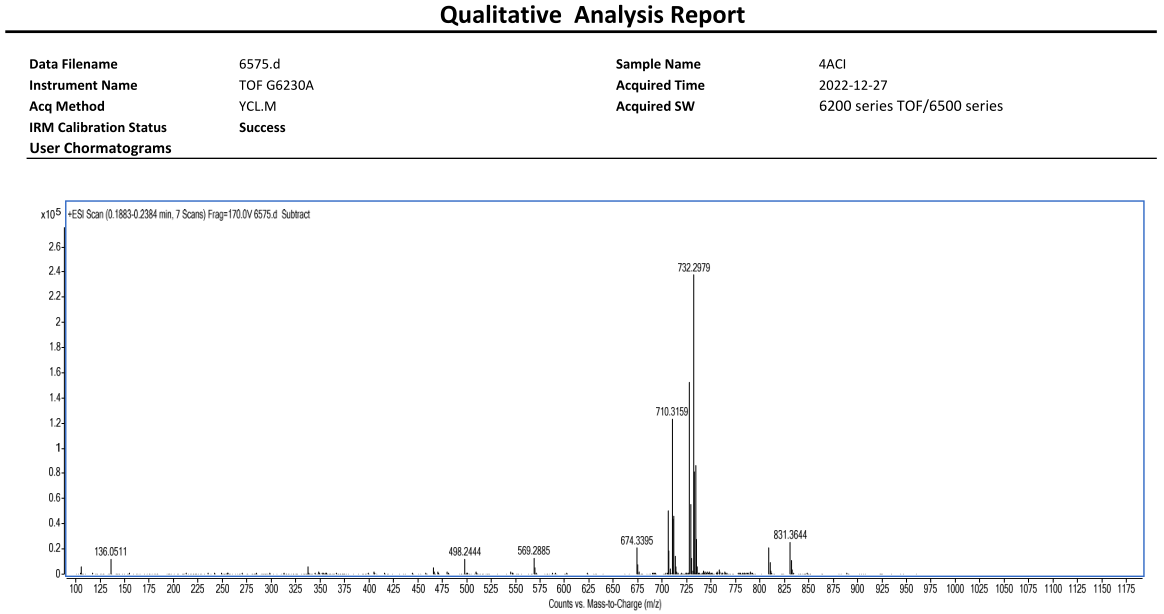
**Figure S15.** The 1H-NMR spectrum and MS spectrum of M5


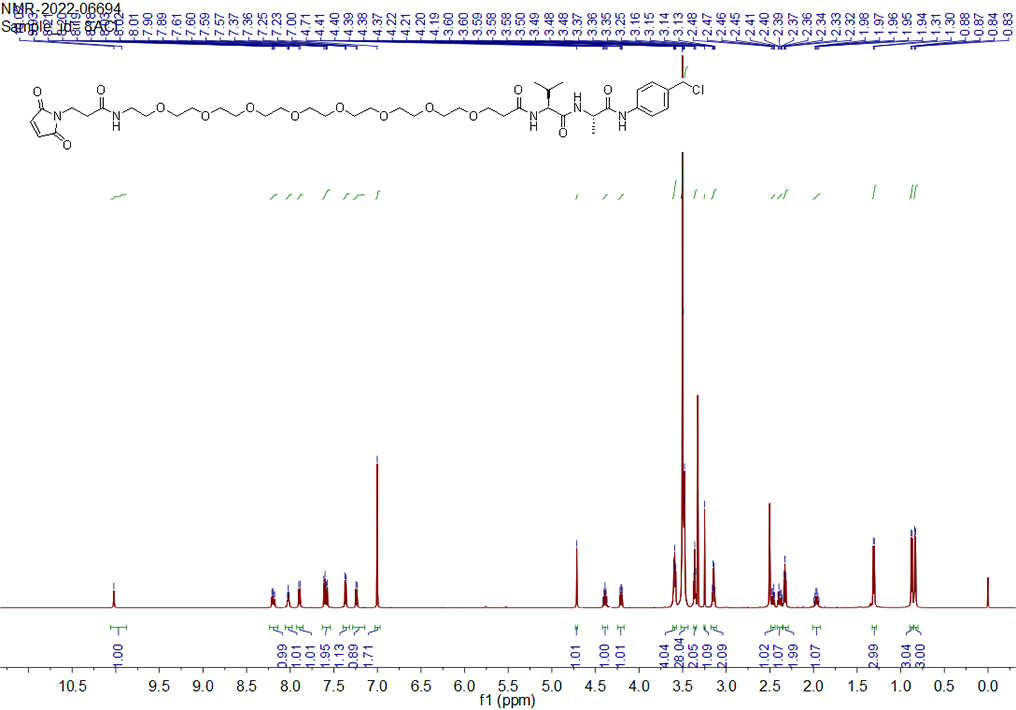


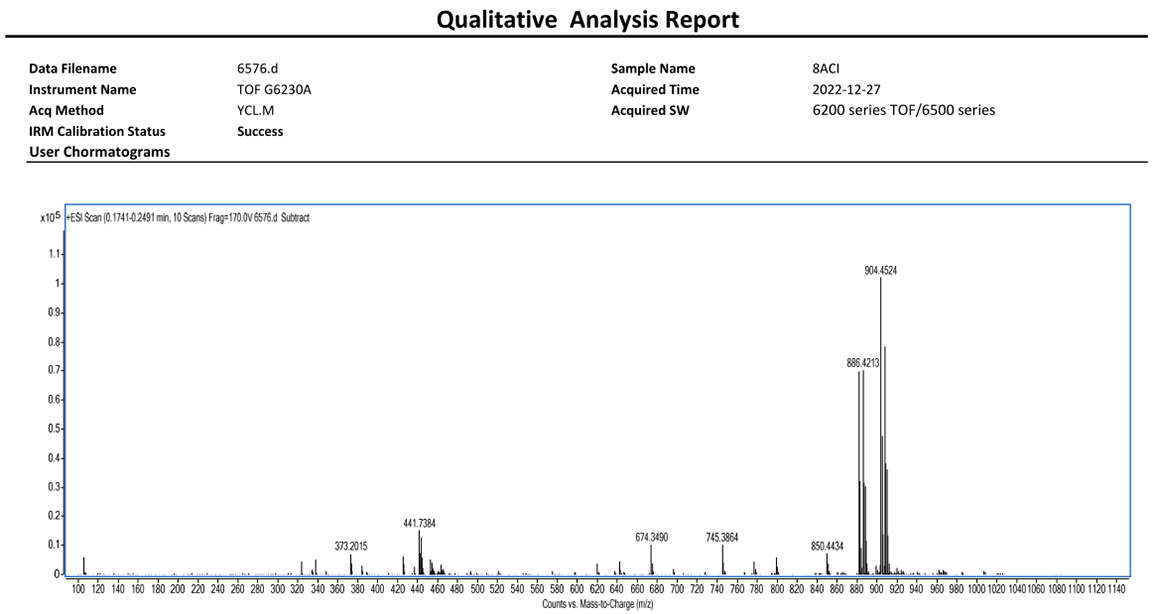
**Figure S16.** The 1H-NMR spectrum and MS spectrum of M6


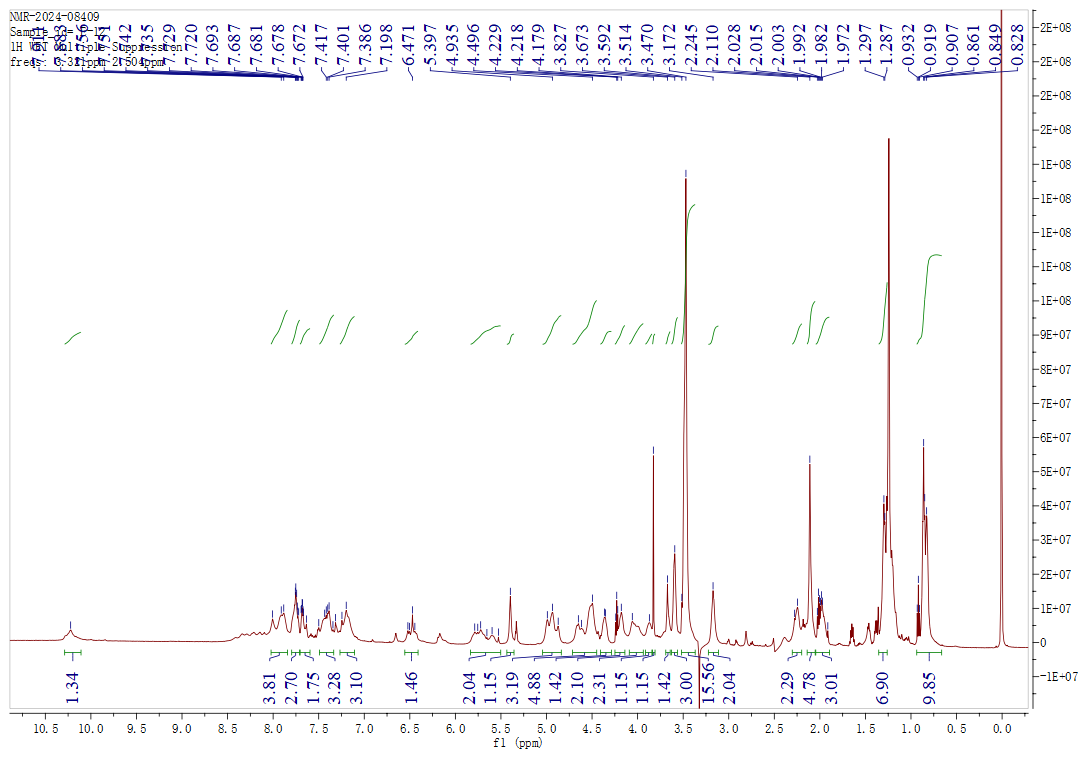

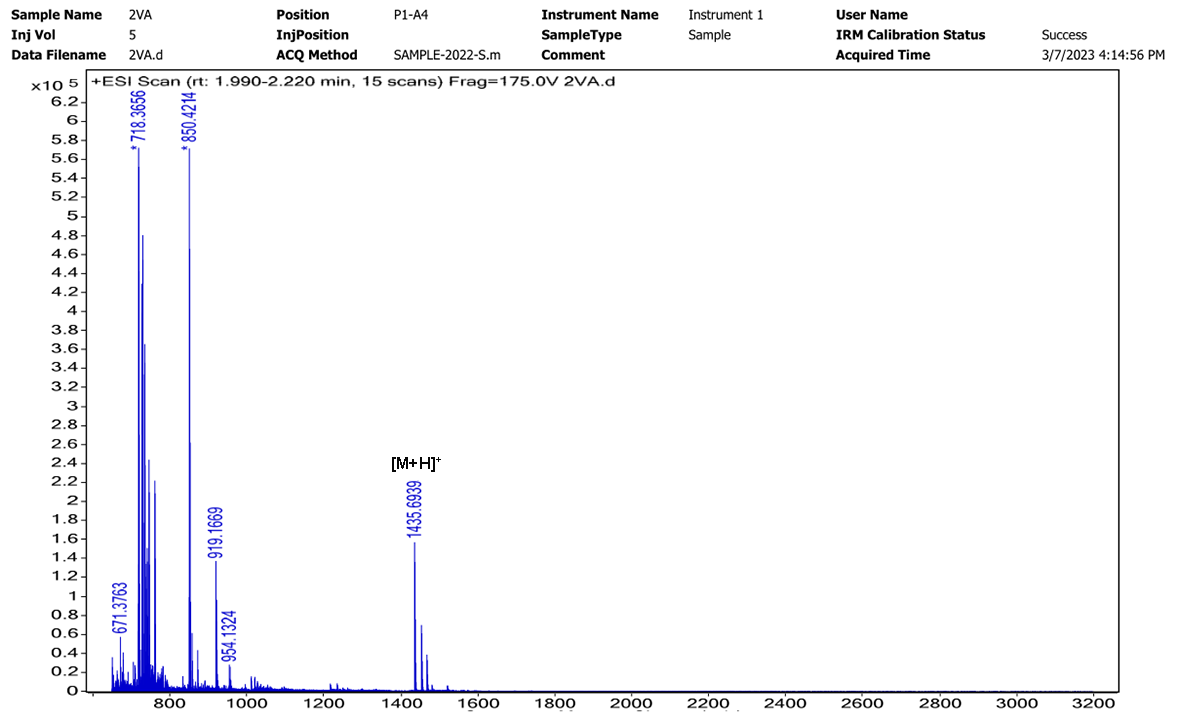
**Figure S17.** The HPLC spectrum and MS spectrum of M7


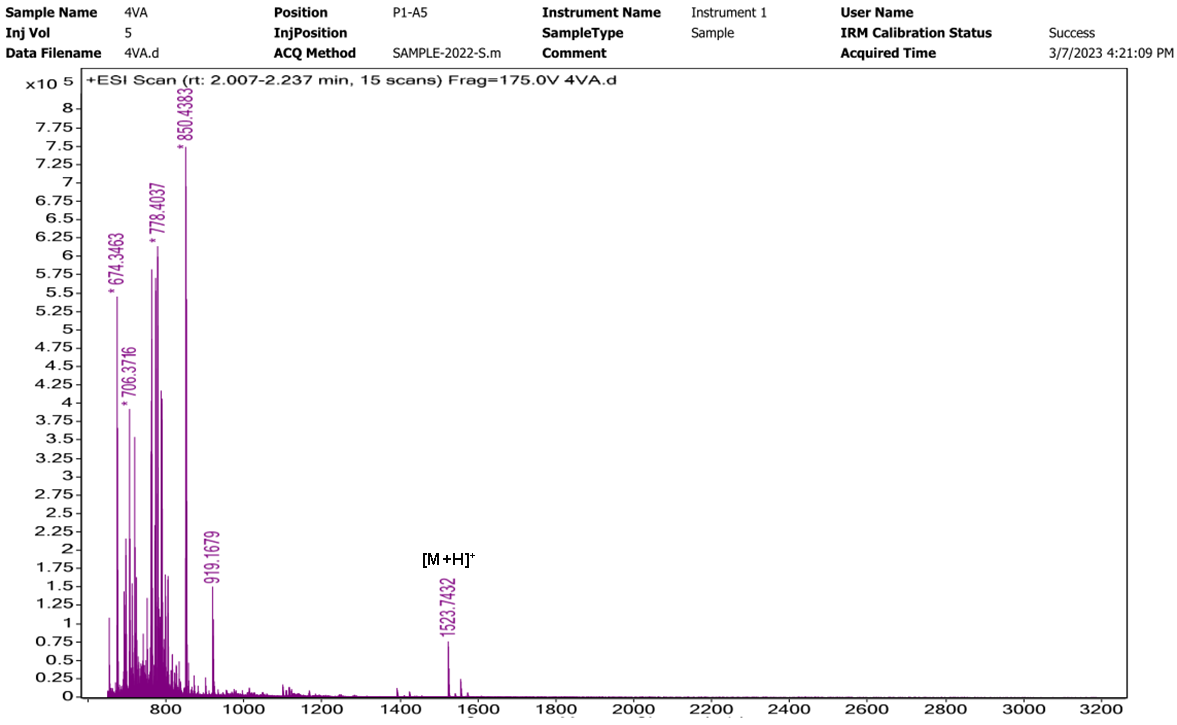
**Figure S18.** The MS spectrum of M8


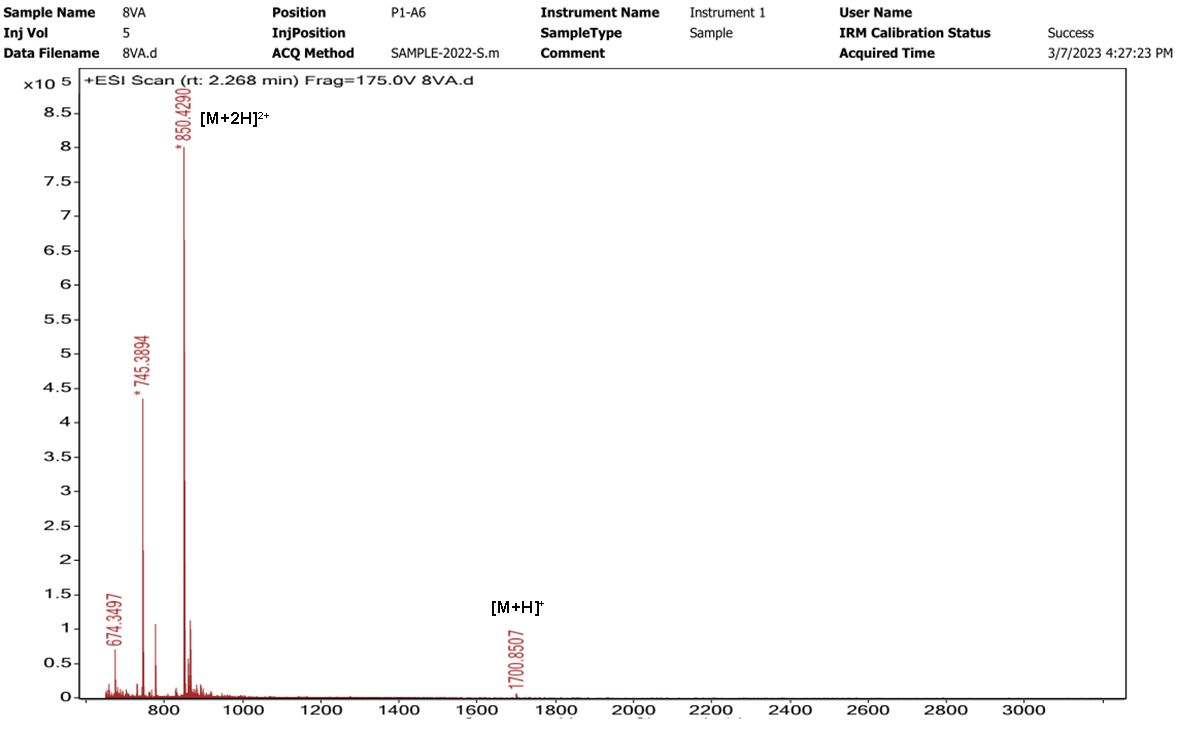


**Figure S19.** The MS spectrum of M9

**
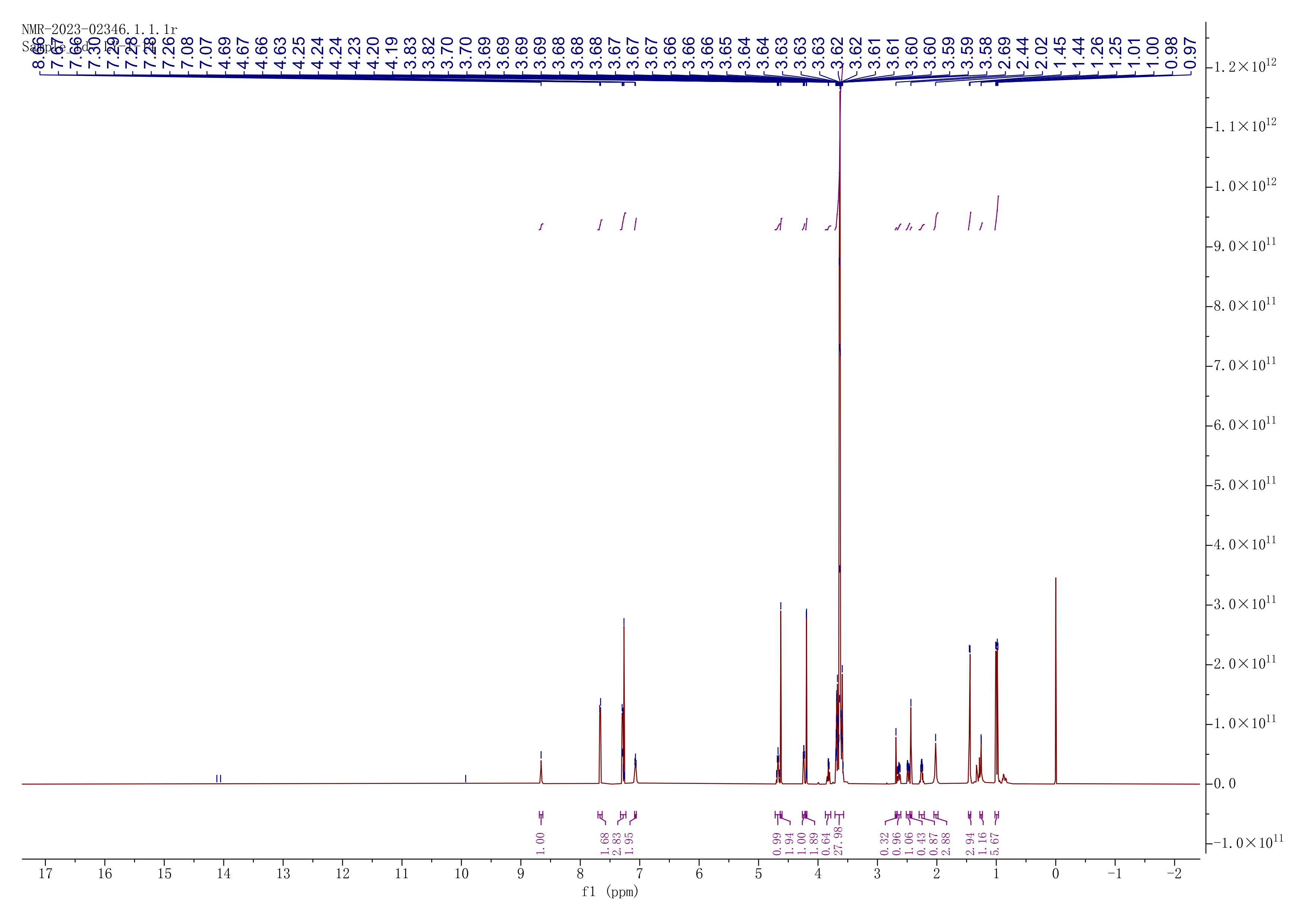
**

**
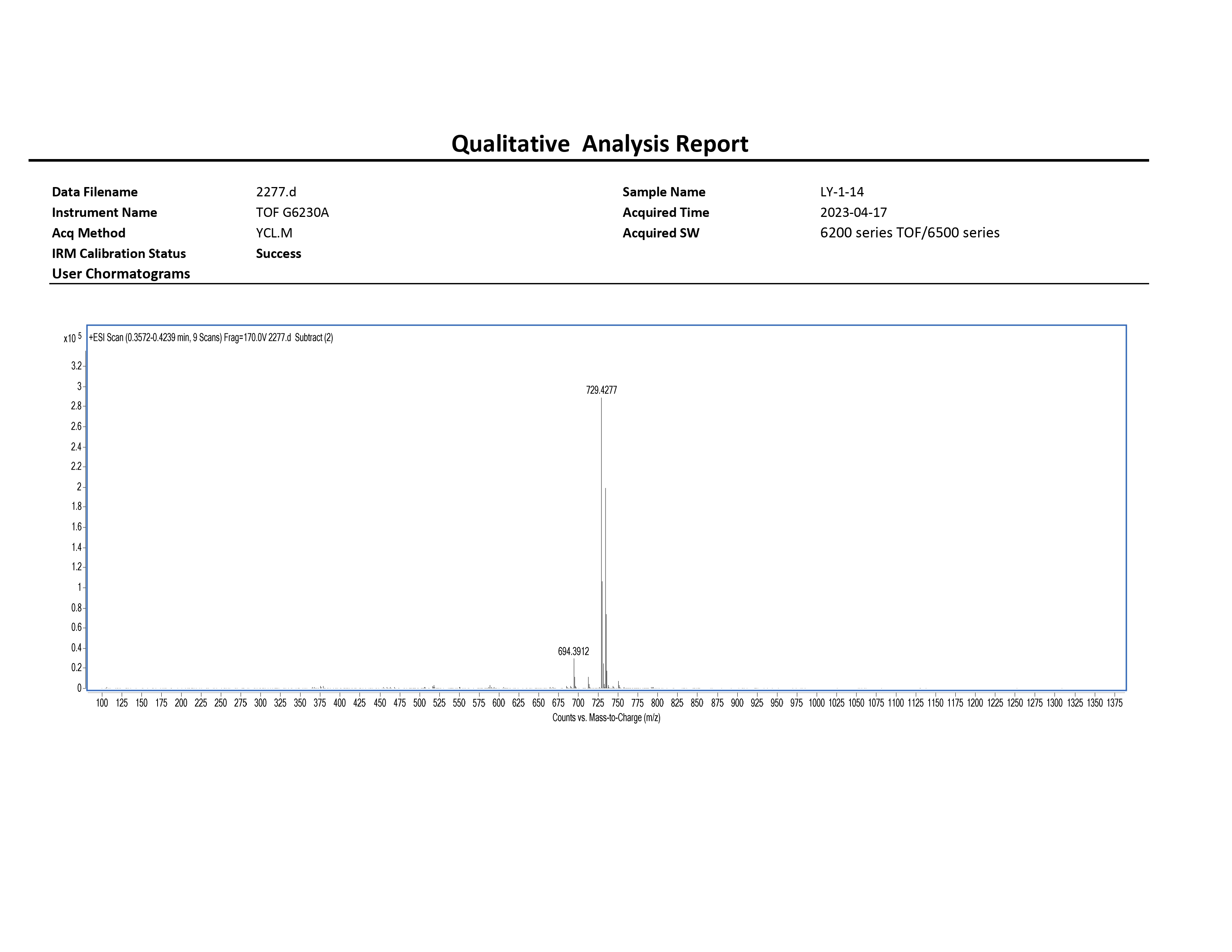
Figure S20.** The 1H-NMR spectrum and MS spectrum of M10

**
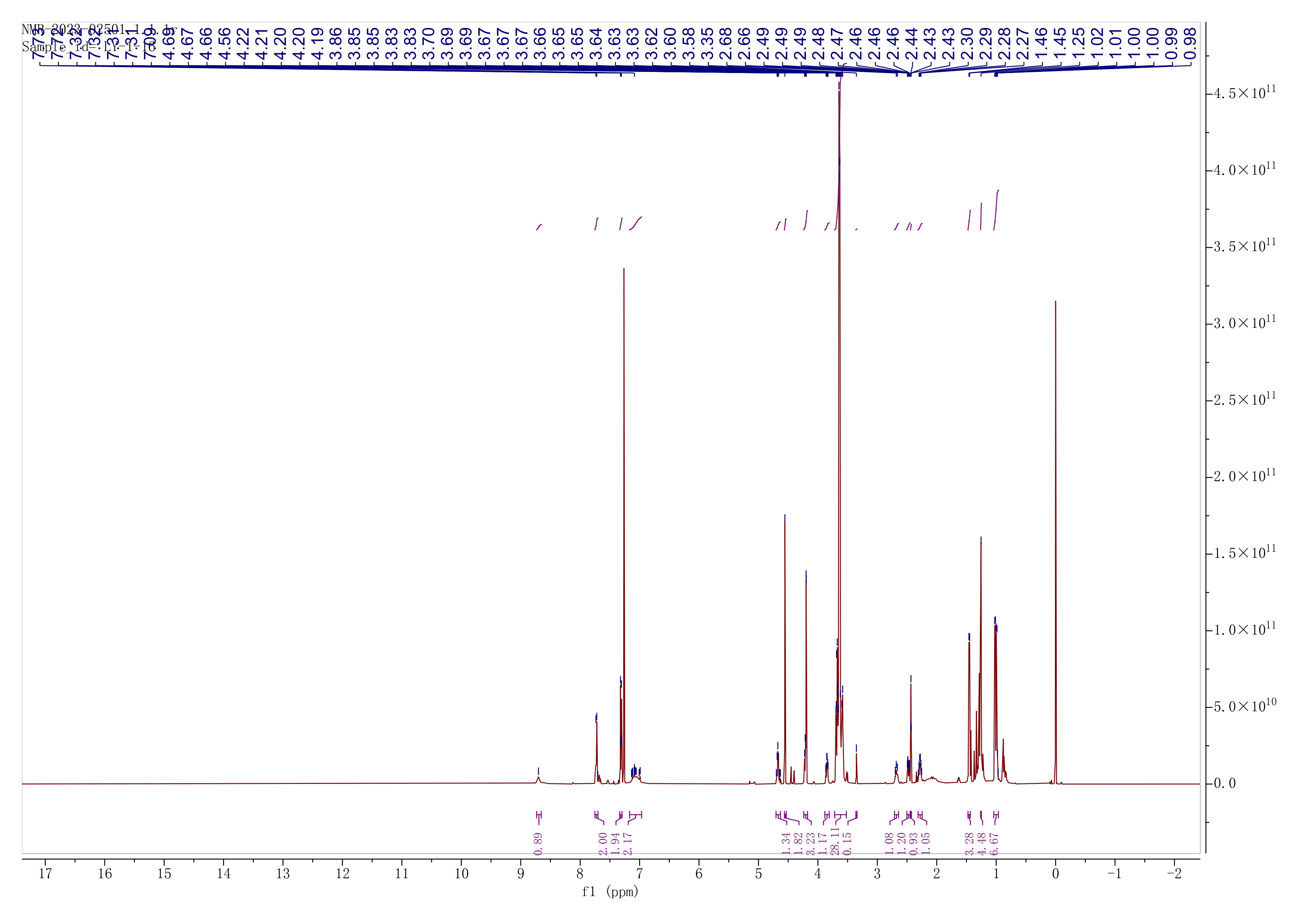
**

**
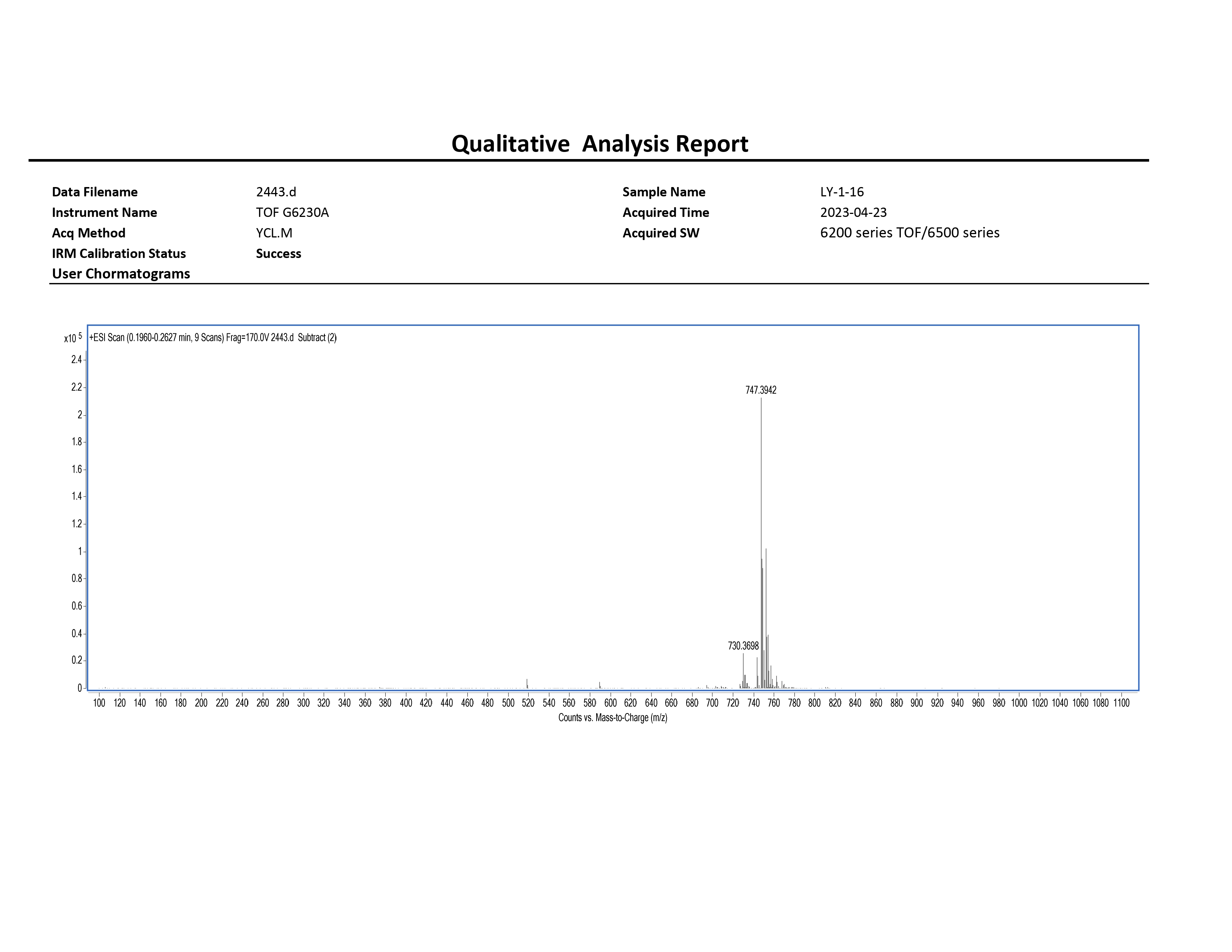
**

**Figure S21.** The 1H-NMR spectrum and MS spectrum of M11


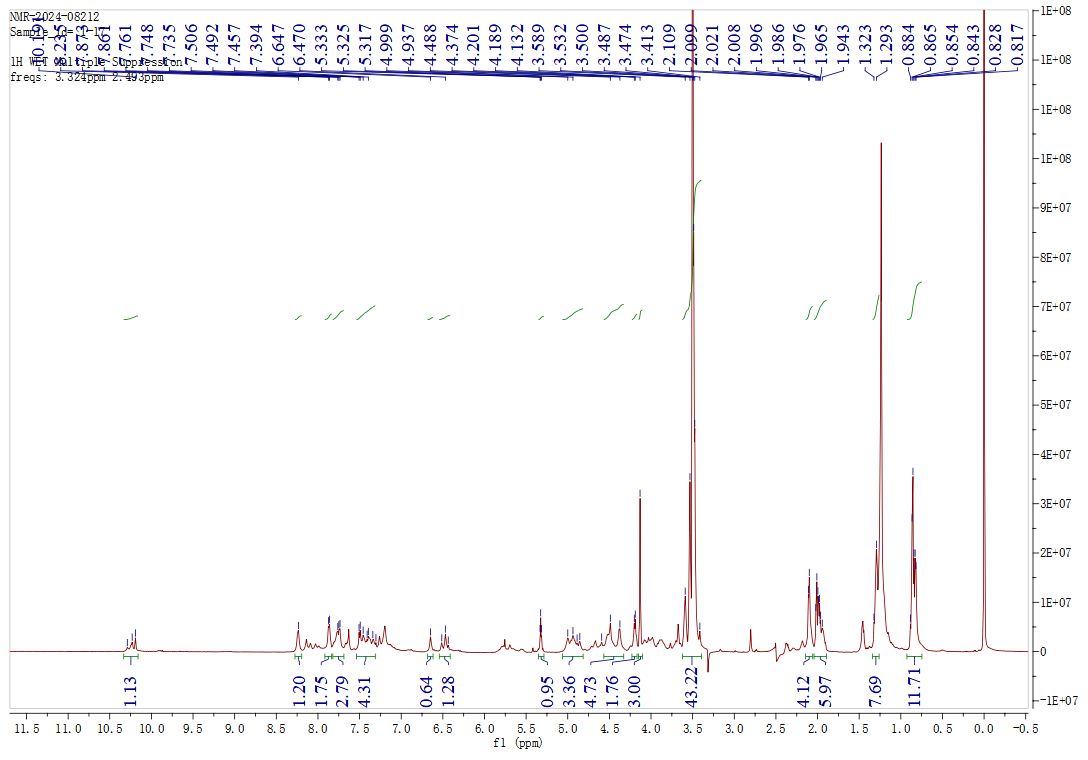


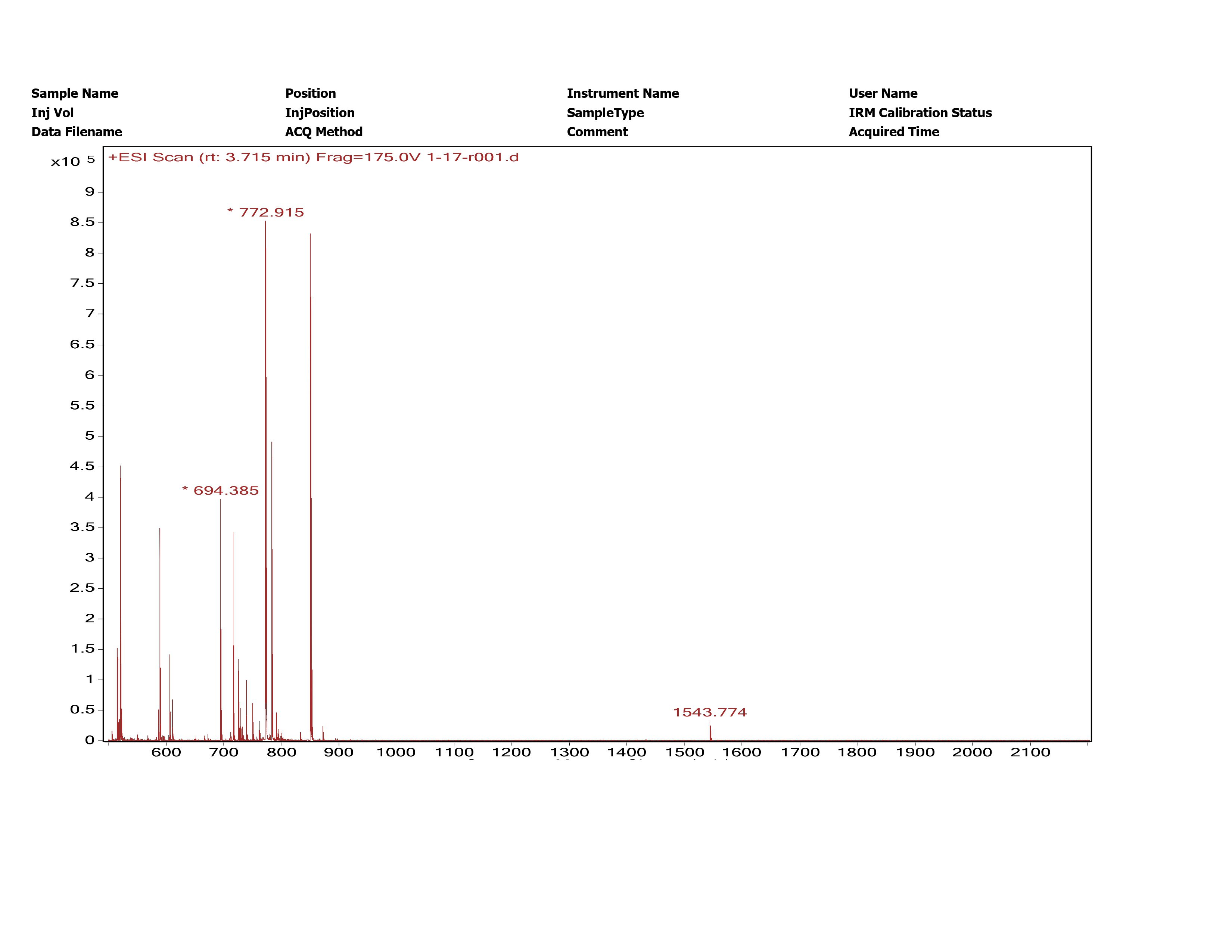


**Figure S22.** The 1H-NMR spectrum and MS spectrum of VA-dSA3


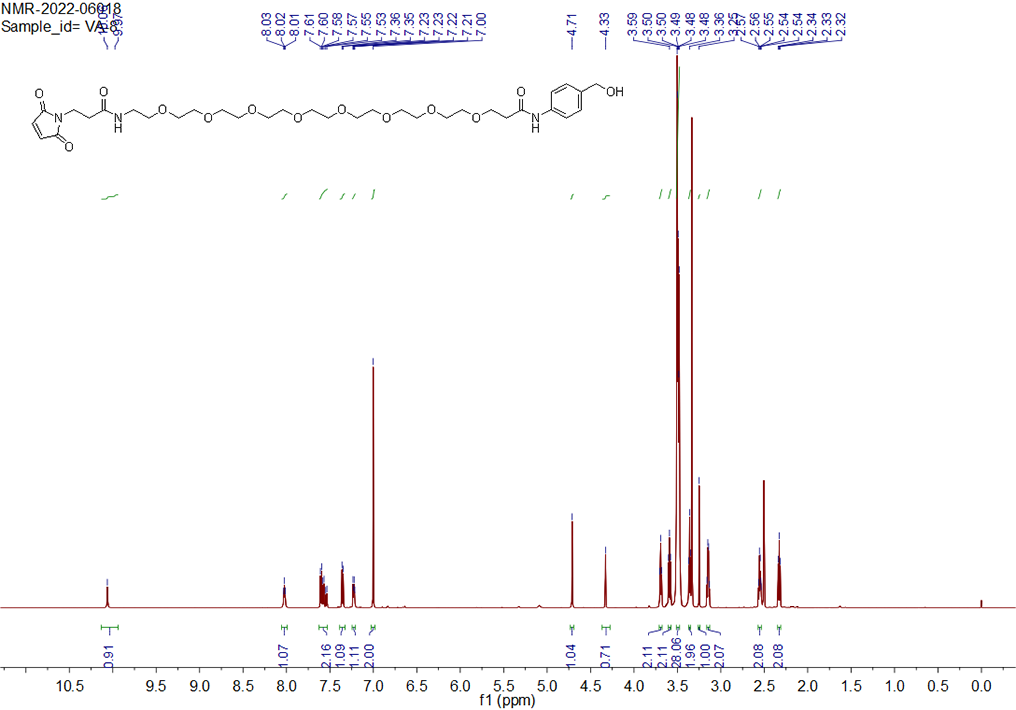


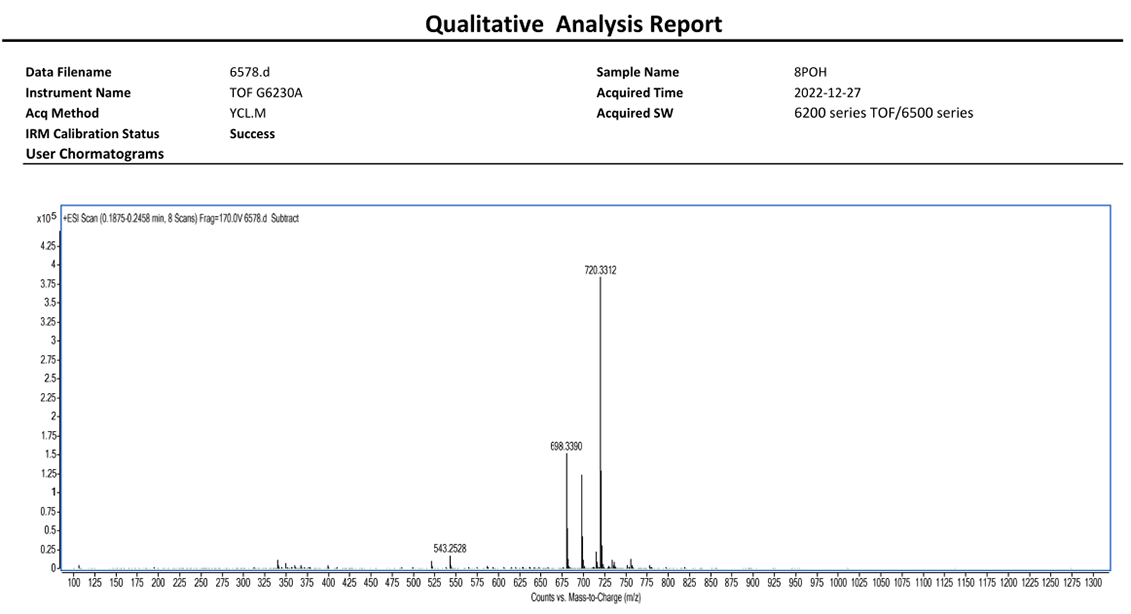
**Figure S23.** The 1H-NMR spectrum and MS spectrum of M12


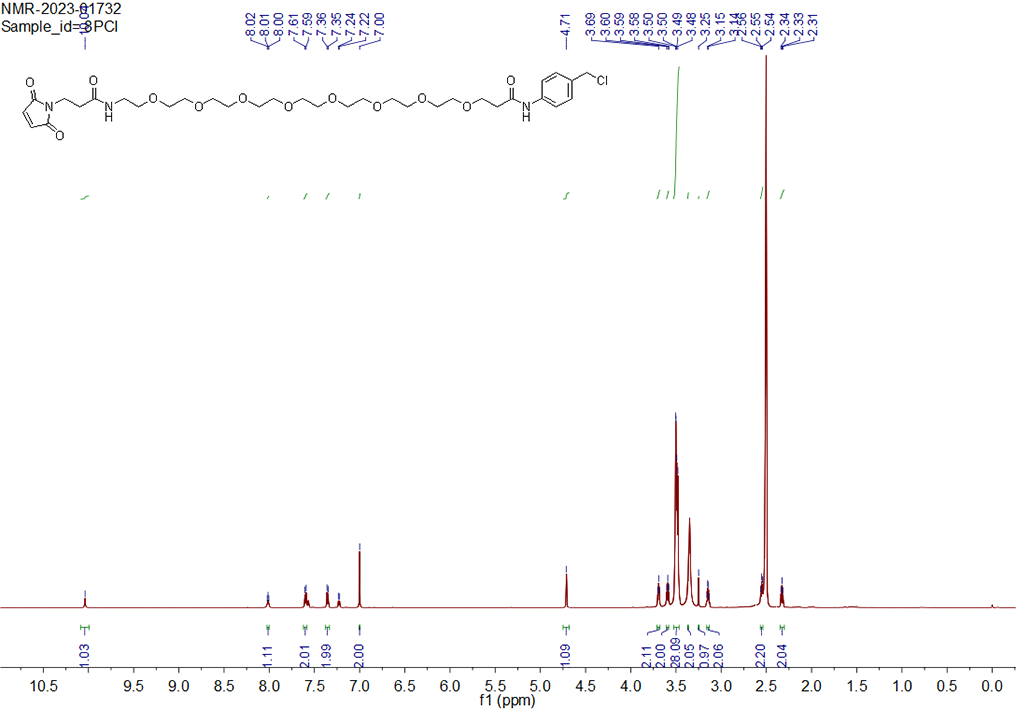


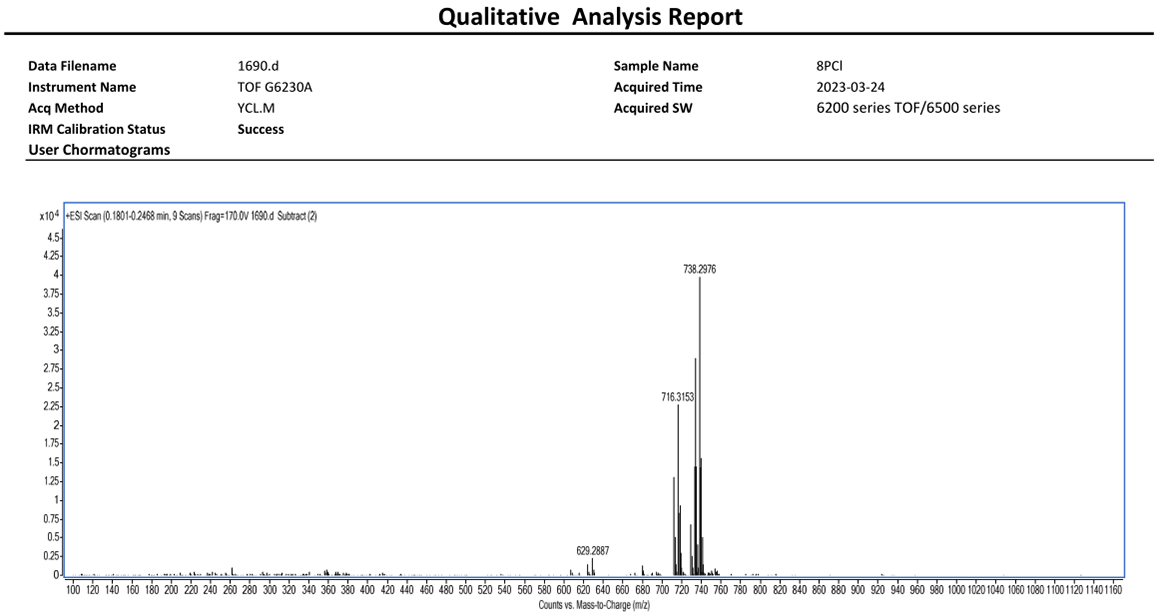
**Figure S24.** The 1H-NMR spectrum and MS spectrum of M13


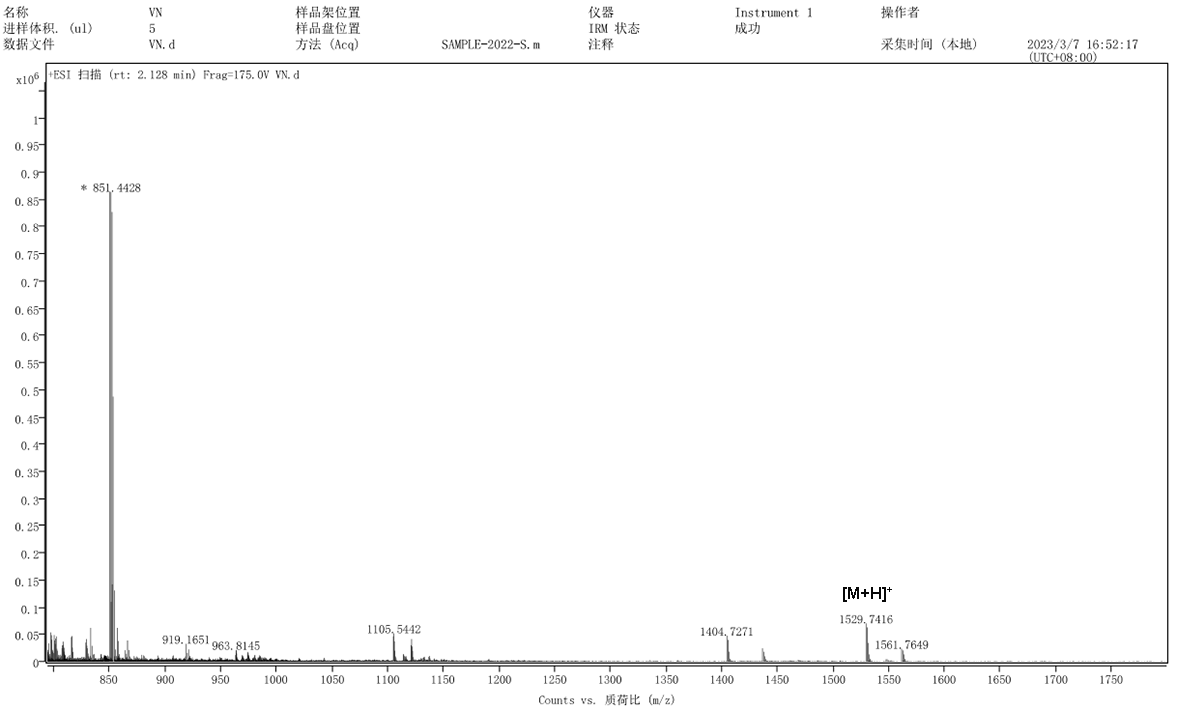


**Figure S25.** The MS spectrum of M14
